# Development and interpretation of a multimodal predictive model for prognosis of gastrointestinal stromal tumor

**DOI:** 10.1038/s41698-024-00636-4

**Published:** 2024-07-26

**Authors:** XianHao Xiao, Xu Han, YeFei Sun, GuoLiang Zheng, Qi Miao, YuLong Zhang, JiaYing Tan, Gang Liu, QianRu He, JianPing Zhou, ZhiChao Zheng, GuiYang Jiang, He Song

**Affiliations:** 1 Department of Gastrointestinal Surgery, The First Hospital of China Medical University, Shenyang, Liaoning China; 2 Department of Pathology, The First Hospital and the College of Basic Medical Sciences of China Medical University, Shenyang, Liaoning China; 3 Department of Gastric Surgery, Cancer Hospital of China Medical University; Cancer Hospital of Dalian University of Technology, Liaoning Cancer Hospital & Institute, Shenyang, Liaoning China; 4 Department of Radiology, The First Hospital of China Medical University, Shenyang, Liaoning China; 5 The state Key laboratory of Neurology and Oncology Drug Development, Jiangsu Simcere Diagnostics Co.,Ltd, Nanjing, China; 6 Department of Pathology, The College of Basic Medical Sciences and The First Hospital of China Medical University, Shenyang, Liaoning China

**Keywords:** Sarcoma, Risk factors

## Abstract

Gastrointestinal stromal tumor (GIST) is the most common mesenchymal original tumor in gastrointestinal (GI) tract and is considered to have varying malignant potential. With the advancement of computer science, radiomics technology and deep learning had been applied in medical researches. It’s vital to construct a more accurate and reliable multimodal predictive model for recurrence-free survival (RFS) aiding for clinical decision-making. A total of 254 patients underwent surgery and pathologically diagnosed with GIST in The First Hospital of China Medical University from 2019 to 2022 were included in the study. Preoperative contrast enhanced computerized tomography (CE-CT) and hematoxylin/eosin (H&E) stained whole slide images (WSI) were acquired for analysis. In the present study, we constructed a sum of 11 models while the multimodal model (average C-index of 0.917 on validation set in 10-fold cross validation) performed the best on external validation cohort with an average C-index of 0.864. The multimodal model also reached statistical significance when validated in the external validation cohort (*n* = 42) with a p-value of 0.0088 which pertained to the recurrence-free survival (RFS) comparison between the high and low groups using the optimal threshold on the predictive score. We also explored the biological significance of radiomics and pathomics features by visualization and quantitative analysis. In the present study, we constructed a multimodal model predicting RFS of GIST which was prior over unimodal models. We also proposed hypothesis on the correlation between morphology of tumor cell and prognosis.

## Introduction

Gastrointestinal stromal tumor (GIST) is the most prevalent mesenchymal tumor originating in the gastrointestinal (GI) tract^1^. GISTs are recognized for varying degrees of malignant potential^2^. Tumor size, mitotic number, tumor original site and tumor rupture are widely acknowledged as key factors of the risk of recurrence and metastasis assessed by modified National Institutes of Health (NIH) scale^3^. For patients at high risk of recurrence, tyrosine kinase inhibitors (TKI) is regularly recommended as adjuvant therapy for GISTs^4^. However, a critical concern in clinical practice is the accurate estimation of recurrence risk. An overestimation of the risk could lead to unnecessary financial burden and potentially adverse effects, while an underestimation might result in inadequate treatment and negatively impact the patient’s prognosis^5,6^. In light of these considerations, it becomes imperative to develop a more precise and reliable predictive model, especially concerning recurrence-free survival (RFS).

With the continuous advancement of feature engineering and imaging processing techniques, radiomics technology now offers a microscopic view into the understanding of tumor imaging by extracting and analyzing a wealth of features derived from fine texture and pixel distributions^7,8^. Concurrently, deep learning (DL) technology has become a common tool in the medical field. There is compelling evidence demonstrating the effectiveness of DL in tasks such as tissue classification, semantic segmentation and disease diagnosis^9–12^. Unlike radiomics, DL learns directly from the images without the need for predefined features. While the “black box” nature of DL models during inference can sometimes make predictions appear opaque and unreliable, an adequate analysis of the results can unveil valuable insights into the relationship between DL models and existing medical knowledge. Therefore, the integration of deep learning technology with the study of microscopic morphological textures in pathological imaging holds significant promise.

**Fig. 1:**
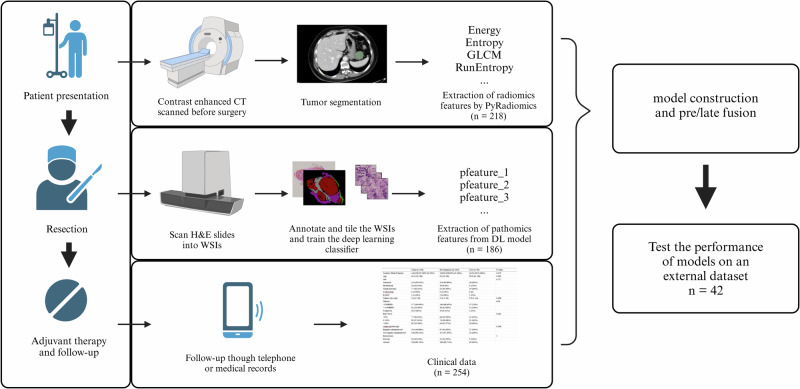
Schematic outline of this study. Multiple modalities of data were acquired during routine treatment to inform prognostic prediction. Pre-operation CE-CT scans of the abdomen were annotated for extracting radiomics features. Post-operation H&E stained slices were annotated for supervised training. Clinical data were acquired including the latest laboratory examination before surgery. Unimodal and multimodal models were fitted on the same development cohort but different subsets but evaluated on a common external validation cohort. This figure was created with BioRander.com. GLRLM: gray-level run length matrix; WSI: whole slide image; RFS: recurrence-free survival. The image was created with BioRender.com by us partially using previously created icons. Retrieved from https://app.biorender.com/biorender-templates.

As demonstrated by K.M.Boehm.etc, multi-omics models have proven effective in predicting the risk stratification of high-grade serous ovarian cancer^13^. Expanding upon this approach, a multimodal model offers a more comprehensive prediction, synthesizing from various aspects^14,15^. While radiomics has yielded promising results in GIST research, the effectiveness of applying a multi-omics model in this context remains to be verified^16,17^. In the present research, we jointly used contrast-enhanced computerized tomography (CE-CT) images and haematoxylin and eosin (H&E) -stained biopsy slides to develop and validate a multi-omics model for predicting recurrence-free survival among GIST patients. Additionally, we explored the potential biological significance of features extracted by DL model (Fig. 1).

## Results

### Cohorts and clinical characteristics

We analyzed 254 patients who were pathologically diagnosed with GIST and treated at The First Hospital of China Medical University as development cohort. Among these 254 patients, radiomics data (preoperative CE-CT) were available for 218 patients, while pathomics data (WSI) were available for 186 patients. We also collected 42 patients pathologically diagnosed with GIST in Liaoning Cancer Hospital & Institute as external validation set. Radiomics, pathomics and clinical data were full-available in this cohort. The median age at the time of diagnosis for development cohort was 61.5 years old (interquartile range (IQR): 54 ~ 66.75) and 62.5 years old (IQR: 55 ~ 68.75) for external validation set. The median tumor size (diameter) in development set was 5 cm, with an IQR of 3.5 to 7.5 cm, while the median tumor size in external validation set is 9 cm (IQR: 5.625 ~ 11.75). In development cohort, 61 patients were documented with over 5/50 HPFs in mitosis (22.45%), while the mitosis number of 19 patients (45.24%) were detected to be over 5/50HPFs. We only recommended TKI adjuvant therapy for patients with high risk of recurrence based on the modified NIH scale. However, taking into account practical commercial considerations and medical tolerance, 114 patients (44.88%) received regular adjuvant therapy in development cohort. In contrast, 39 testing patients (92.86%) received regular adjuvant therapy for a higher percentage of patients with high recurrence risk in external validation cohort (92.86% vs. 44.88%). Over a followed-up period of at least one year, 26 patients (10.24%) in development cohort and 11 patients (26.19%) in external cohort were detected to have developed recurrences, as determined through radiological examination. (Table 1).

**Table 1:** General clinical characteristics and followed-up results of cohorts

	Development (*n* = 254) (percentage/IQR)	Test (*n* = 42) (percentage/IQR)
Sex (Male/Female)	146/108 (57.48%/42.52%)	19/23 (45.24%/54.76%)
Age	61.5 (54, 66.75)	62.5 (55, 68.75)
Site		
Stomach	142 (55.91%)	29 (69.05%)
Duodenum	26 (10.24%)	0 (0)
Small intestine	77 (30.31%)	11 (26.19%)
Colorectum	6 (2.36%)	1 (2.38%)
EGIST	3 (1.18%)	1 (2.38%)
Tumor size (cm)	5 (3.5, 7.5)	9 (5.625, 11.75)
Mitosis		
<5/50HPFs	177 (69.69%)	7 (16.67%)
>=5/50HPFs	61 (22.45%)	19 (45.24%)
Unknown	20 (7.86%)	16 (38.09%)
Ki67 level		
<5%	77 (30.31%)	11 (30.95%)
5 ~ 10%	95 (37.41%)	21 (21.43%)
>10%	82 (32.28%)	18 (47.62%)
Adjuvant therapy		
Regular administered	114 (44.88%)	39 (92.86%)
Not regular administered	140 (55.12%)	3 (7.14%)
Recurrence		
Present	26 (10.24%)	11 (26.19%)
Absent	228 (89.76%)	31 (73.81%)
Radiomics data		
Available	218 (85.83%)	42 (100%)
Not available	36 (14.17%)	0 (0)
Pathomics data		
Available	186 (73.23%)	42 (100%)
Not available	68 (26.77%)	0 (0)

### Development of the radiomics model

**Fig. 2:**
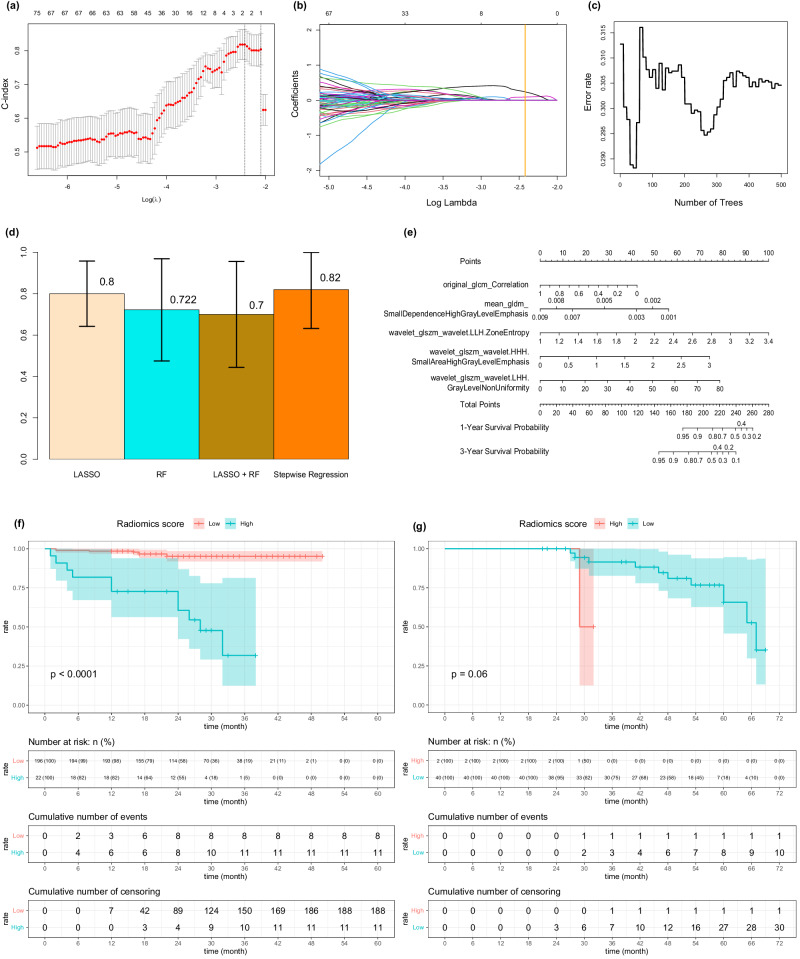
Construction of the radiomics model. **a,** **b** Selection of radiomics features related to RFS through LASSO-COX regression. **c** Selection of radiomics features related to RFS through random forest algorithm. **d** Performances of models based on different groups of features when evaluated on validation set using cross validation method (standard deviation of the mean). **e** the nomogram of radiomics model. **f** Kaplan-Meier analysis on RFS stratified by radiomics scores (Developing set, *n* = 218). **g** Kaplan-Meier analysis on RFS stratified by radiomics scores (External validation set, *n* = 42). *P*-values were calculated using the log-rank test. Glcm gray-level co-occurrence matrix, Gldm gray-level dependence matrix, Glszm gray-level size zone matrix, LASSO least absolute shrinkage and selection operator, RF random forest.

We initiated this part of analysis with data extracted from the intravenous phase images of preoperative CE-CT images, employing PyRadiomics, which provided us with 2266 features across 218 samples. For dimension reduction, we employed LASSO-COX regression with 10-fold cross-validation, which resulted in the selection of 2 features with a minimum lambda value of 0.0552 (Fig. 2a, b). Simultaneously, we applied the random forest algorithm with 10-fold cross-validation to identify prognostically relevant features from a different aspect. With a best node size of 45 and 500 decision trees, this method led to the selection of 12 features through a variants-hunting method (Fig. 2c). Then, we combined these features and further screened them through step-wise COX regression. This process resulted in the generation of four groups of features (features selected by LASSO-COX regression, features selected by random forest algorithm, combined features and features re-selected by step-wise COX regression) were generated. Utilizing a multivariate COX regression model with 10-fold cross-validation, we constructed and validated predictive models for each of these four groups of features. Notably, the model constructed using the features re-selected through step-wise COX regression, specifically “original_glcm_Correlation”, “mean_gldm_SmallDependenceHighGrayLevelEmphasis (SDHGLE)”, “wavelet_glszm_wavelet.LLH.ZoneEntropy (ZE)”, “wavelet_glszm_wavelet.HHH.SmallAreaHighGrayLevelEmphasis (SAHGLE)”, and “wavelet_glszm_wavelet.LHH.GrayLevelNonUniformity (GLNU)” performed the best on validation set within the entire development set during the cross validation process (Mean C-index: 0.82, standard deviation: 0.188) (Fig. 2d, e). We further assessed the model’s ability to separate prognostic prediction using Kaplan-Meier method. The radiomics model demonstrated good separating capacity in the development cohort (*p*-value < 0.0001) (Fig. 2f). However, it didn’t reach statistically significance in the external cohort (*p* = 0.06) (Fig. 2g). Generally, patients with lower radiomics predictive scores were expected to achieve longer recurrence-free survival periods. The score of radiomics model could be calculated by this formula: radscore = (42–42*original_glcm_Correlation)+(63–7000*mean_gldm_SmallDependenceHighGrayLevelEmphasis)+(41.667*wavelet_glszm_wavelet.LLH.ZoneEntropy-41.667) + (24.708*wavelet_glszm_wavelet.HHH.SmallAreaHighGrayLevelEmphasis)+(0.984*wavelet_glszm_wavelet.LHH.GrayLevelNonUniformity).

### Development and explanation of the pathomics model

We trained a tissue classifier based on the ResNet50 network structure, and its performance in recognizing tumor tissue type was notably high, as indicated by the confusion matrix reported for validation set (Accuracy: 0.96) (Supplementary Figure 1). Considering that tumor tissue is the predominant tissue type in GIST, we decided to focus on features extracted from the tumor tissue region.

**Fig. 3:**
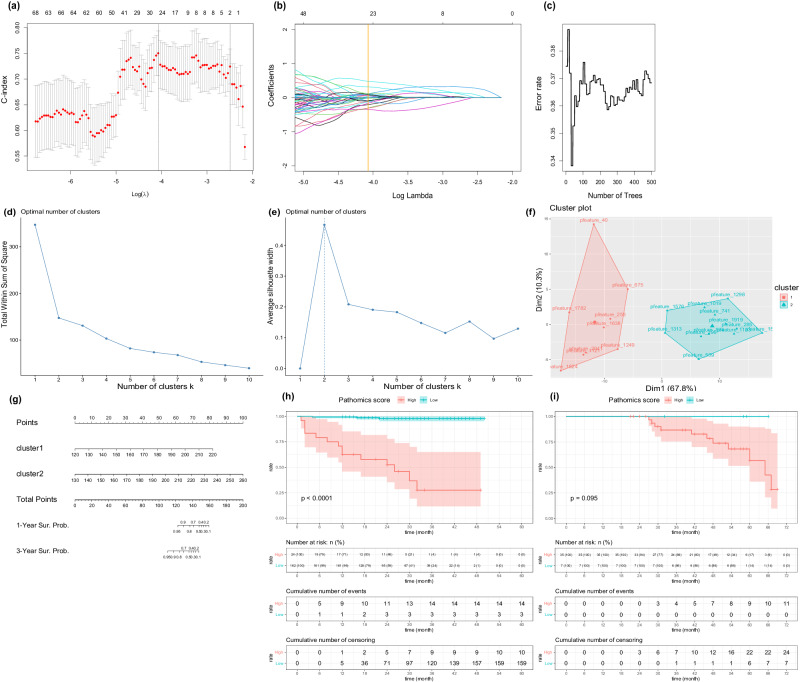
Construction of the pathomics model. **a**, **b** Selection of radiomics features related to RFS through LASSO-COX regression. **c** Selection of radiomics features related to RFS through random forest algorithm. **d** Determine the best cluster number by total within sum of square. **e** Determine the best cluster number by average silhouette width. **f** Visualization of the result of cluster. **g** Nomogram of pathomics model. **h** Kaplan-Meier analysis on RFS stratified by pathomics scores (Developing set, *n* = 186). **i** Kaplan-Meier analysis on RFS stratified by pathomics scores (External validation set, *n* = 42). *P*-values were calculated using the log-rank test.

After feature screening, a total of 21 pathomics features were selected using stepwise COX regression, which were excess in constructing a multivariate COX regression model within a development set of 186 samples (Fig. 3a–c). We then determined the optimal number of clusters and divided the 21 pathomics features into 2 clusters (Fig. 3d–f). Initially, two sub-models were constructed using these two clusters of pathomics features, and predictive scores were calculated based on these sub-models (calculation functions were supplied in Supplementary Table 1). Subsequently, pathomics models were constructed using the pathomics features derived from the sub-models through a multivariate COX regression model with 10-fold cross-validation (Fig. 3g). The discriminative ability of the model was validatied and found to be significant in development set (*p*-value in development set < 0.0001, *p*-value in external validation cohort = 0.095) (Fig. 3h, i). The score of pathomics model could be calculated by this formula: patscore = (0.82*cluster1 score-98.353) + (0.769*cluster2 score-100).

**Fig. 4:**
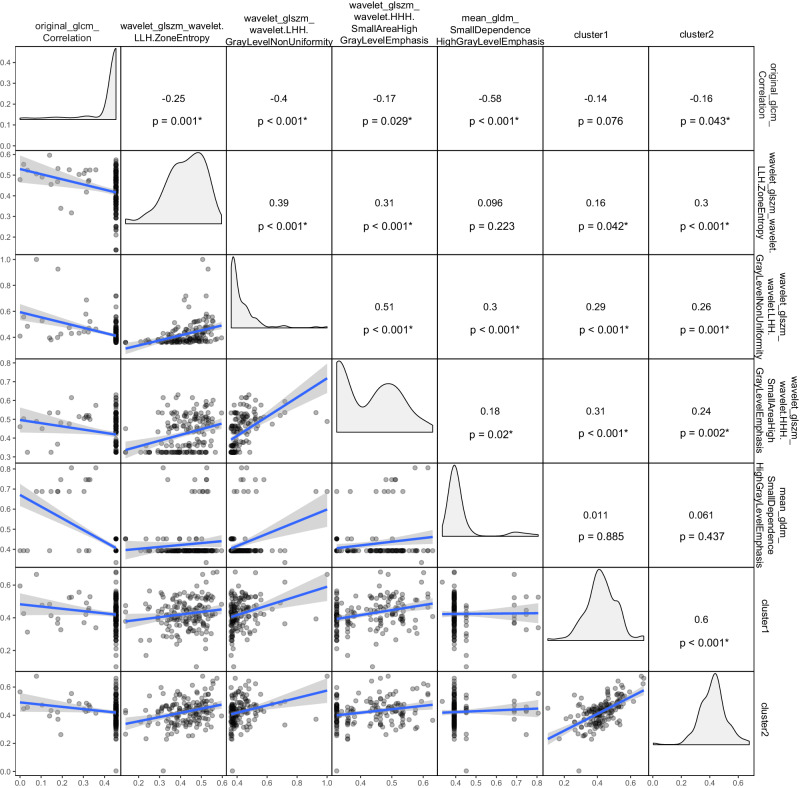
Correlation map. The correlation map between pathomics (cluster1, cluster2) and radiomics (original_glcm_Correlation, mean_gldm_SmallDependenceHighGrayLevelEmphasis, wavelet_glszm_wavelet.LLH.ZoneEntropy,wavelet_glszm_wavelet.HHH.SmallAreaHighGrayLevelEmphasis, wavelet_glszm_wavelet.LHH.GrayLevelNonUniformity) features.

**Fig. 5:**
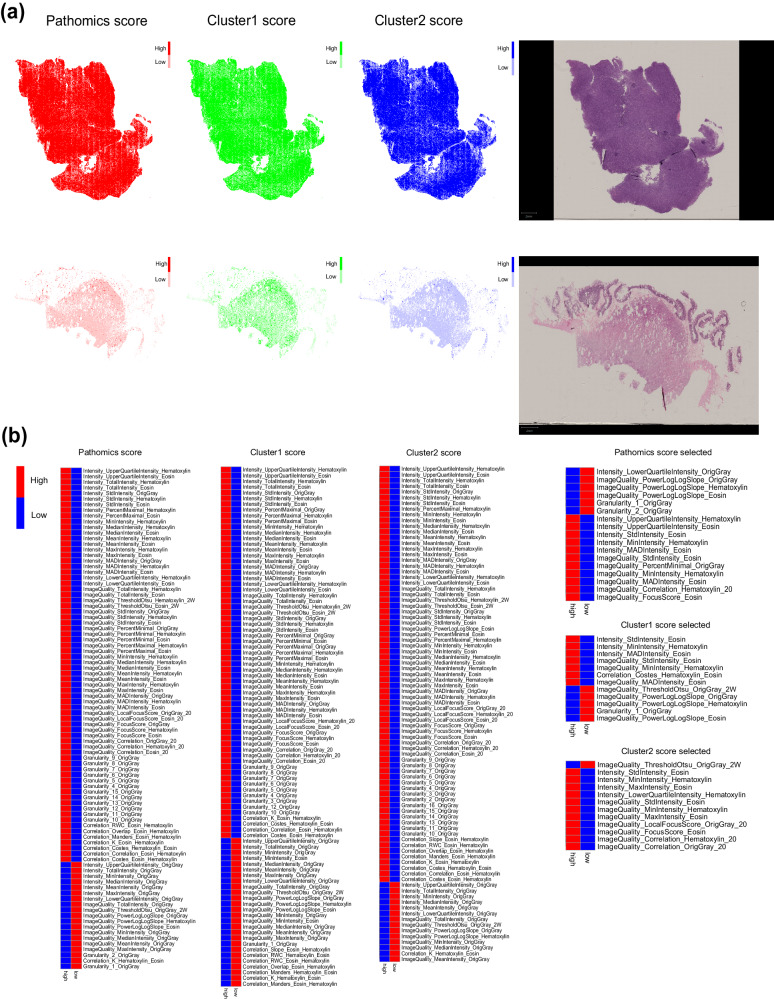
The explanation of pathomics features. **a** The WSIs and distribution of cluster1 scores, cluster2 scores, and pathomics scores with the highest the lowest pathomics score. **b** Heatmaps of the texture feature distribution extracted by CellProfilier stratified by high and low scores of features. Pathomics score represents the features distributed differently between patches with high pathomics scores in WSIs with high pathomics scores and patches with low pathomics scores in WSIs with low pathomics scores. Cluster1 score represents the features distributed differently between patches with high cluster1 scores in WSIs with high cluster1 scores and patches with low cluster1 scores in WSIs with low cluster1 scores. Cluster2 score represents the features distributed differently between patches with high cluster2 scores in WSIs with high cluster2 scores and patches with low cluster2 scores in WSIs with low cluster2 scores. Pathomics score selected, cluster1 score selected, and cluster2 score selected represent the features selected through two stages.

To explore potential morphological relevance between radiomics and pathomics images, we utilized a correlation heat map (Fig. 4). This analysis revealed positive correlations between cluster1 and cluster2 scores with three radiomics features (ZE, SAHGLE, GLNU), indicating that higher values represented greater heterogeneity in texture pattern and intensity (Supplementary Table 2). Additionally, cluster2 was negatively correlated with ‘original_glcm_Correlation’. In Fig. 5a, we presented two WSI images with the highest and lowest pathomics scores, respectively, alongside visualizations of the predictions made by the deep learning classifier and and the distributions of cluster1 scores, cluster2 scores, and pathomics scores. Following this, we selected 6800 representative patches from 34 WSIs and extracted texture features using CellProfiler. In the quantitative analysis, we calculated cutoff values to divide cluster1 scores, cluster2 scores, and pathomics scores. As a result, we identified 17 texture features that were differently distributed in patches with high and low pathomics scores. Additionally, 12 texture features differently distributed were detected for cluster1 and cluster2 scores, respectively (Fig. 5b). All visualized predictions and distributions of pathomics features are displayed in Supplementary Fig. 2.

### Development of the clinical model

**Fig. 6:**
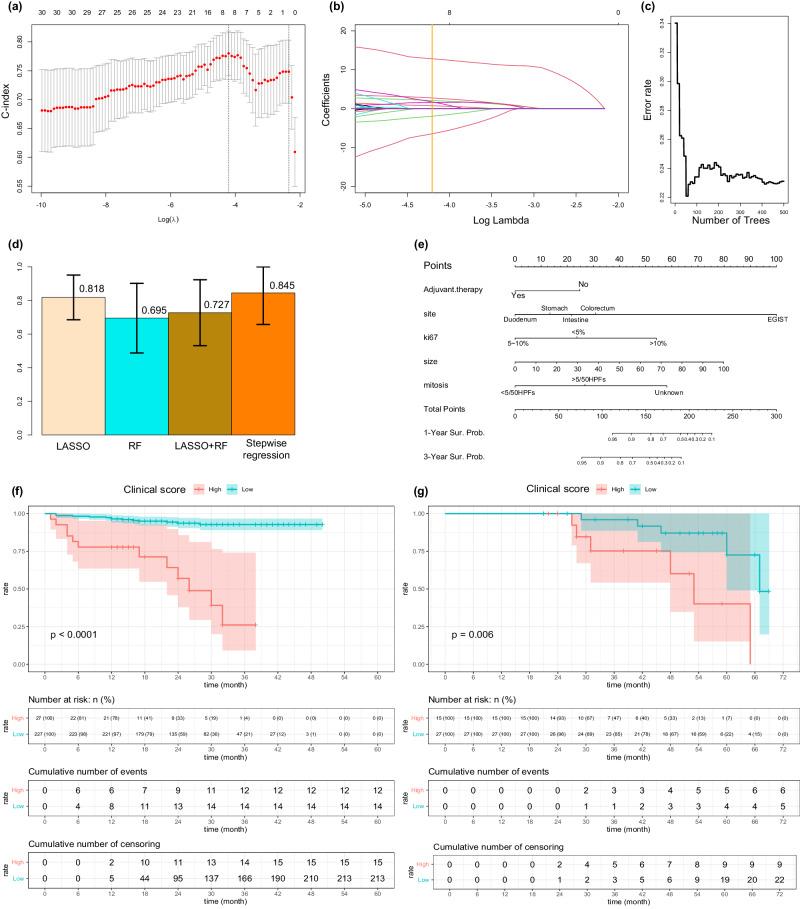
Construction of the clinical model. **a**, **b** Selection of clinical features related to RFS through LASSO-COX regression. **c** Selection of clinical features related to RFS through random forest algorithm. **d** Performances of models based on different groups of features when evaluated on validation set using cross validation method (standard deviation of the mean). **e** the nomogram of clinical model. **f** Kaplan-Meier analysis on RFS stratified by clinical scores (Developing set, *n* = 254). **g** Kaplan-Meier analysis on RFS stratified by clinical scores (External validation cohort, *n* = 42); *P*-values were calculated using the log-rank test.

We complied a total of 30 features, which encompassed sex, age, original site, ki67 positive level, height, weight, BMI index, GI bleeding, obstruction, intra-abdominal bleeding, surgery method, tumor size, mitosis number, regular adjuvant therapy application, and regular preoperative biochemical detection results. Utilizing LASSO-COX regression with 10-fold cross validation, we identified 8 significant predictors. Four features were selected by random forest algorithm (Fig. 6a–c). Then we combined the features and re-selected them using stepwise COX regression through the same method applied for radiomics feature selection. Eventually, adjuvant therapy administration, tumor site, tumor size, mitosis number and ki67 positive level were selected to construct clinical model utilizing within the development cohort (*n* = 254). The distinguish capacity was verified and examined to be significant in both development cohort and external validation cohort (p value in development cohort: < 0.0001, *p*-value in external validation cohort: 0.006) (Fig. 6f, g). The predictive score given by clinical model could be calculated by this formula: clinscore = (4*tumor size)+[(25 for didn’t receive regular adjuvant therapy) or (0 for received regular adjuvant therapy)]+[(0 for duodenum) or (13 for stomach) or (22 for small intestine) or (31 for colorectum) or (100 for EGIST)] + [(0 for ki67 positive level between 5% to 10%) or (24 for ki67 positive level < 5%) or (54 for ki67 positive level > 10%)] + [(0 for mitosis number < 5/50HPFs) or (27 for mitosis number > 5/50HPFs) or (58 for mitosis number unknown)].

### Constructing multi-omic models and model validation

**Fig. 7:**
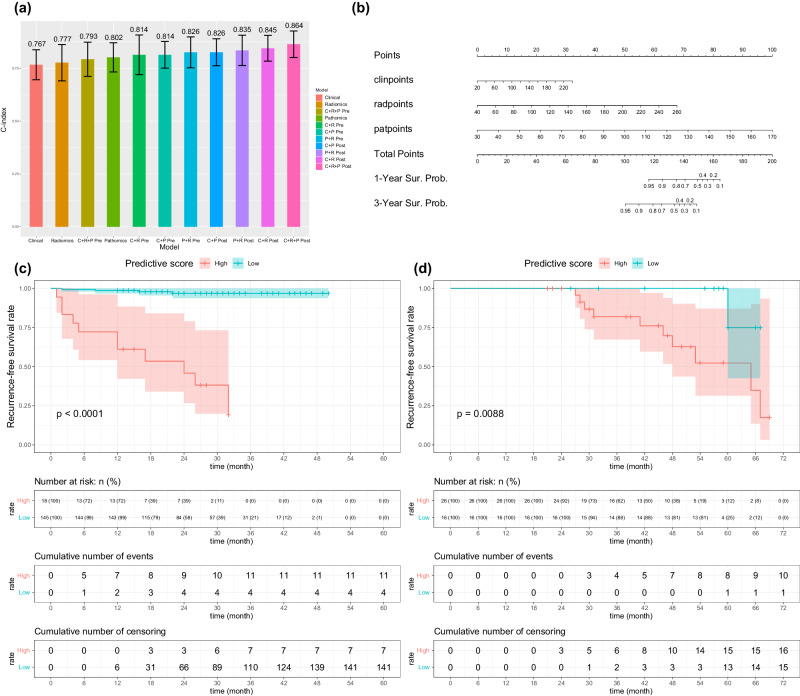
Construction of the multimodal model. **a** Performances of all models on external validation cohort (standard deviation of the mean). **b** Nomogram of the multimodal model. **c** Kaplan-Meier analysis on RFS stratified by fusion scores (Developing set, *n* = 163). **d** Kaplan-Meier analysis on RFS stratified by fusion scores (External validation cohort, *n* = 42). *P*-values were calculated using the log-rank test. R radiomics model, *P* pathomics model, *C* clinical model.

We developed multi-omics models by integrating uni-omics models through both early fusion and late fusion methods. In total, we constructed 11 models, including three uni-omics models. To evaluate their effectiveness, we subjected all models to rigorous testing on a shared external validation cohort, utilizing the bootstrap method with 500 iterations. The models were compared based on their C-index performance metrics (Fig. 7a). Remarkably, the combined model that integrated radiomics, pathomics, and clinical data though post-fusion demonstrated the strongest performance, with an average C-index of 0.864 and a standard deviation of 0.063 (Fig. 7a, b). The multimodel model reached statistical significance on distinguishing the prognosis within both the development cohort and external validation cohort using K-M method (*p*-value of log-rank test: < 0.0001 for development cohort and 0.0088 for external validation cohort) (Fig. 7c, d). The predictive score of multimodal model could be calculated by this formula: Predictive score = (0.408*radscore-16.306) + (0.714*patscore-21.429) + (0.0085*clinscore-2.069). The forest plots of unimodal models and the multimodal model were exhibited in Supplementary Figure 3. The DCA curves was exhibited in Supplementary Figure 4.

### Genomic mutation

Out of the total cohort, targeted gene mutation detection was conducted for 36 patients to guide medicine administration. Among these patients, all were detected to possess at least one mutation in either the KIT or PDGFRA genes, with the exception of one individual, a 15-year-old boy, who had an SDHA mutation. Additionally, sporadic mutation in FGFR1, NF1, NTRK1, PIK3CA and SDHB were detected in a few cases. Notably, nine patients exhibited mutations at multiple positions. In our survival analysis, we observed that patients with mutations at multiple positions tended to have a poorer prognosis (log-rank test *p*-value: 0.044) (Supplementary Fig. 5). We noticed that the scores given by multi-omics model were of a same level in patients with KIT11 exon mutation or not. However, patients with multiple mutation positions tended to have higher scores compared with others, though the difference was not significant in statistics (*p* = 0.14, Supplementary Fig. 6).

## Discussion

In the radiomics uni-modal model, we observed that three features were identified as hazard factors in predicting RFS: ZE, SAHGLE, and GLNU. ZE measures the uncertainty or randomness in the distribution of zone sizes and gray levels within the image. A higher ZE value indicates greater heterogeneity in texture patterns. Similarly, GLNU measures the variability of gray-level intensity values in the image, with a lower value suggesting more homogeneity in intensity values. SAHGLE measures the proportion of smaller size zones with higher gray-level values in the image. These findings suggest that a more heterogeneous and complex distribution of textures within the tumor lesion is associated with a poorer prognosis. Additionally, SDHGLE, which measures the joint distribution of small dependence with higher gray-level values, was identified as a protective factor. A high SDHGLE value may indicate more interconnected areas of high gray-level values, possibly representing more homogeneous solid components within the mass.

In the development of the pathomics model, original pathomics features were extracted from the first fully connected layer as a vector^18^. This vector-based representation makes it challenging to precisely interpret the predictive significance of individual features. In that case, we believed it was reasonable to group pathomics features into clusters during the following process. Deep learning networks have demonstrated remarkable effectiveness in solving non-linear problems; however, the mechanism underlying their inferences have remained somewhat mysterious^19,20^. Various research endeavors have sought to demystify the inference process of deep learning networks and transform it into a more interpretable pathway. For instance, Kitamura et al. conducted research based on local interpretable model-agnostic explanations (LIME), while Elmarakeby et al. achieved this by constructing a sparse network structure and re-definite the learning patterns^21,22^. To date, there isn’t a universally accepted approach for achieving explainable artificial intelligence (XAI)^23^. The method for creating XAI solution appear flexible but come with varying limitation, making widespread recognition challenging. In our present research, we chose a simple and intuitive method to explore the significance of deep learning features. This involved visualization and a quantitative comparison of pathomics features extracted by CellProfiler.

When examining the heat maps visualizing the values of the clusters and pathomics scores, an intriguing observation emerged—the distribution of values within patches sometimes exhibited a regular fluctuation pattern in square block areas. This phenomenon can be attributed to the underlying principles of CNN, which extract features through convolutions. The stack of convolution layers could result in square ‘sensory areas’ that provide the visual context of the CNN model. Notably, the border regions of these square ‘sensory areas’ appear to carry a higher weight of sensitivity. In the context of our study, higher pathomics scores were associated with poorer prognosis. In the first stage of quantitative analysis, we detected 89 texture features differently distributed between patches with high pathomics scores in WSIs with high pathomics scores and those with low pathomics scores in WSIs with low pathomics scores, 91 textures features differently distributed between patches with high cluster1 scores in WSIs with high cluster1 scores and those with low cluster1 scores in WSIs with low cluster1 scores, and 87 texture features differently distributed between patches with high cluster2 scores in WSIs with high cluster2 scores and those with low cluster2 scores in WSIs with low cluster2 scores. Furthermore, in the second stage, we detected texture features differently distributed between patches with high and low pathomics scores, cluster1 scores, and cluster2 scores in WSIs at varying level. Then we selected texture features that were commonly detected in both stages to represent features that differed from pathomics scores, cluster1 or cluster2 scores. The results of the second stage were presented in Supplementary Figure 7. According to the feature dictionary compiled from CellProfiler project, we observed that tumor area with higher pathomics scores exhibited higher image intensity and sharper visual characteristics. Increased intensity in both the hematoxylin and eosin channels suggested hyperstaining and sharper vision, possibly indicating a higher density of distributed cells. Similarly, elevated levels of cluster1 and cluster2 scores were associated with higher image intensity. Notably, a high level of cluster2 scores was correlated with high value of ‘ImageQuality_Correlation_Hematoxylin_20’ and ‘ImageQuality_Correlation_OrigGray’, indicative of more blurred views in most cases.

The clinical model was developed based on five key factors: administration of adjuvant therapy, tumor size, tumor site, mitosis number, and Ki67 expression. Among these factors, tumor site, tumor size, and mitosis number are well-established as significantly associated with prognosis. Both the widely recognized modified NIH scale and the AFIP scale incorporate these variables into their assessment criteria for predicting recurrence risk. In the case of GIST, adjuvant therapy primarily involves TKIs such as imatinib. Imatinib has been proven to be highly effective in preventing recurrence, particularly in patients at high risk of recurrence. A 10-year follow-up study of a clinical trial demonstrated that three years of adjuvant therapy is superior to one year of administration alone in reducing recurrence and mortality rates^24^. On the other hand, ki67 has been validated as a valuable biological marker for assessing the malignant risk of GIST^25–27^. Elevated ki67 positive levels typically indicate higher proliferative activity, which is directly correlated with an increased malignant potential^28^.

In our present study, we constructed three uni-modality models based on radiomics, pathomics, and clinical information. Radiomics model was the uni-modality model with the best performance and it came up with inference from pre-operative sight, while pathomics model and clinical model were mainly based on post-operative information. We validated the performance of the multi-omics model, formed by merging all three uni-modality models, as the most effective in predicting recurrence-free survival in the external validation set (Average C-index: 0.864). In general, multi-omics models draw upon information from multiple sources, offering a more comprehensive basis for predictions, thereby enhancing accuracy and reliability. The efficacy of constructing multi-omics models has been demonstrated in various fields, such as colorectal cancer lung metastasis and ovarian malignant tumor^29^. Specifically, our approach combined radiomics and pathomics technology, delving deeply into the prognostic potential of detailed texture features. Through the ‘computer vision (CV)’ perspective, we introduced a novel and instructive means of recognizing imperceptible features for humanity within radiological and pathological images.

This study has several limitations that should be acknowledged. Firstly, while we explored the significance of deep learning features, the specific relationships with biological knowledge and the underlying mechanism still remained mysterious. We anticipate that with the integration of single-cell RNA sequence technology and emerging spatial transcriptome sequencing techniques, a clearer connection between CV features and biological processes will be unveiled. Secondly, patients with low-risk GISTs were not tended to receive regular radiological examination, which could bring selection bias. Thirdly, the training sets for different models were of varying amount of data, however, the performance on training sets was only used for the uni-omics feature selection. When compared different models, we used the performance on a common test set. However, sample size of training set might do effect the model performance. Generally, a bigger data set might lead to a better predictive model. In that case, the performance of radiomics model (with the biggest sample volume) could be overestimated and the performance of multi-omics model (with the smallest sample volume) could be underestimated. Fourthly, in the deep learning model training process, tumor and background image type took the predominant ratio, which could be a potential explanation to the limited predictive accuracy of other tissues in validation set.

We constructed a multi-omics model and validated its priority than uni-modality models. To deal with the inference process of deep learning model which is hard to understand, we explored the biological significance and correlations of radiomics and pathomics features. The multimodal model could supply a more comprehensive and stable prediction on RFS of patients with GIST, which could guide the clinical decision-making in the precise application of TKI adjuvant therapy. Besides, as we applied deep learning algorithm (convolutional neural network, CNN) in pathomics feature extraction, the “black box” in the process of inference could make the prediction of pathomics model confusing. As a result, we made effort in the interpretation of pathomics features. Through visualization and quantitative analysis, we displayed the distribution of pathomics features and the relationship between pathomics features and the visualized texture features. Combined with multiple-immunohistological-stained pathological materials and sequencing technology, deep learning could inspire a new sight in further studies in microenvironments by appropriate process.

## Methods

### Patients enrollment and cohort curation

A total of 354 patients underwent surgery and pathologically diagnosed with GIST in The First Hospital of China Medical University from 2019 to 2022 were retrospectively enrolled. Following the inclusion and exclusion criteria below, there were 254 patients eligible in this study eventually. Inclusion criteria were as follows: (1) Patients who underwent surgical resection with the intent of curative treatment for GIST; (2) Diagnosis confirmed through postoperative pathology and immunohistochemistry examinations; (3) Availability of at least one year of follow-up data. Exclusion criteria encompassed: (1) Patients who received neoadjuvant therapy prior to surgery; (2) Small GISTs with a largest diameter less than 1 cm; (3) Cases with macroscopic residuals of the primary lesion; (4) Patients with concurrent malignancies; (5) Preoperative venous phase CE-CT images available but not meeting quality standards; (6) H&E stained whole slide images (WSI) available but not meeting quality standards.

Flow chart of the selection process was presented in (Supplementary Figure 8). Patients were scheduled for followed-up appointments every three months, during which their survival time and survival status at the last follow-up were meticulous recorded. RFS was defined as the time elapsed from surgical resection to the verified occurrence of recurrence at any site, as confirmed by CE-CT scans. Patients deemed to be at high risk of recurrence were recommended to undergo targeted gene mutation detection to enable precise application of adjuvant therapy. However, due to commercial constraints, only 36 patients actually underwent targeted gene mutation detection. Preoperative radiological images were accessible for 218 patients, while histopathological H&E digital slides were available for 186 patients. Among these, 163 patients possessed data from all three modalities: preoperative CE-CT image, WSI of histology slices, and clinical characteristics information. In addition, we retrospectively gathered 42 patient records from Liaoning Cancer Hospital & Institute, covering the period between 2018 and 2022. These patients underwent surgery and received pathological diagnosis of GIST. The data collected included comprehensive information, making it an ideal external validation cohort for validating the performance of our models. (Supplementary Figures 8 and 9 and Table 1). The external validation cohort was never used for feature selection.

This study was conducted in accordance with the Declaration of Helsinki and has received approval from the Institutional Ethics Review Board of The First Hospital of China Medical University and the Institutional Ethics Review Board of Liaoning Cancer Hospital & Institute. As this was a retrospective study, the requirement for written signed informed consent was waived. This study was compliant with the ‘Guidance of the Ministry of Science and Technology (MOST) for the Review and Approval of Human Genetic Resources’.

### Radiological image acquisition and ROI annotation

CT images were acquired using 64-detector CT scanners (Supplementary Table 3). The dynamic contrast-enhanced scan was performed, with 90–150 ml of 320–370 mgI/mL nonionic iodine contrast injected intravenously at a rate of 2.0–3.0 ml/s. Portal venous phase (PVP) imaging was obtained at 65–80 s after the intravenous injection of the contrast agent. The region of interest (ROI) of tumor on PVP imaging was delineated with the whole data in a blind fashion by one of two radiologists (D.L.F. and S.L.) who possess more than 2 years of experience in abdominal imaging. The segmentations were validated and modified by one of the two radiologists with 15 (reader 1, L.J.) and 10 years (reader 2, M.Q., a co-author of the present study) of experience in the interpretation of abdominal CT. The segmentation of tumors was conducted using 3D slicer software (Ver. 5.1.0).

### The extraction of radiomics features

The initial step involved standardizing the images using the ROI mask and resampling them to a solution of 2 * 2 * 2 mm. Utilizing PyRadiomics (a package based on Python, widely used for radiomics feature extraction, ver. 3.1.0), we extracted a total of 2266 radiomics features. These features included descriptors related to shape, size, gray-level co-occurrence matrix, gray-level size zone matrix, gray-level run length matrix, gray-level dependence matrix, neighbouring gray tone difference matrix, and wavelet features in both 2.5D and 3D.

### Construction of radiomics model

We employed a comprehensive approach to identify potential radiomics features associated with RFS from a pool of 2266 radiomics features. Initially, we used LASSO-COX regression and the random forest algorithm with a 10-fold cross validation setup to screen for relevant features^30,31^. Subsequently, we combined the features respectively selected by LASSO-COX regression and random forest algorithm and removed the duplicated features. Next, stepwise COX regression which is a statistical technique used to identify a subset of predictor variables essential for constructing predictive models was adopted to re-selected a optimized subgroup of features^32^. Following initial screening, we categorized the features into four groups: (1) features selected by LASSO-COX regression; (2) features selected by random forest algorithm; (3) a combination of features selected by LASSO-COX or random forest method; (4) features re-selected from the combined feature group using stepwise COX regression. To determine the most effective feature group, we selected the group with the highest C-index on validation sets within a 10-folds cross-validation of the entire development set (*n* = 218) repeated for 200 times. Finally, with these selected features, we constructed the radiomics model using a multivariate COX regression approach.

### WSI preparation and tissue annotation

To collect H&E digital slides, we reviewed the electronic health record to find suitable pathology cases and expert pathologists (X.H., a co-author and G.Y.J., a co-corresponding author) reviewed the slides to select qualified specimens for digitization which contain enough tumor for further evaluation. After digital scanning, two expert pathologists (X.H. and G.Y.J.) partially annotated 30 H&E WSIs using the QuPath^33^. The approach was to label example regions of tumor, stroma, mucosa, smooth muscle, and necrosis with reasonable but imperfect accuracy. We then exported these annotations as bitmaps and converted them to GeoJSON objects.

### Tissue classifier training and validation

We tiled the 30 WSI images, each annotated to correspond with an annotation map, into patches measuring 224*224 pixels. Each patch was tagged with the tissue type that occupied the largest proportion of the corresponding area on the matched annotation map. As we generated an extensive data set exceeding 6,000,000 patches, significantly larger than the original ImageNet data set, training on the entire data set would have been prohibitively expensive. Consequently, we decided to randomly select 300,000 patches (Background: 178,353/59.45%; Tumor: 108370/36.12%; Mucosa: 3339/1.11%; Muscle: 4034/1.34%; Stroma: 3736/1.25%; Necrosis: 2168/0.72%), which were than divided into a training set and a validation set in a 7:3 ratio (Training set [Background: 124366/5922%; Tumor: 76601/36.48%; Mucosa: 2201/1.05%; Muscle: 2764/1.32%; Stroma: 2497/1.19%; Necrosis: 1571/0.75%], validation set [Background: 53987/59.99%; Tumor: 31769/35.3%; Mucosa: 1138/1.26%; Muscle: 1270/1.41%; Stroma: 1239/1.38%; Necrosis: 597/0.66%]). In the present study, we didn’t use overlapping patches. The tiles at the border which was not square were excluded as most of these patches were blank and would brought unnecessary steps in the training stage, for instance, image stretching or cropping. We applied randomly rotation and flip to conduct data amplification of training set. Subsequently, we trained a ResNet50 model for 30 epochs, utilizing a learning rate of 0.0001, weights pretrained on ImageNet and Adam Optimizer^34^. Our objective function was class-balanced cross-entropy, and we used small batches of 64 patches to train on a single NVIDIA GTX3090 GPU. The model underwent validation on the validation set after each epoch, and the results and parameters of the best-performing epoch was saved. The confusion matrix of the results of the best-performing epoch was shown in Supplementary Figure 1. Python (Ver. 3.9.1) was used for model training.

### Pathomics features extraction and model construction

We tiled all the 186 WSIs into patches of the same size as those utilized during training and retained their respective WSI association. Subsequently, we fed all these patches into the trained classifier in batches. After passing through 5 ResNet blocks, the output took on a shape of 2048 * 7 * 7. To convert this output into a vector of length 2048, we applied a global average pooling layer. We read out the vector on this layer as pathomics texture features. A scalar on each position of the vector represented a feature. We piled features from all patches cropped from a common WSI by the second dimension to create constructed data. We calculated the mean values of all the patches to produce WSI-level feature data. Next, we began by initially screening pathomics features using LASSO-COX regression and the random forest algorithm. Subsequently, we refined our selection by employing stepwise regression, similar to our approach in selecting radiomics features. Then we utilized the ‘fviz_nbclust’ function from the ‘factoextra’ package to determine the optimal number of clusters for our selected features. This function utilizes metrics such as total within-cluster sum of squares and average silhouette and gap statistics. Following the determination of the optimal cluster number, we performed feature clustering and visually presented the cluster results. Based on these outcomes, we developed pathomics sub-models for RFS using a multivariate COX regression approach, incorporating features from each cluster. Predictive scores were then calculated for each cluster’s pathomics features. Finally, we constructed the pathomics model using multivariate COX regression in our development cohort (*n* = 186).

### Explore the biological significance of pathomics features

In our initial exploration, we investigated the correlations between pathomics and radiomics features by constructing a co-distribution matrix based on Pearson’s correlation coefficients. Radiomics features, crafted through feature engineering, inherently possess greater interpretability. We considered p-value less than 0.05 as statistically significant in these analysis. Following this, to provide a clearer insight into pathomics clusters, we identified WSIs with the highest and lowest scores for cluster1, cluster2, and the pathomics scores derived from the pathomics model, resulting in a total of 34 WSIs. Subsequently, we visualized the distributions of these two clusters and pathomics scores at the WSI-level. To further distinguish the differences between areas with high and low scores, we calculated cutoff values to stratify cluster1 scores, cluster2 scores, and pathomics scores into high and low score group. Then we selected 6800 representative patches in the 34 WSI images and extracted texture features by CellProfilier (ver. 4.2.1). Following methods demonstrated in D.Chens’ research, we processed patches with “UnmixColors”, “ColorToGray”, “MeasureImageQuality”, “MeasureImageIntensity, “MeasureColocalization” and “MeasureGranularity” modules^35^. Then we analyzed the distributions of features and their correlations with labels using Mann-Whitney U test. Texture features with significantly different distributions between high and low score groups, with a two-sided p-value less than 0.05, were selected. Heat maps were employed to visualize these differences. All the values were centered by means and scaled based on Z-scores. We then summarized and elucidated the significance of pathomics features, combining them with an explanation to the texture features provided by CellProfilier.

### Construction of clinical model

To identify robust biomarkers strongly associated with RFS, we employed a sequential approach which combined LASSO-COX regression, random forest algorithm, and stepwise regression. Similar as the construction of radiomics model, the 4 groups of features (features selected by LASSO-COX regression, features selected by random forest algorithm, combined of features selected by LASSO-COX regression and random forest algorithm, and features re-selected by stepwise COX regression) were validated by 10-fold cross validation in the entire development set (*n* = 254). The group with highest C-index on the validation set in cross validation were used to construct clinical model. This clinical model was ultimately built using a multivariate COX regression approach.

### Construction of Multi-omics models and efficiency validation

We developed multi-omics models through both early fusion and late fusion workflows. In the early fusion approach, we directly combined the features from different uni-omics models and constructed multi-omics model on merged data set. In contrast, in the late fusion workflow, we combined the predictive scores generated by uni-omics models as additional features for the construction of multi-omics models, all on the same merged data set used in early fusion workflow. Following this integration, we ended up with a total of 11 models, including the clinical model (C/Clin), radiomics model (R/Rad), pathomics model (P/Pat), integrated models of clinic and radiomics merged through early fusion or late fusion (R + C pre/ R + C post), integrated models of clinic and pathomics merged through early fusion or late fusion (P + C pre/ P + C post), integrated models of radiomics and pathomics merged through early fusion or late fusion (R + P pre/ R + P post) and integrated models of all three modalities of data merged through early fusion or late fusion (R + P + C pre/ R + P + C post). Subsequently, we validated all 11 models on a common external validation cohort using a bootstrapping method, repeating it 500 times. The mean C-index with standard deviation was selected as the measurement of effectiveness. We compared and gave a rank of the effectiveness on the external validation cohort of all models. Additionally, we defined the cut-off points for the predictive scores of each model and categorized patients into “high score” or “low score” groups. We evaluated the models’ ability to distinguish prognosis through Kaplan-Meier survival curve analysis with log-rank tests.

### Gene mutation status detection

Tissue specimens from patients decided to undergo targeted gene mutation detection were provided to Simcere Diagnostics®. Subsequent steps involved next generation sequencing (NGS) and the targeted identification of gene mutation sites, including KIT, PDGFRA, FGFR1, NF1, NTRK1, NTRK2, NTRK3, PIK3CA, SDHA, SDHB, BRAF, KRAS, and NRAS, all conducted by Simcere Diagnostics®. The targeted sequenced genes were in a panel device supplied by Simcere Diagnostics® aimed to predict the sensitivity to targeted drugs approved by FDA, recommended by guidance, and in clinical trials. Since the detection primarily served as a guide for the application of adjuvant therapy, only specific gene sites of relevance were examined. The results were then synthesized into a mutation landscape (Supplementary Fig. 5a). To assess the impact of different mutation types on prognosis, we conducted Kaplan-Meier survival curve analysis with log-rank testing. A two-side p-value of less than 0.05 was considered statistically significant (Supplementary Fig. 5b, Supplementary Fig. 5c). We used U-test to analyze the relationship between the phenotype of gene mutation and predictions of multi-omics model (Supplementary Fig. 6).

### Statistical analysis

In this study, we conducted comparative analyses of continuous variables using the t-test when they followed a normal distribution, or the Mann-Whitney U test if not. Categorical features were compared using either the chi-square test or the Fisher exact test, depending on the sample volume as judged appropriate. The normality of data was examined by Shapiro test. Survival curves were generated using Kaplan-Meier method and compared using the log-rank test. Cox regression was used to conduct predictive models and hazard ratios (HR) were calculated. The PH assumption of Cox regression was validated. A global *p*-value < 0.05 was recognized as violation^36^. Pearson’s correlation coefficient was applied in exploring the relationship between features. All the cut-off points in the present study were determined utilizing “surv_cutpoint” function in “survminer” package (ver. 0.4.9) based on maximally selected rank statistics to providing a value of cutpoint that corresponding to the most significant relation with outcome. Maximally selected rank statistics assumes that an unknown cutoff in independent variables determines two groups of observations regarding the response, and the two groups have the biggest statistical divergences between each other. This statistics is an appropriate standardized two-sample linear rank statistic of the responses that represents the difference between two groups. Additionally, all the cut-off points were defined on the development cohort and tested on the external validation cohort. All statistical analyses were performed using R software (Ver. 4.2.1) or Python (Ver. 3.9.1). The LASSO-COX regression method and random forest algorithm were performed using “glmnet” (ver. 4.1–7), “survival” (ver. 3.5–7) and “randomForestSRC” (ver. 3.2.2) packages. The COX regression and PH assumption validation were accomplished using “survival” (ver.3.5–7) and “survminer” (ver. 0.4.9) packages. The nomograms were developed with “rms” (ver. 6.6–0) package. Visualization related packages included “ggplot2” (ver. 3.4.2), “wesanderson” (ver. 0.3.6), “VennDiagram” (ver. 1.7.3), “ComplexHeatmap” (ver. 2.14.0), “circlize” (ver. 0.4.15), “corrmorant” (ver. 0.0.0.9007) and “pheatmap” (ver. 1.0.12). Generally, a *p*-value < 0.05 was considered to indicate statistical significance. Decision curve analysis (DCA) was conducted by “ggDCA” (ver. 1.2) package.

## Supplementary information

Supplementary Files

## Data Availability

The CE-CT images, H&E images, and clinical information analyzed during the current study are not publicly available for patient privacy proposes. Data analyzed in this study are available from H.S. (hsong@cmu.edu.cn) through a reasonable request. Access to the data will be restricted to non-commercial research which removes patient-sensitive information.

## Code availability

The process of radiomics feature extraction, deep learning model of pathomics training, and pathomics feature extraction were completed on Image managing platform supplied by United-Imaging® and Onekey platform respectively. The parameters and trained weights of deep learning model were available from the corresponding author, He Song. Email: hsong@cmu.edu.cn. In statistical analysis, R software (Ver. 4.2.1) and Python (Ver. 3.9.1) were utilized.
