# Author Correction: Development and interpretation of a multimodal predictive model for prognosis of gastrointestinal stromal tumor

**DOI:** 10.1038/s41698-024-00738-z

**Published:** 2024-10-21

**Authors:** XianHao Xiao, Xu Han, YeFei Sun, GuoLiang Zheng, Qi Miao, YuLong Zhang, JiaYing Tan, Gang Liu, QianRu He, JianPing Zhou, ZhiChao Zheng, GuiYang Jiang, He Song

**Affiliations:** 1 Department of Gastrointestinal Surgery, The First Hospital of China Medical University, Shenyang, Liaoning China; 2 Department of Pathology, The First Hospital and the College of Basic Medical Sciences of China Medical University, Shenyang, Liaoning China; 3 Department of Gastric Surgery, Cancer Hospital of China Medical University, Shenyang, Liaoning China; 4 Liaoning Cancer Hospital & Institute, Cancer Hospital of Dalian University of Technology, Shenyang, Liaoning China; 5 Department of Radiology, The First Hospital of China Medical University, Shenyang, Liaoning China; 6 The state Key laboratory of Neurology and Oncology Drug Development, Jiangsu Simcere Diagnostics Co., Ltd, Nanjing, China; 7 Department of Pathology, The College of Basic Medical Sciences and The First Hospital of China Medical University, Shenyang, Liaoning China

**Keywords:** Sarcoma, Risk factors

Correction to: *npj Precision Oncology* 10.1038/s41698-024-00636-4, published online 26 July 2024

“In this article, one of the corresponding authors, He Song, was listed incorrectly as the first author. This has now been corrected, and He Song is listed last in the author list.”

