# Supplementary material for: Development and interpretation of a multimodal predictive model for prognosis of gastrointestinal stromal tumor
Source: NPJ Precis Oncol. 2024 Jul 26;8:157. doi: 10.1038/s41698-024-00636-4 (PMC11282065; doi:10.1038/s41698-024-00636-4)
Supplement: Supplementary file 1 — Supplementary Files [file 41698_2024_636_MOESM1_ESM.pdf]

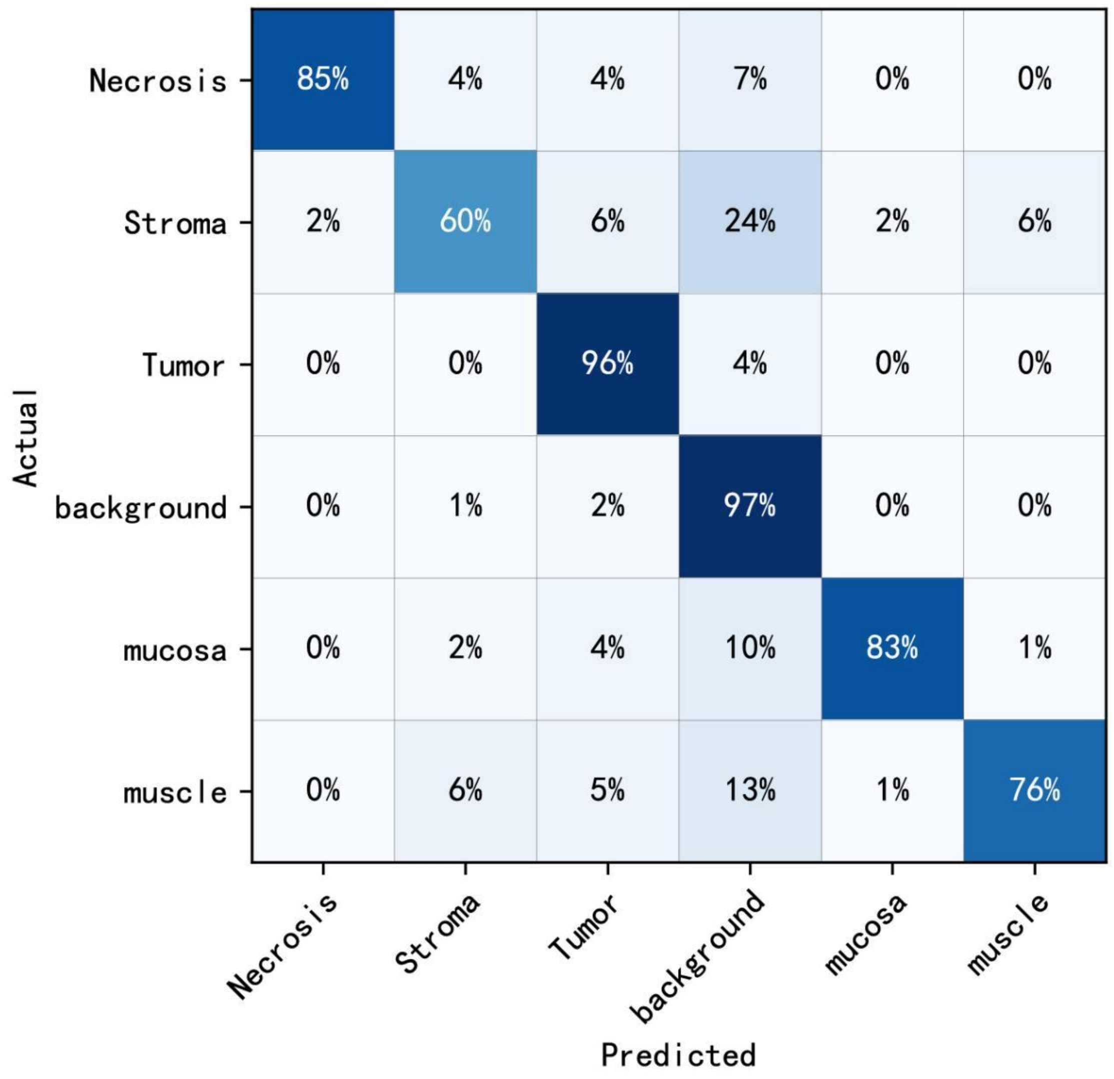

Supplementary Figure 1. Confusion matrix of the prediction generated by the classification model based on deep learning in the best-performing epoch.

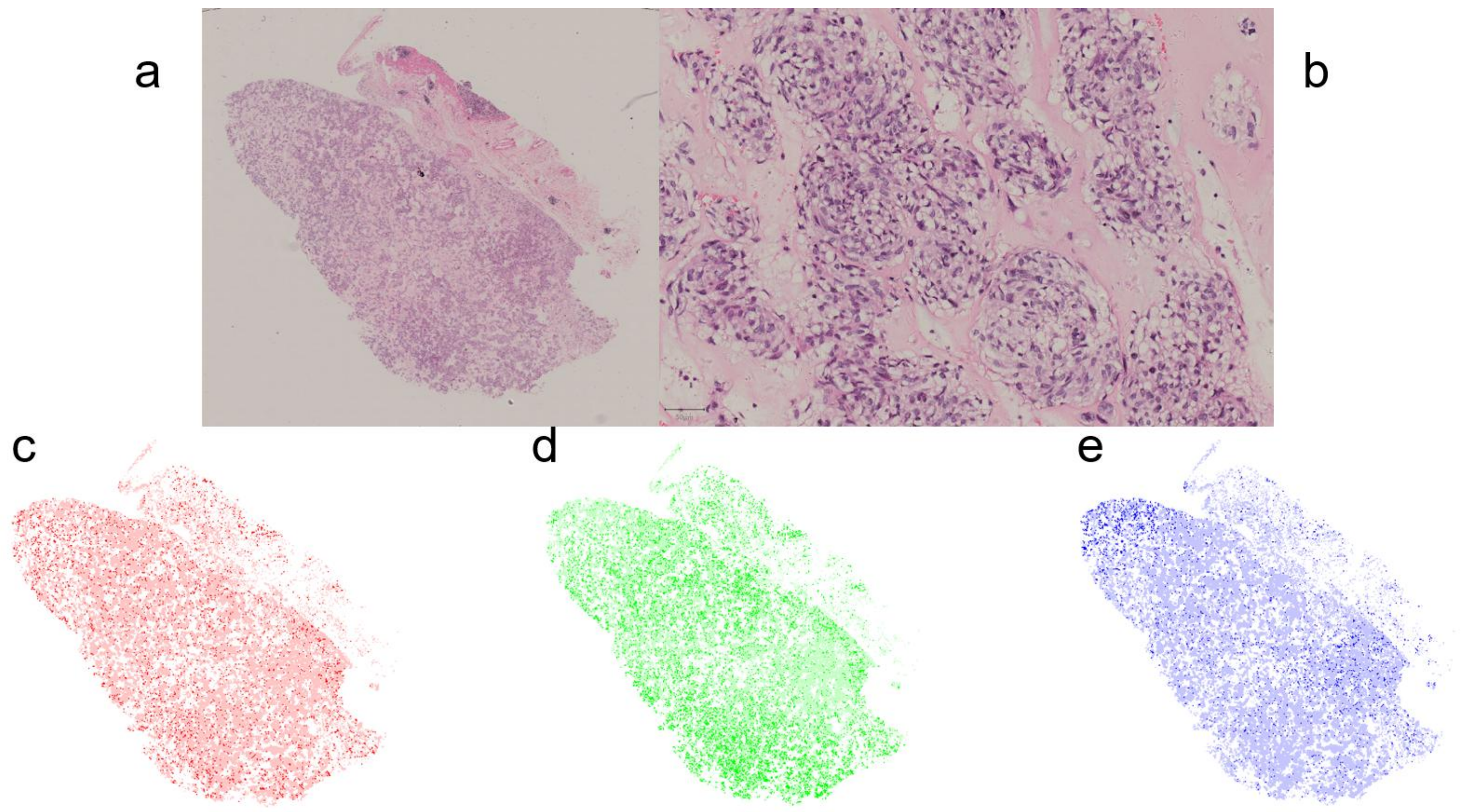
a
b
c
d
e

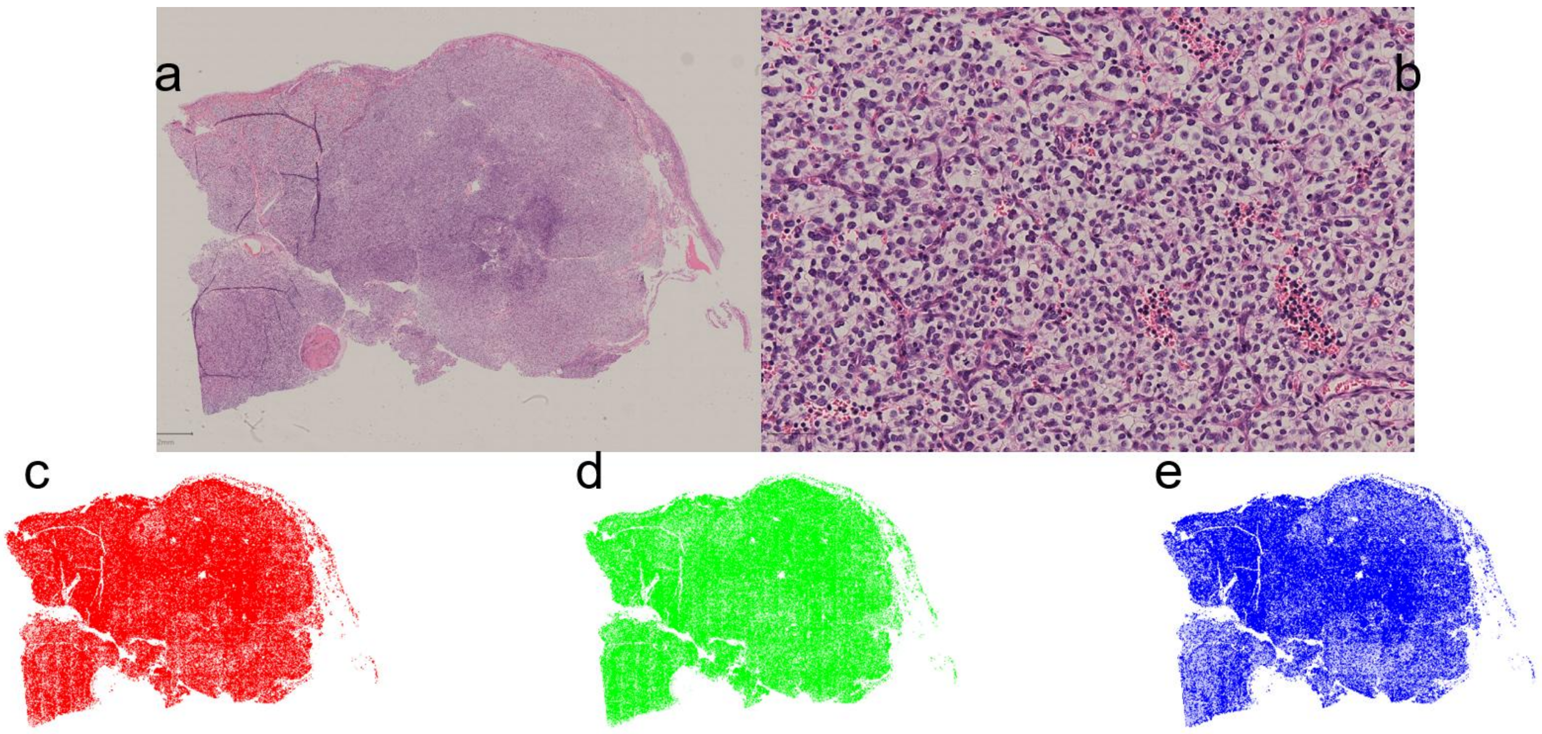
a
b
c
d
e

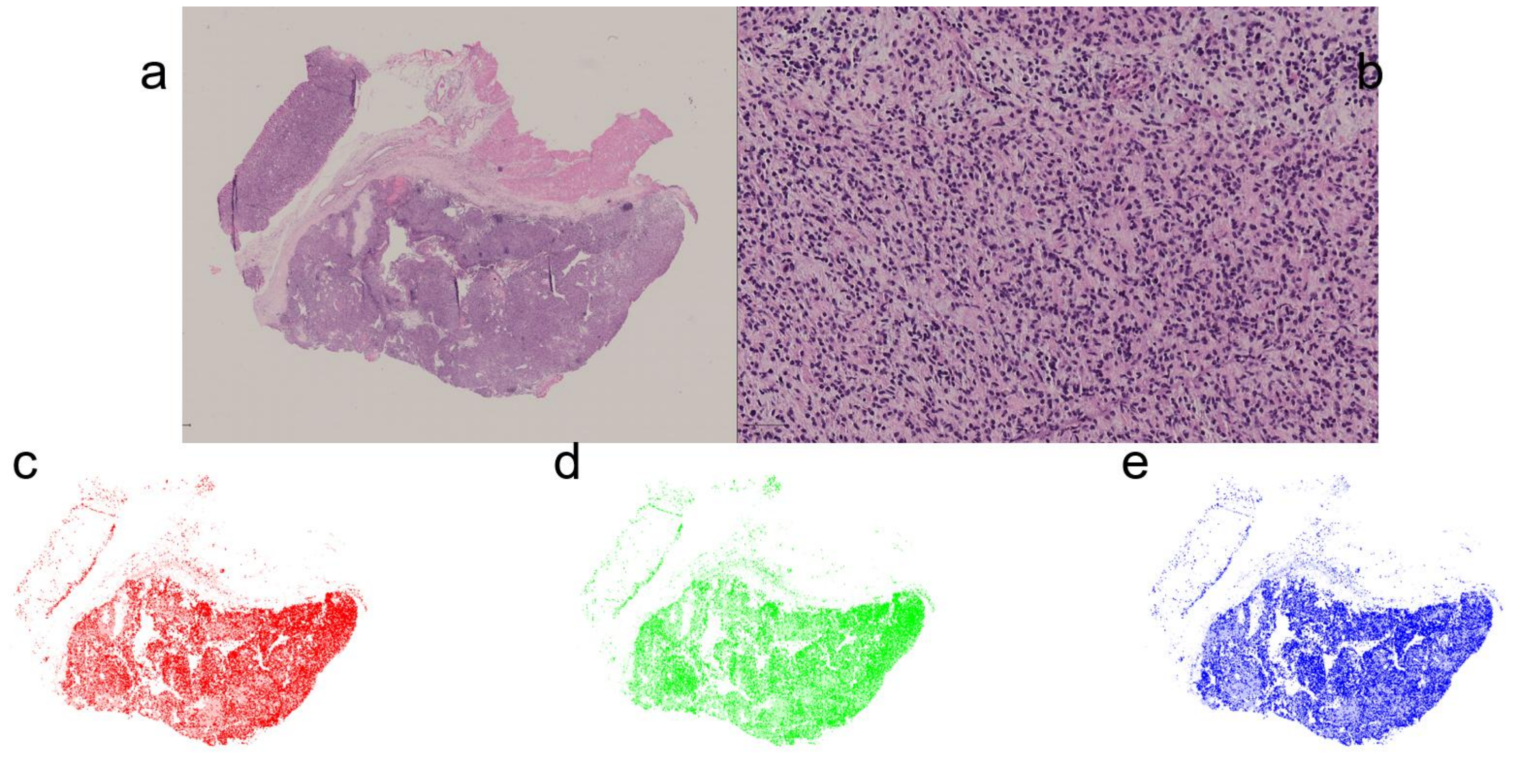
a
b
c
d
e

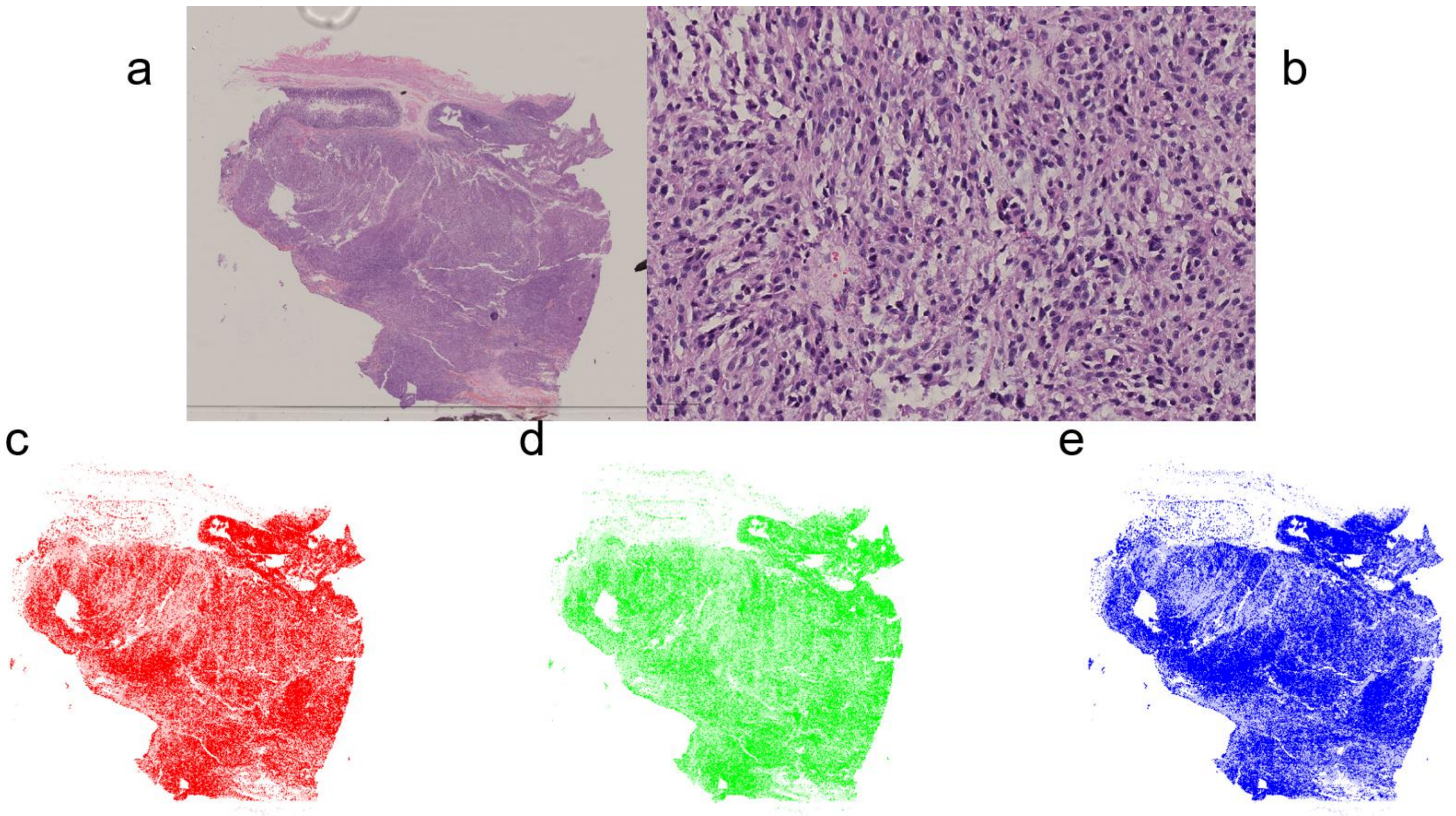
a
b
c
d
e

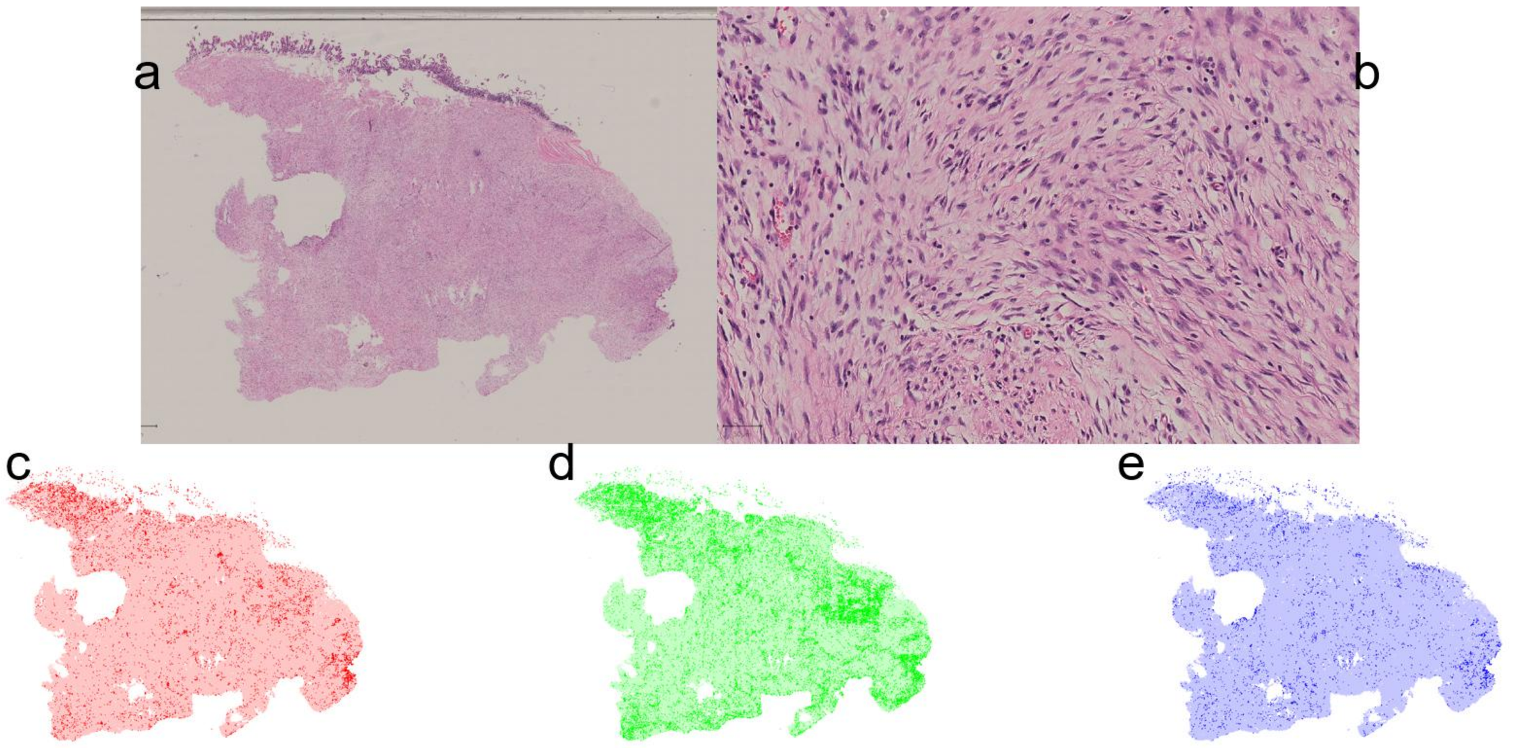
a
b
c
d
e

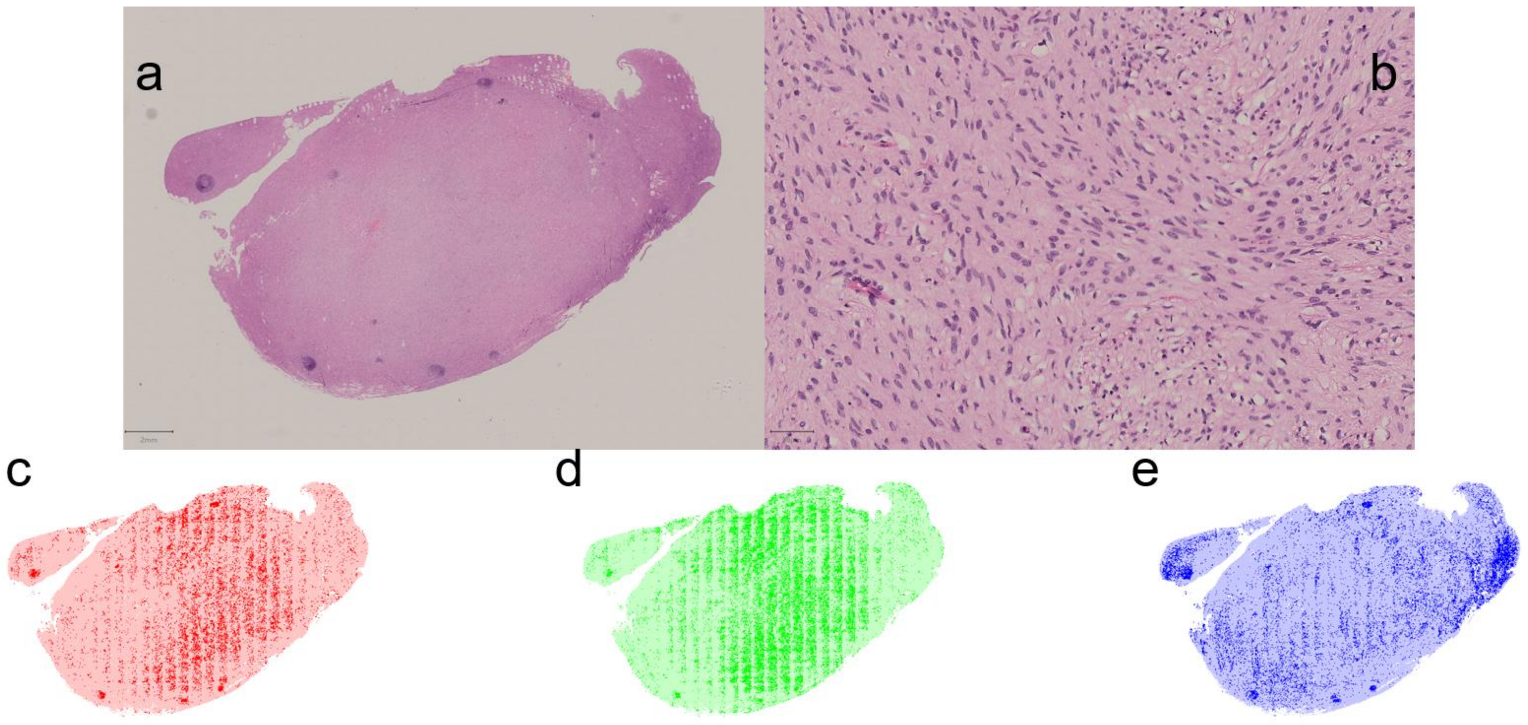
a
b
c
d
e

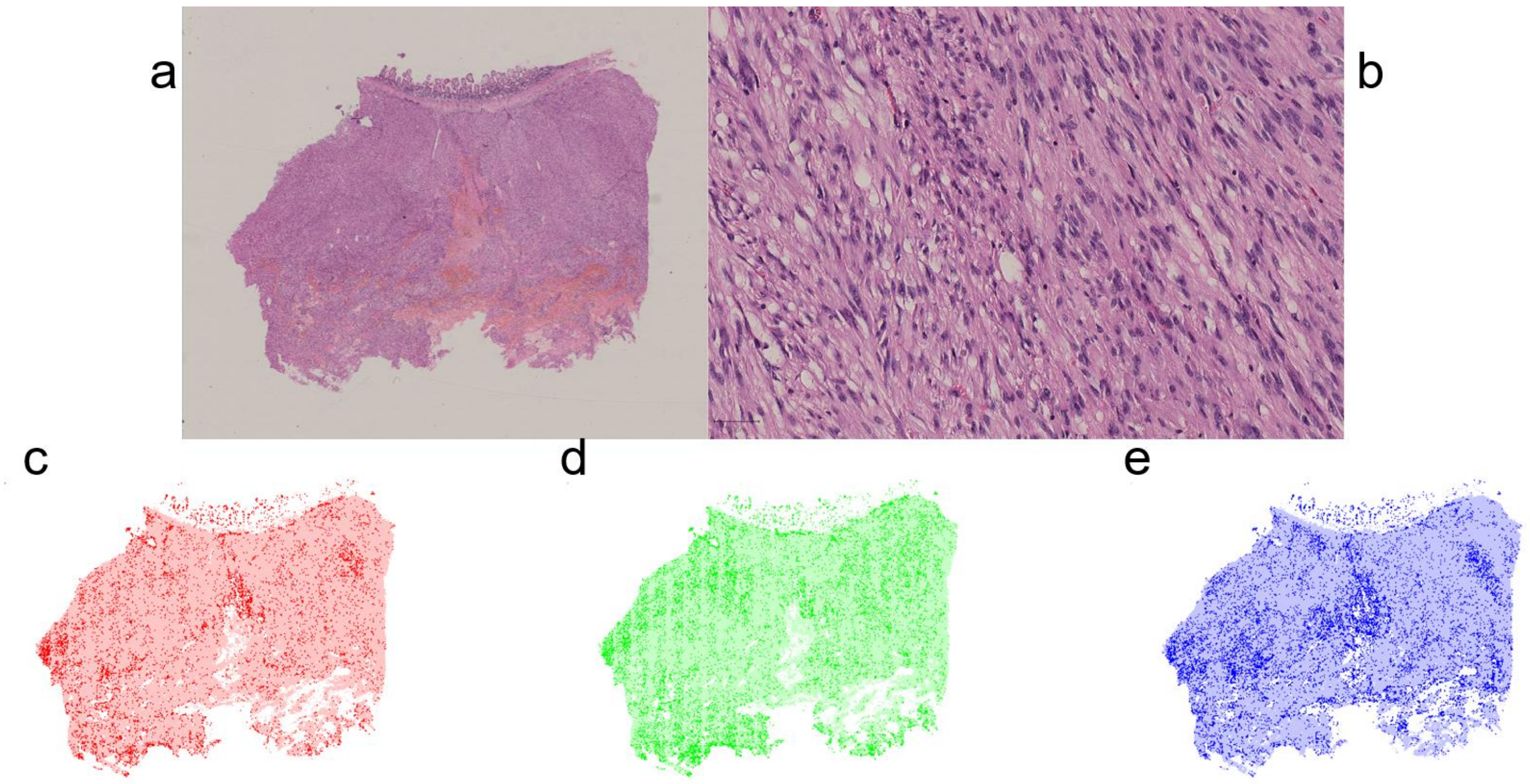
a
b
c
d
e

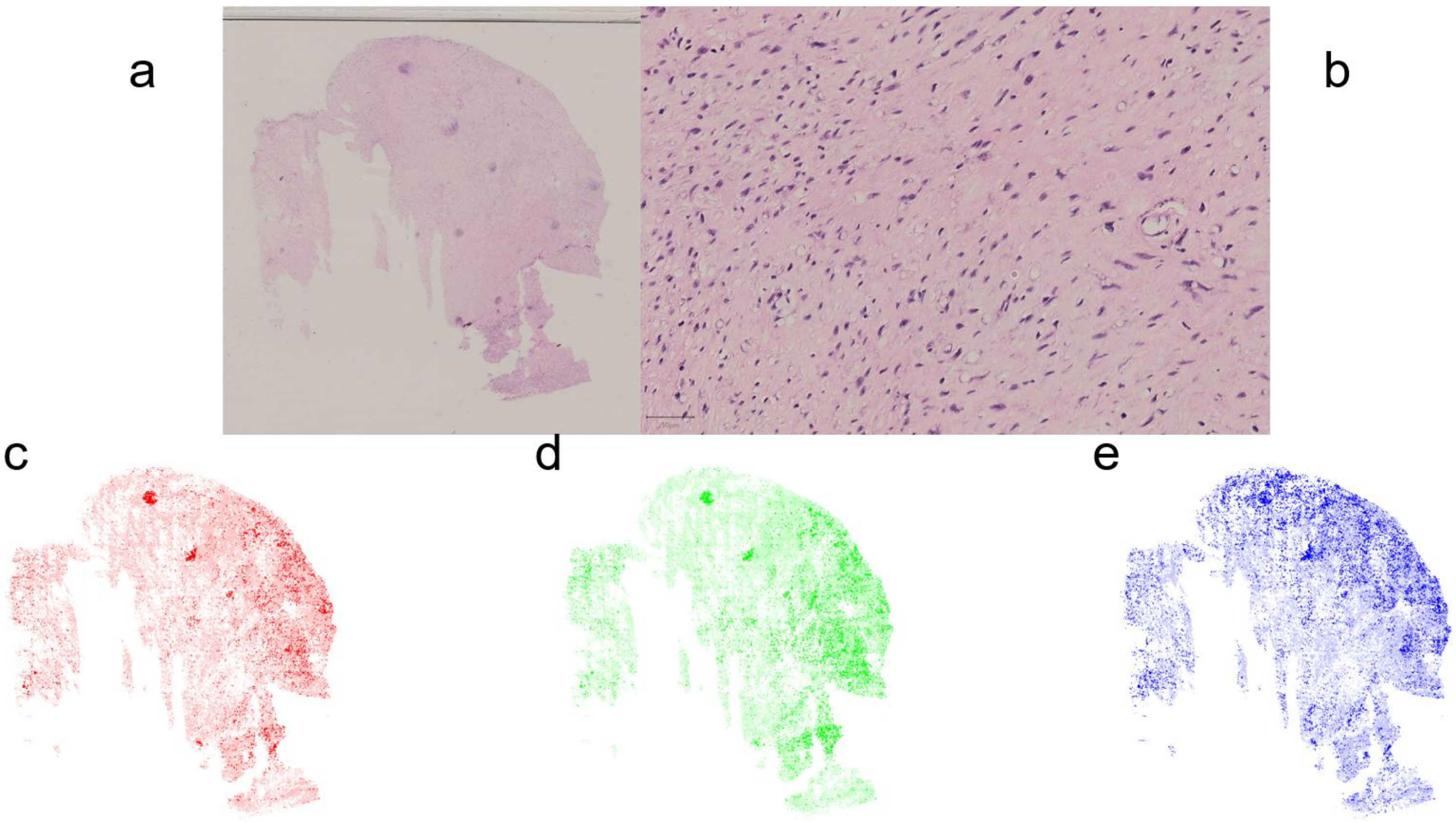
a
b
c
d
e

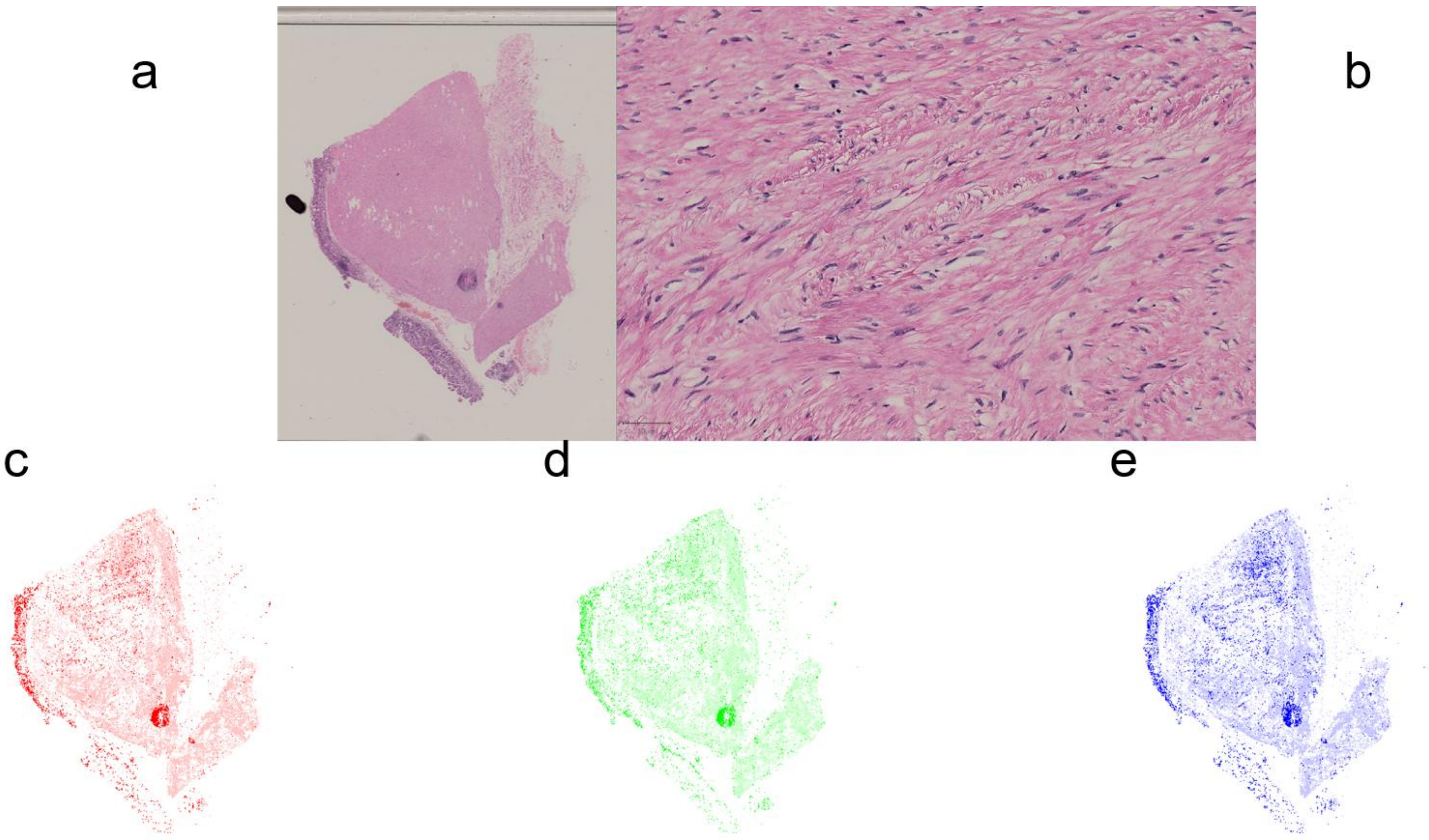
a
b
c
d
e

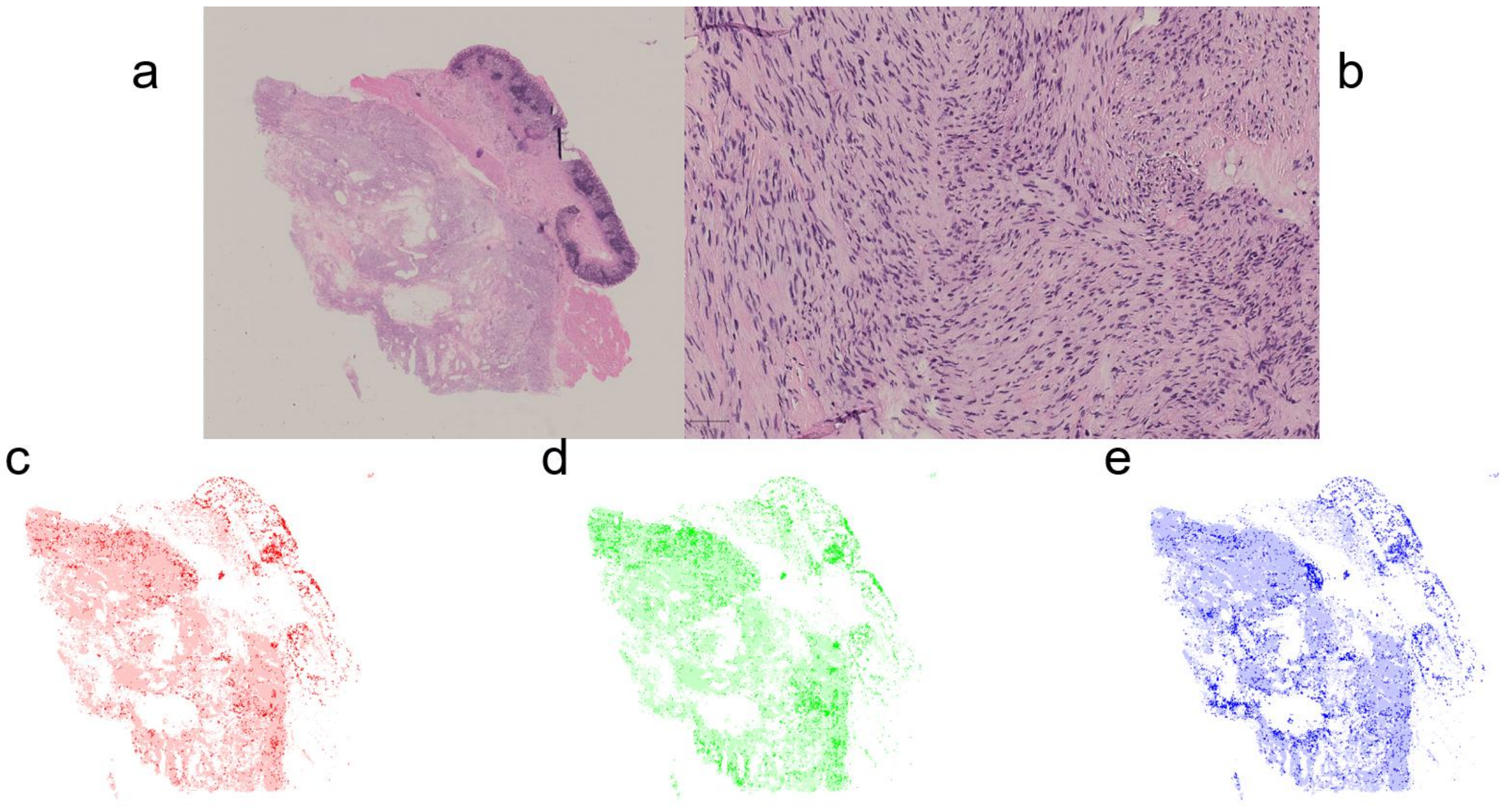
a
b
c
d
e

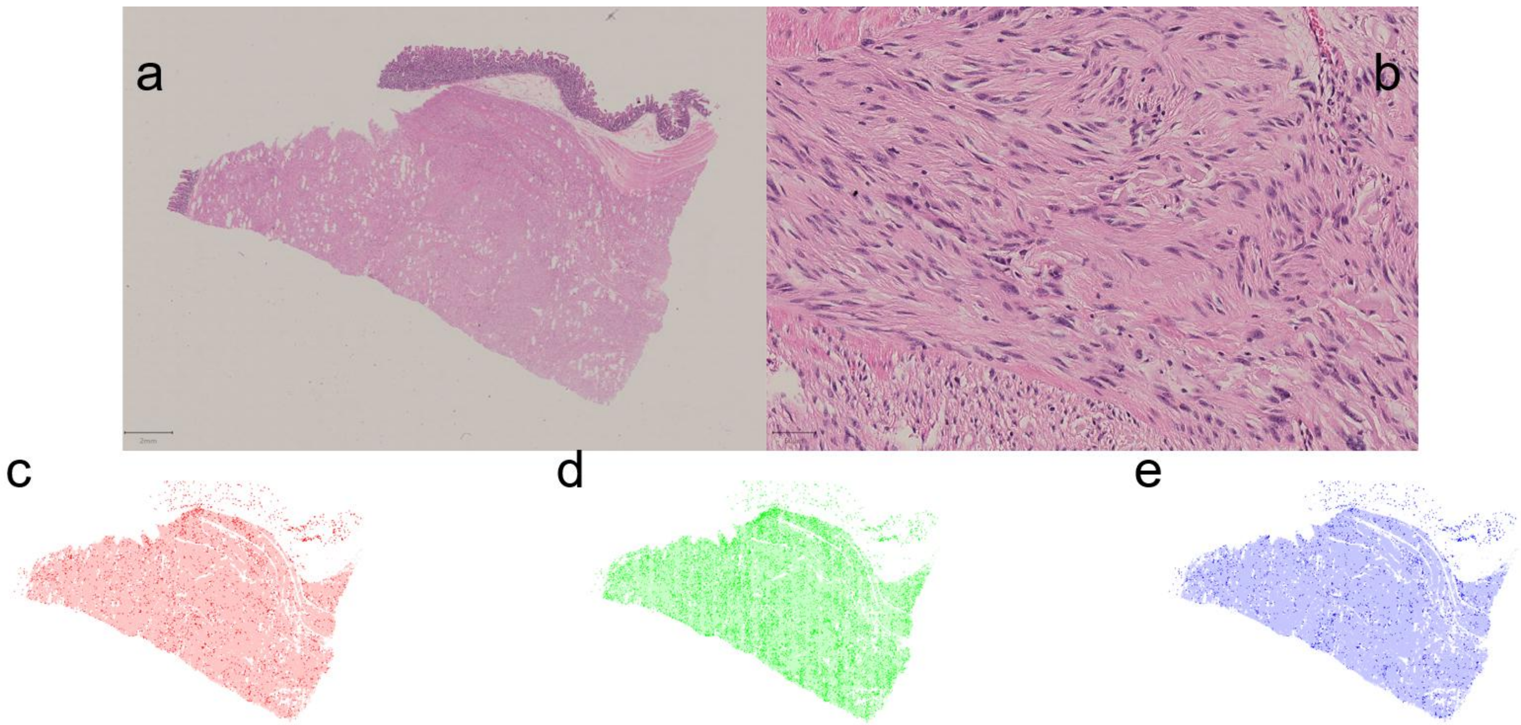
a
b
c
d
e

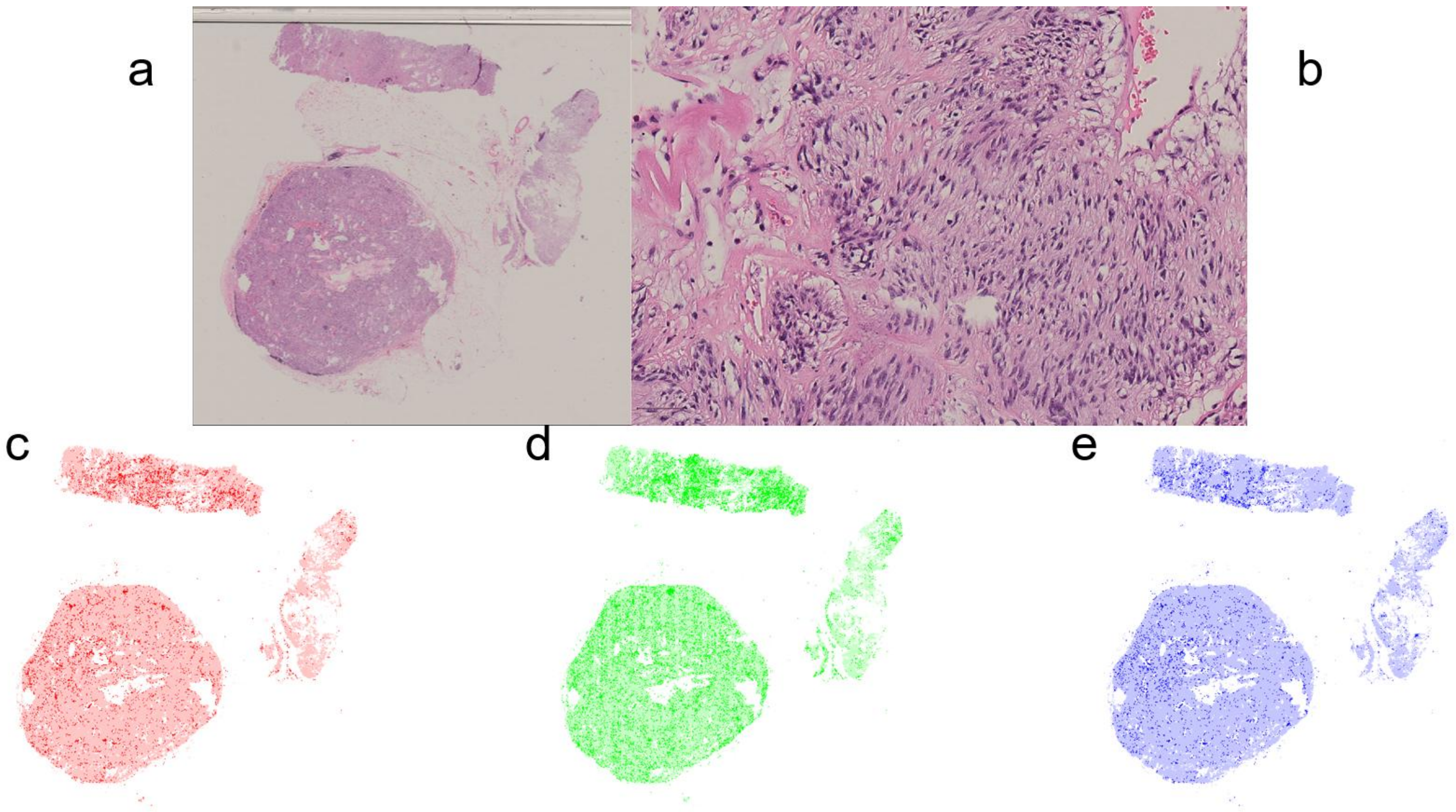
a
b
c
d
e

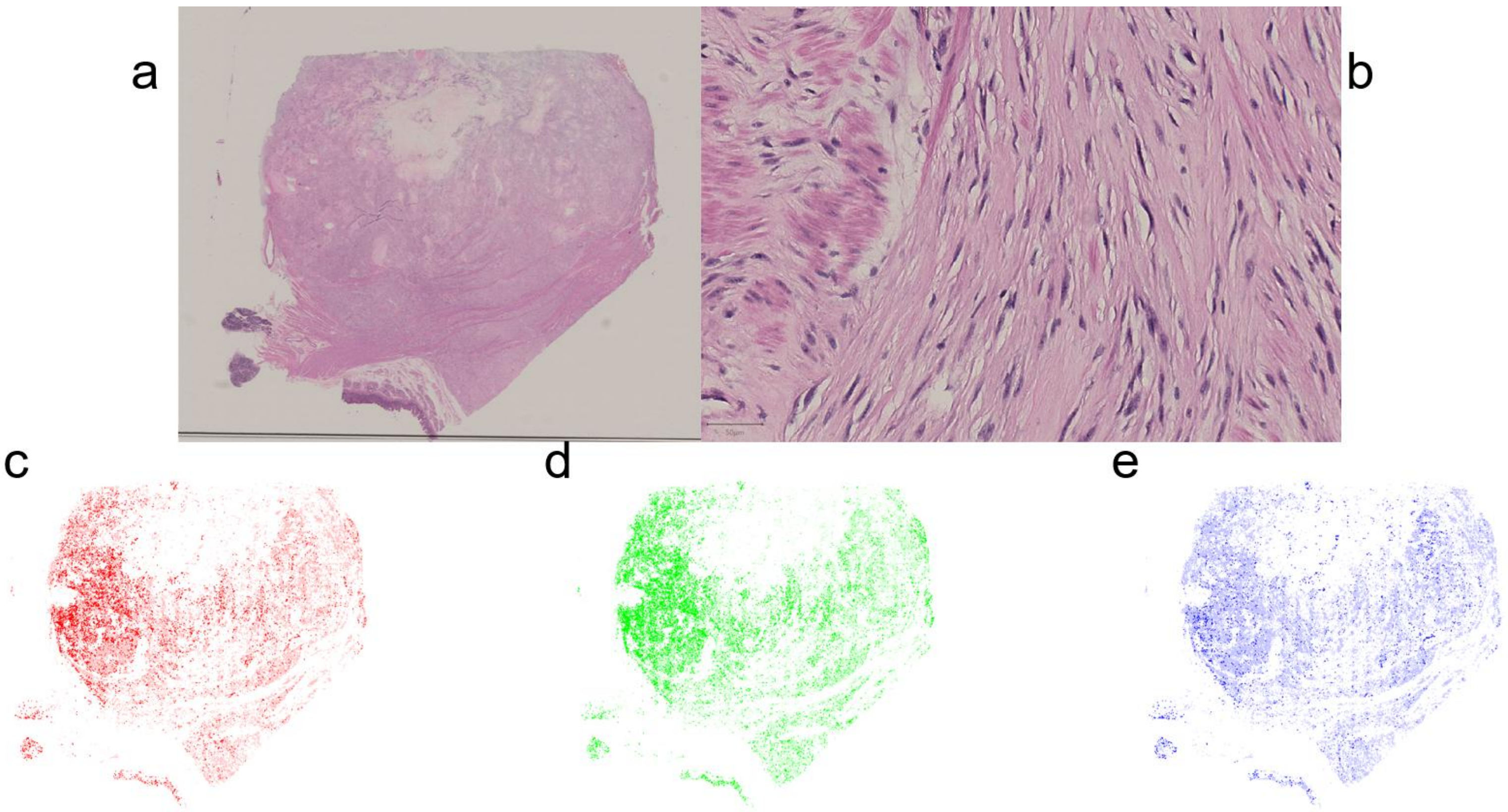
a
b
c
d
e

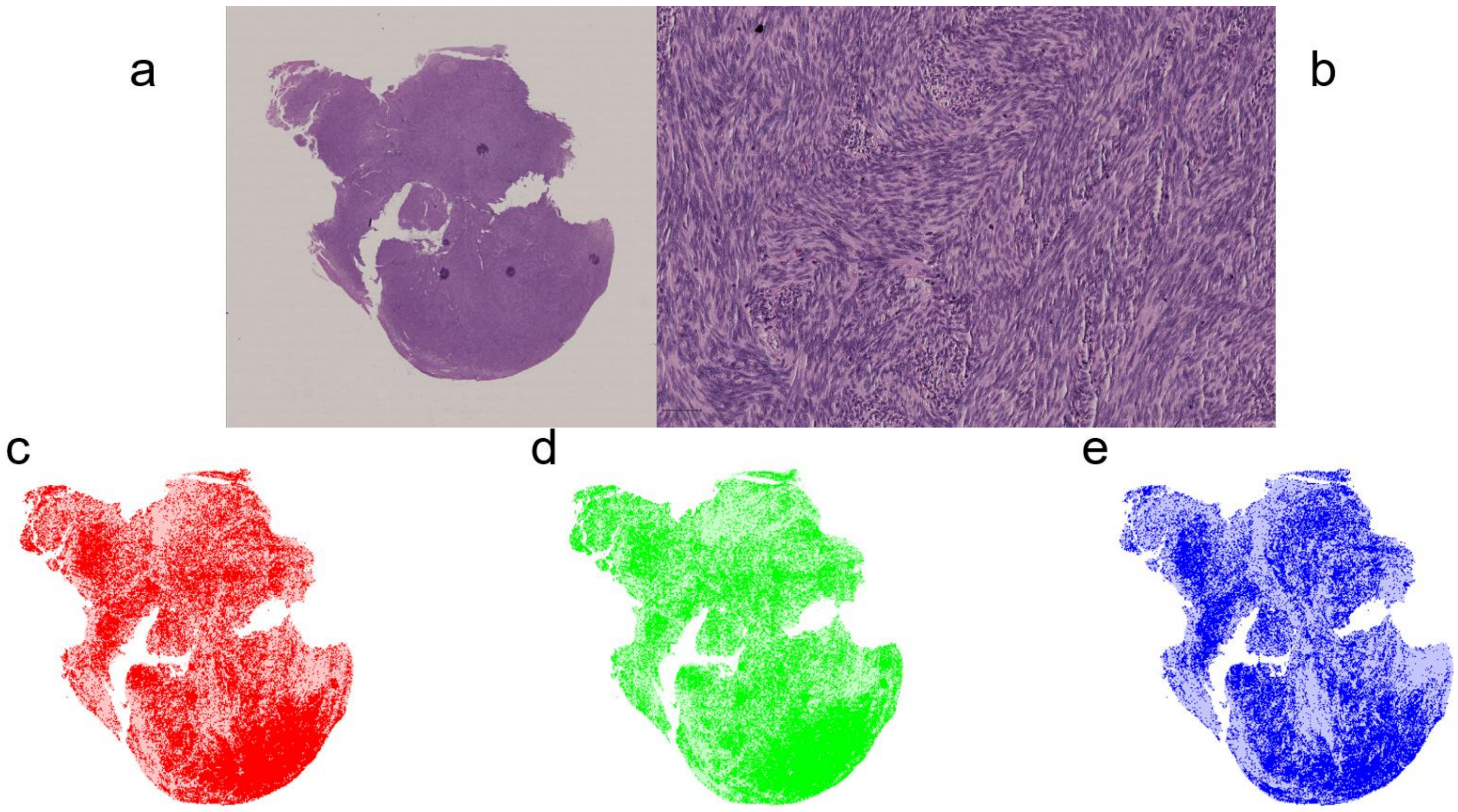
a
b
c
d
e

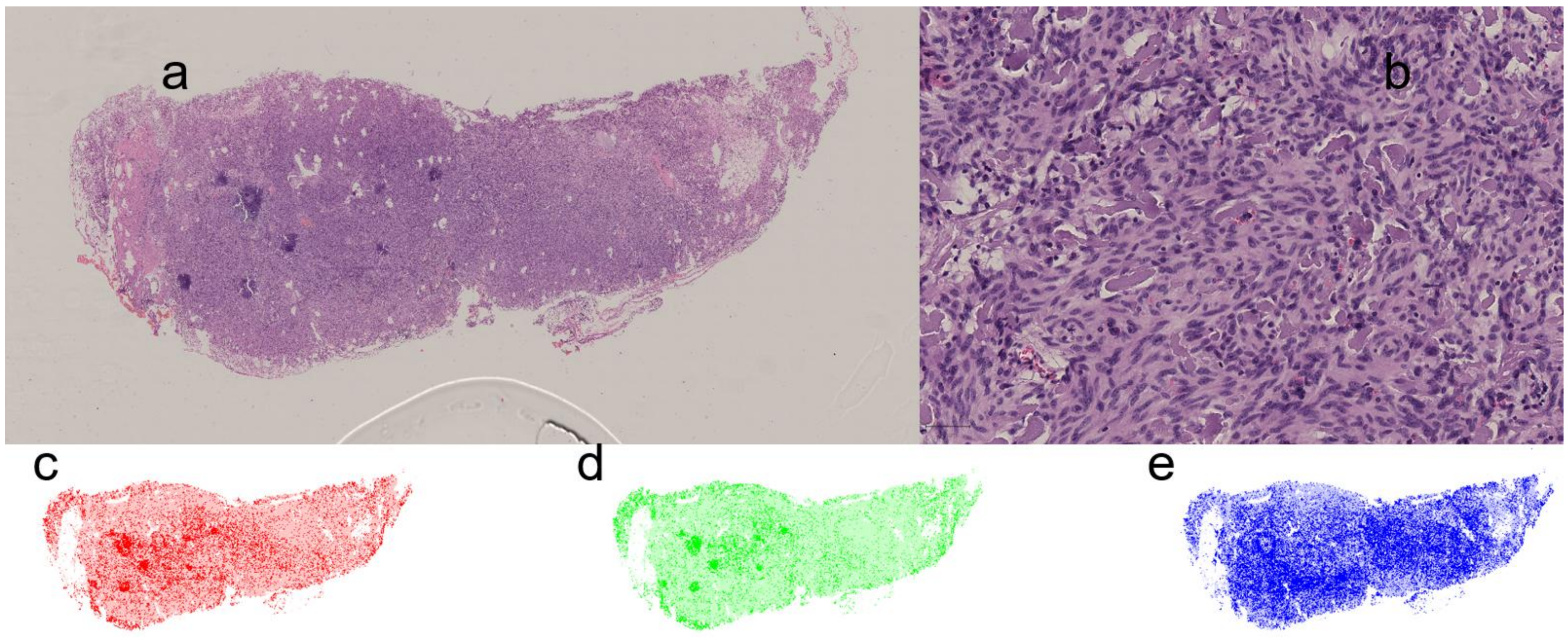
a
b
c
d
e

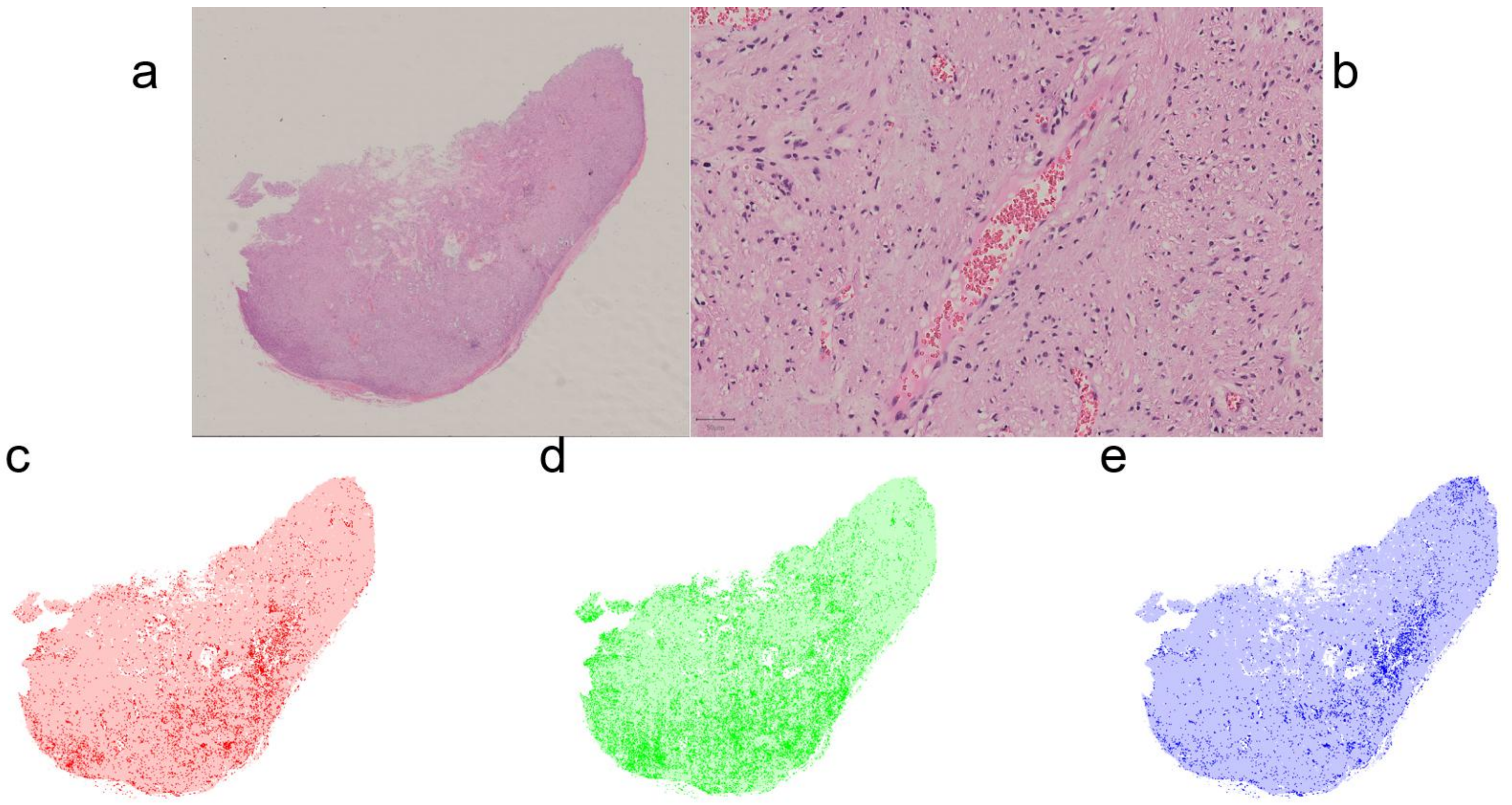
a
b
c
d
e

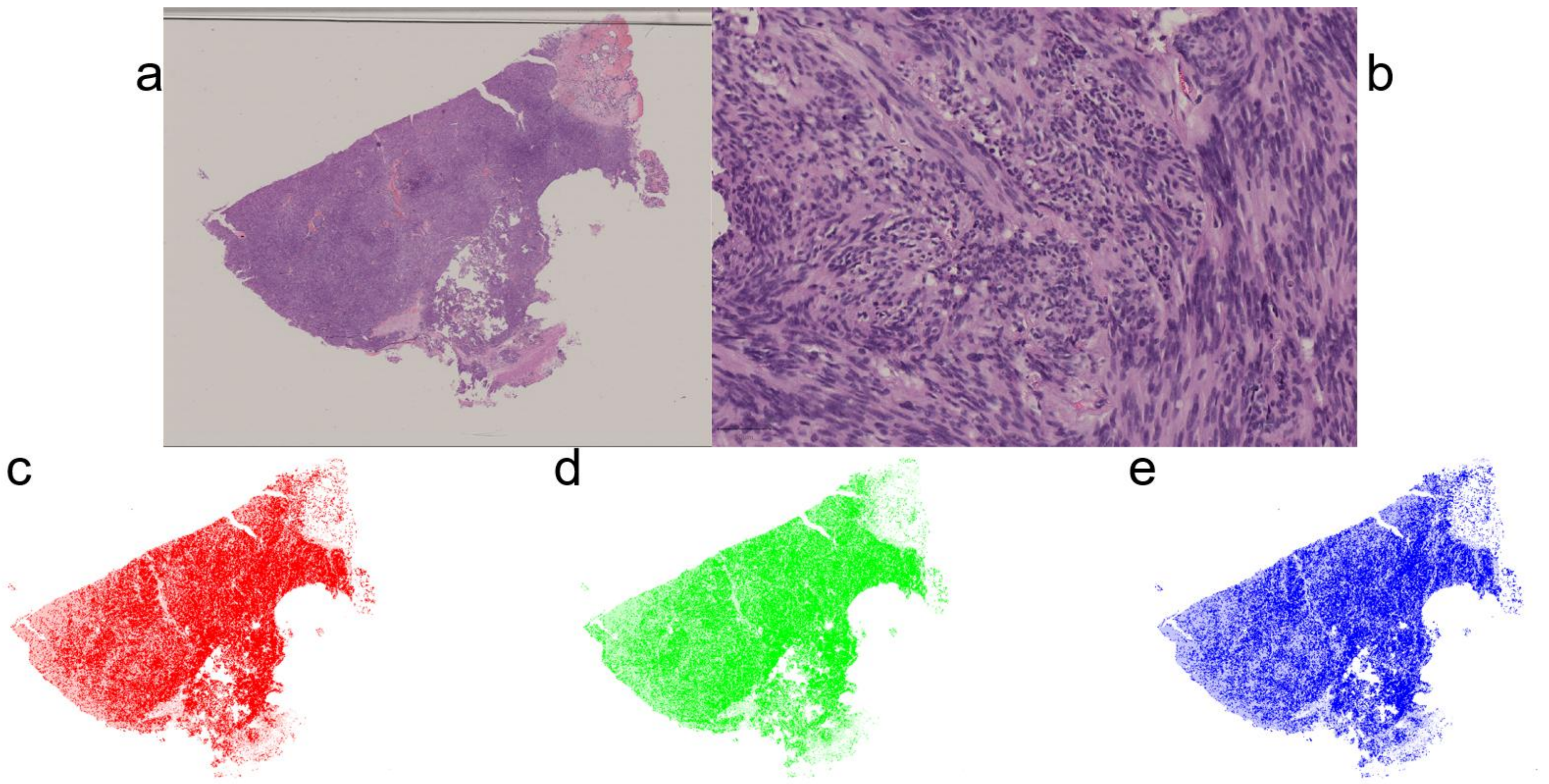
a
b
c
d
e

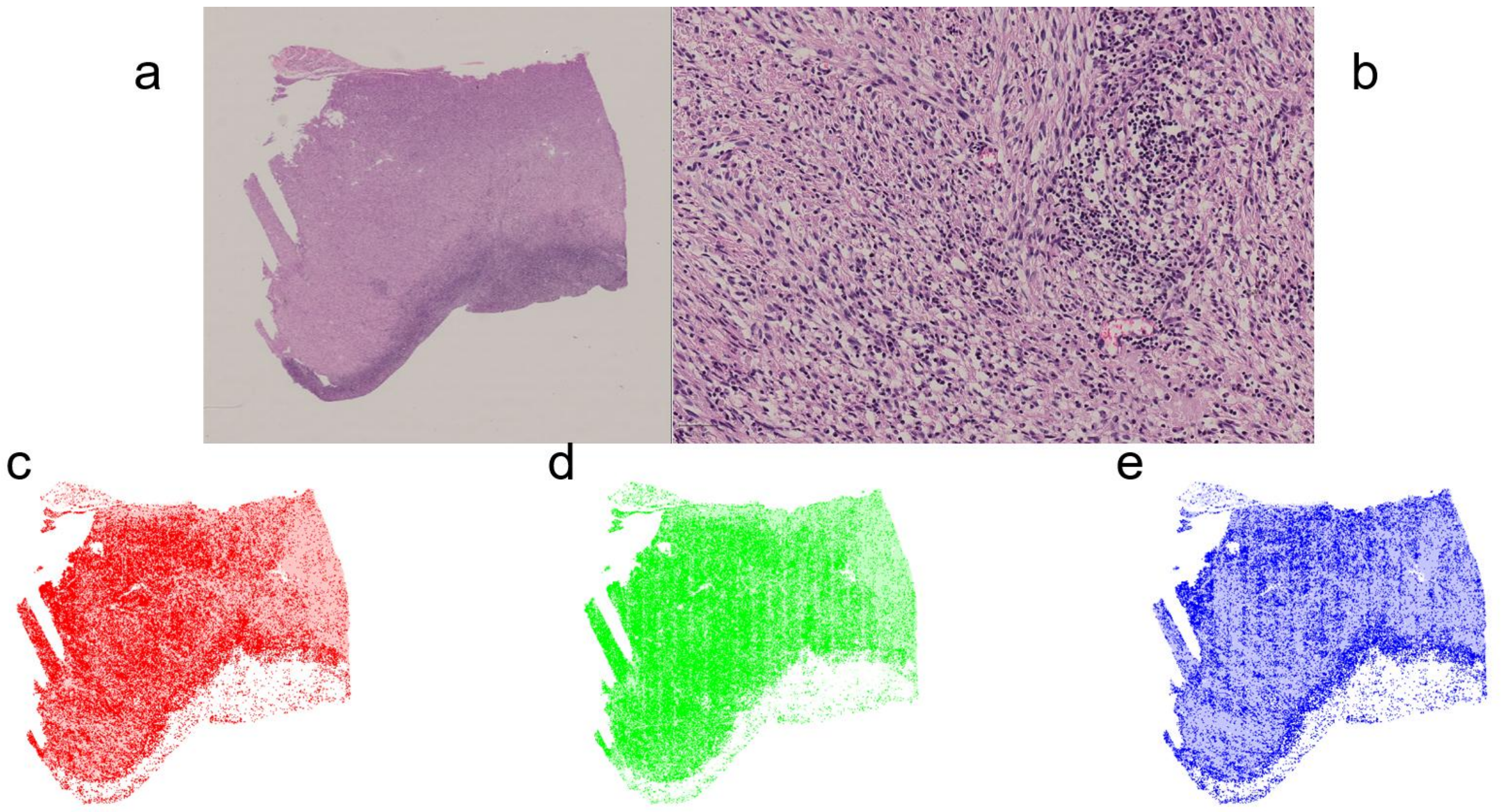
a
b
c
d
e

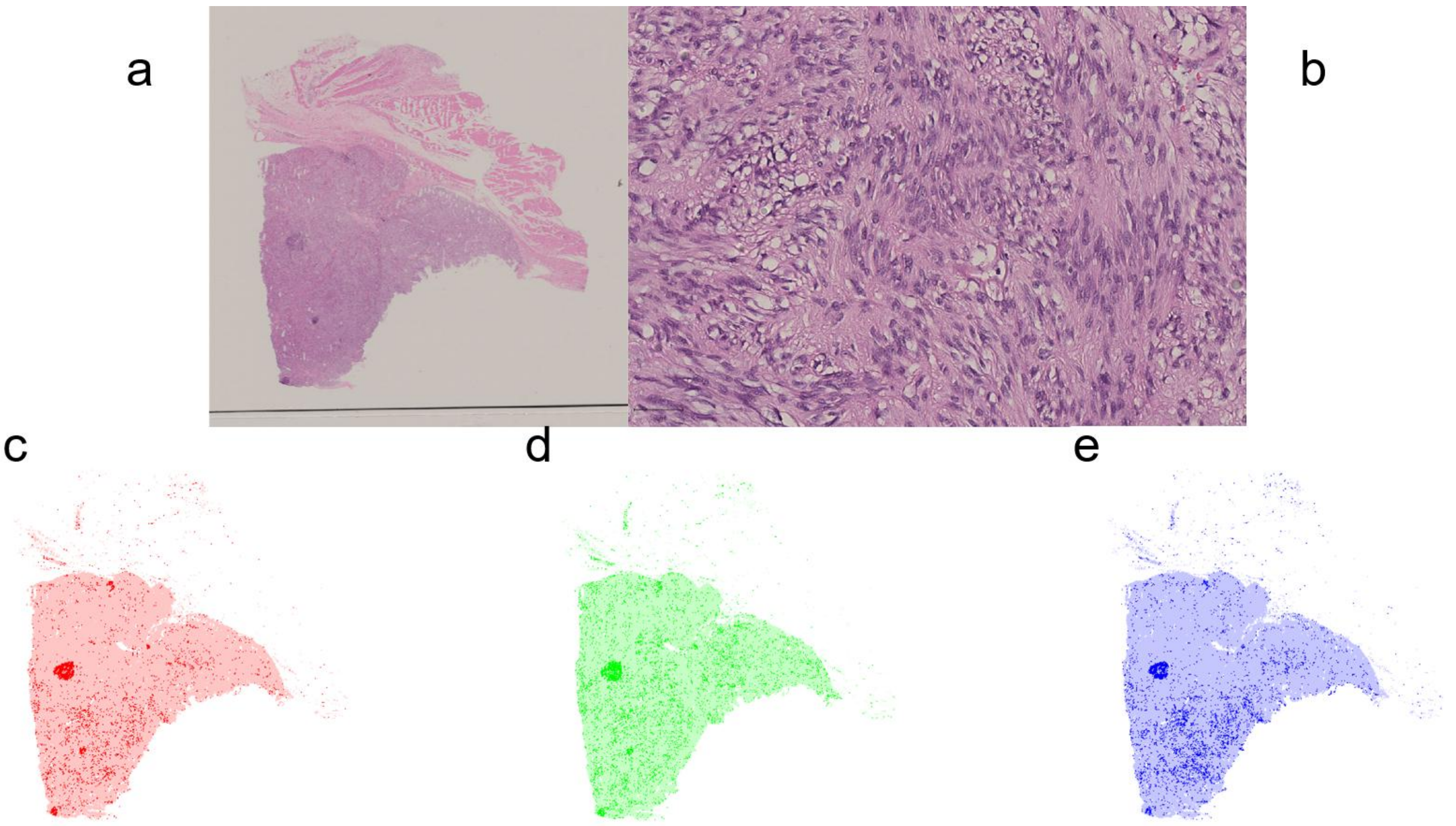

a b

c d e

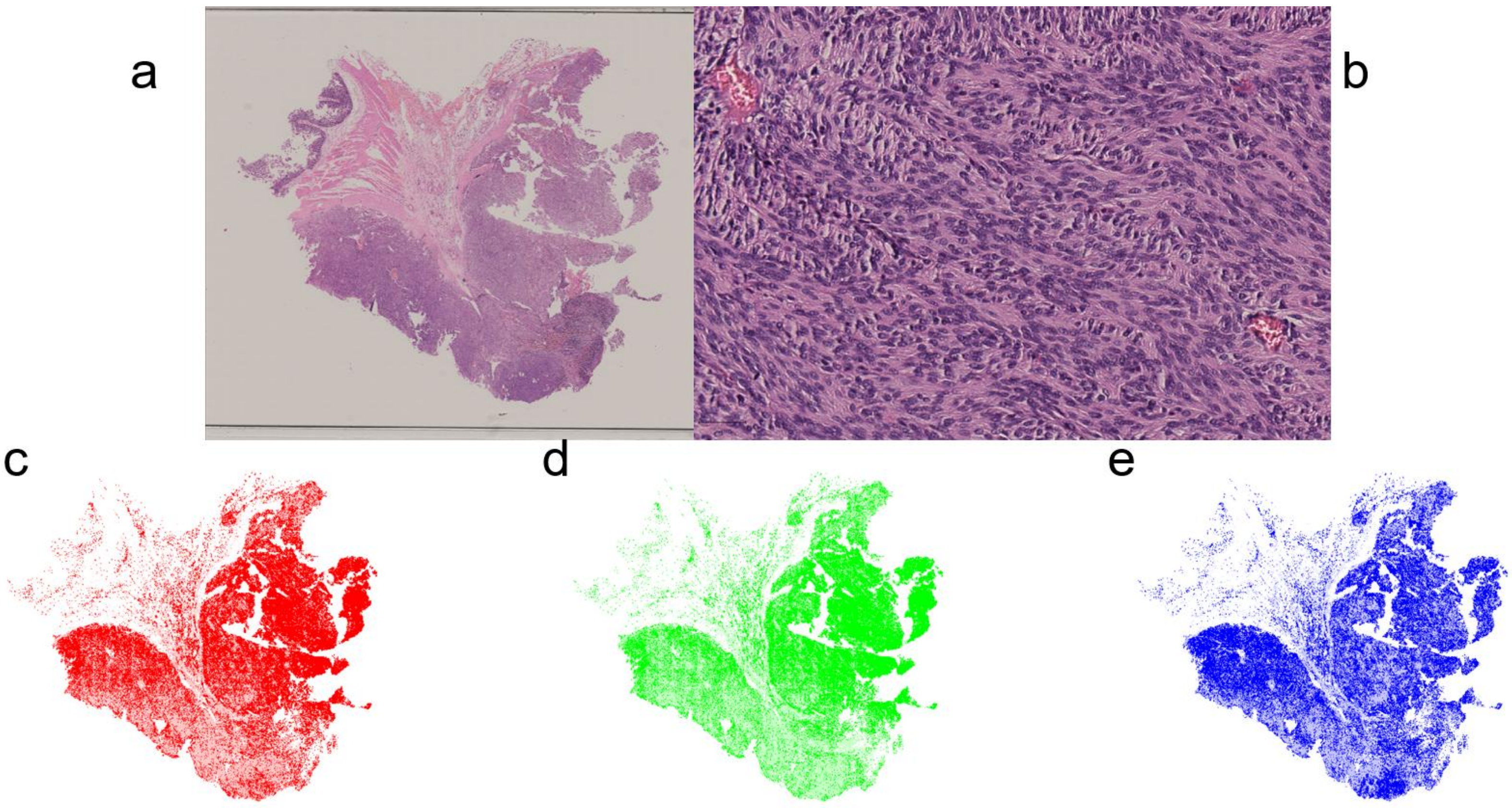

a b

c d e

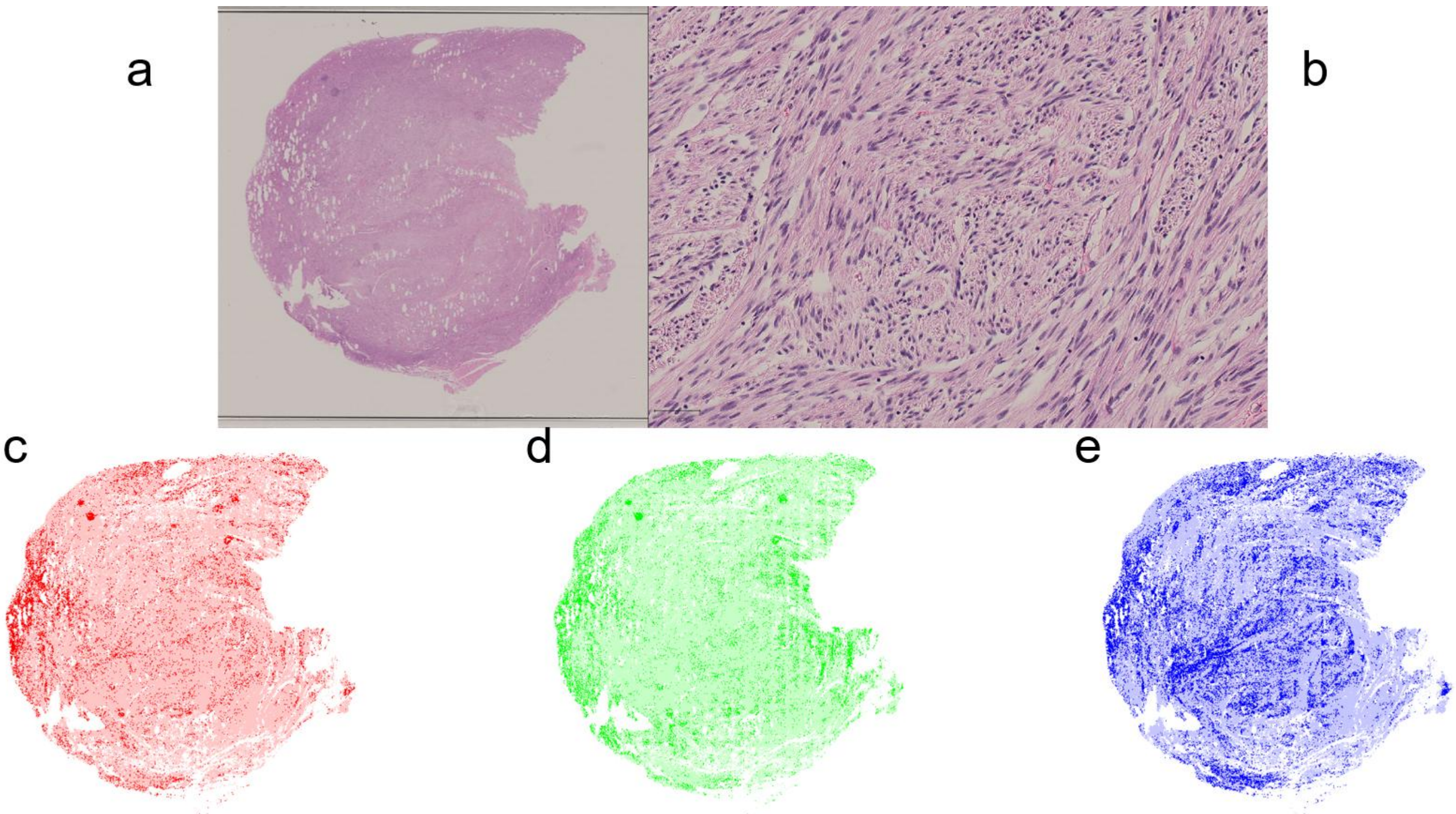

a b

c d e

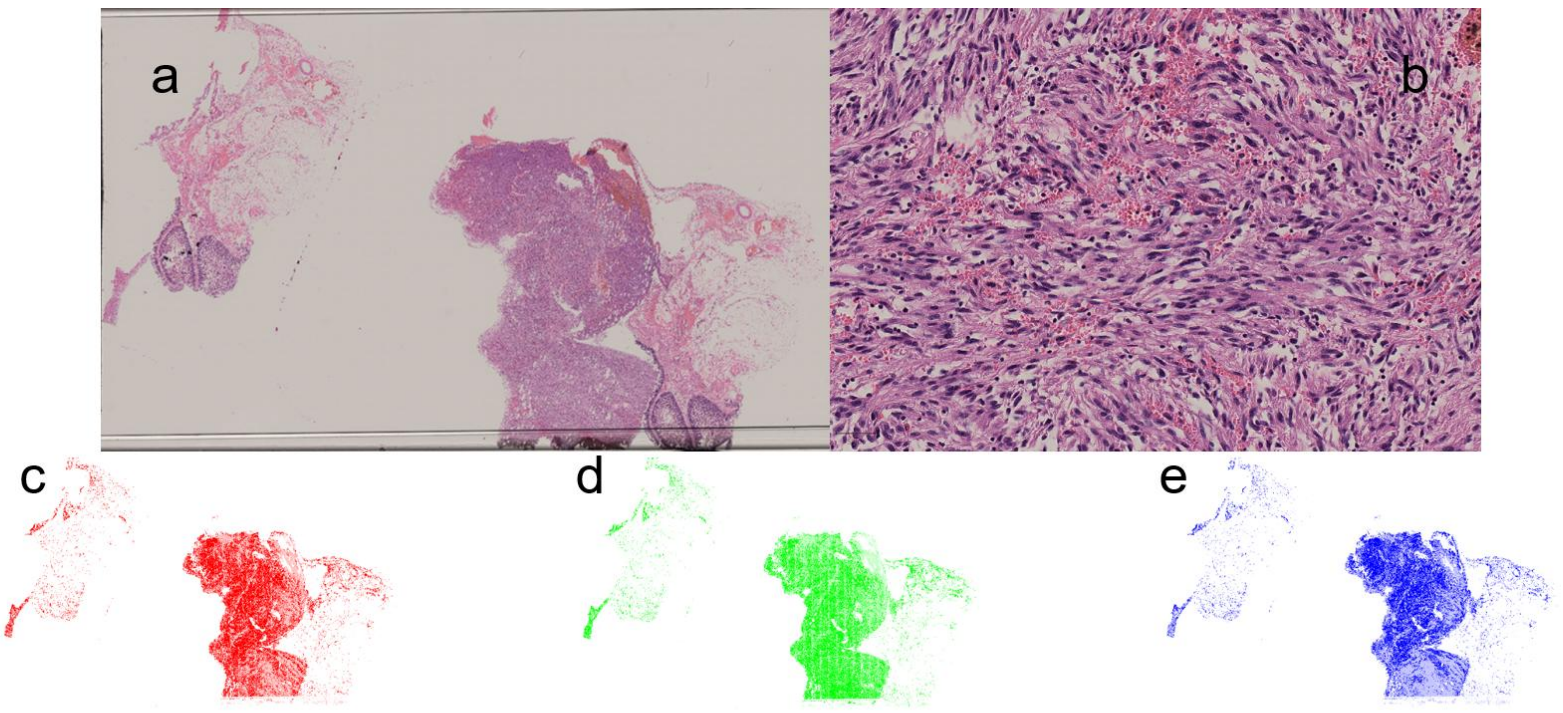
a
b
c
d
e

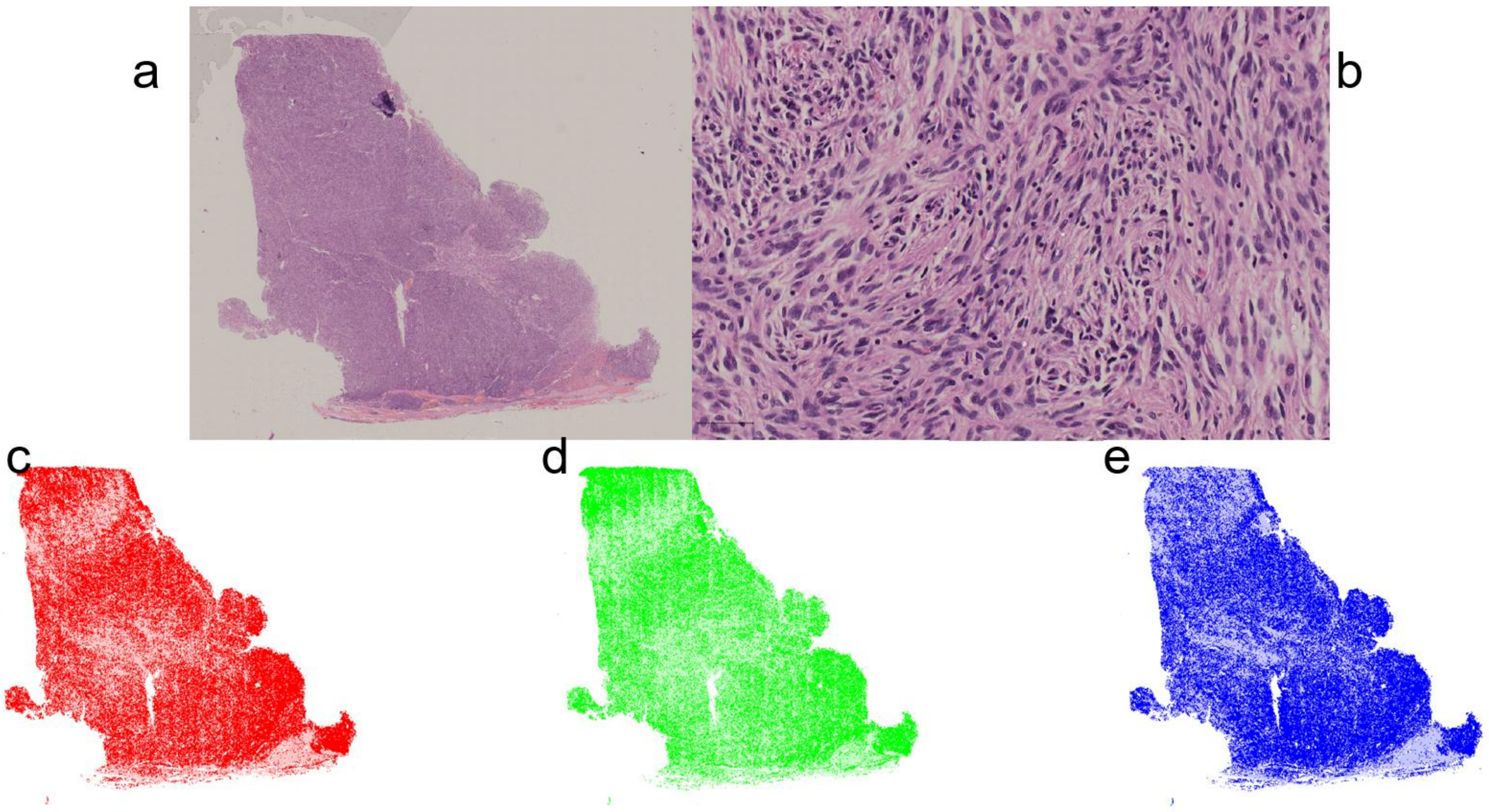
a
b
c
d
e

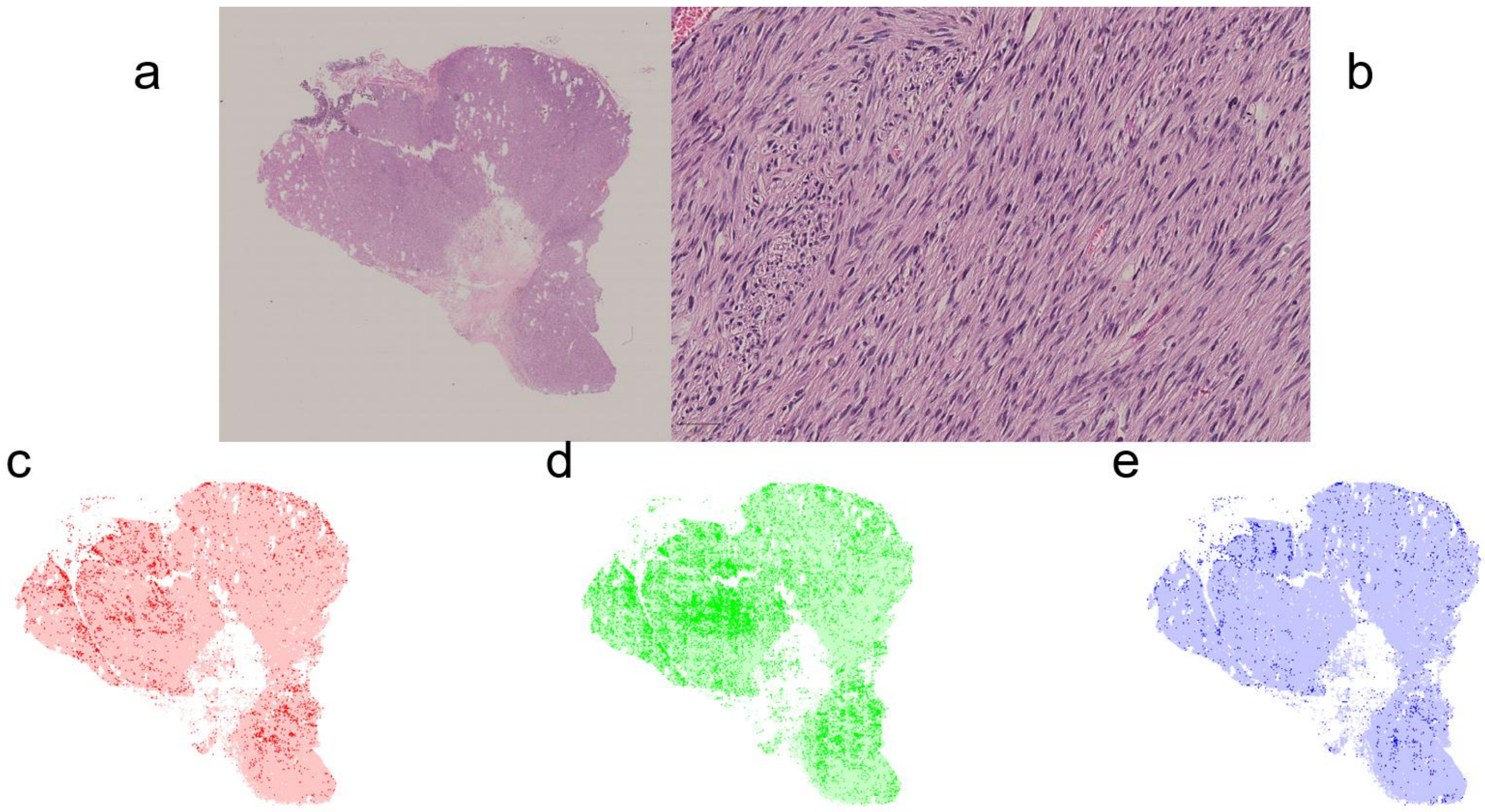
a
b
c
d
e

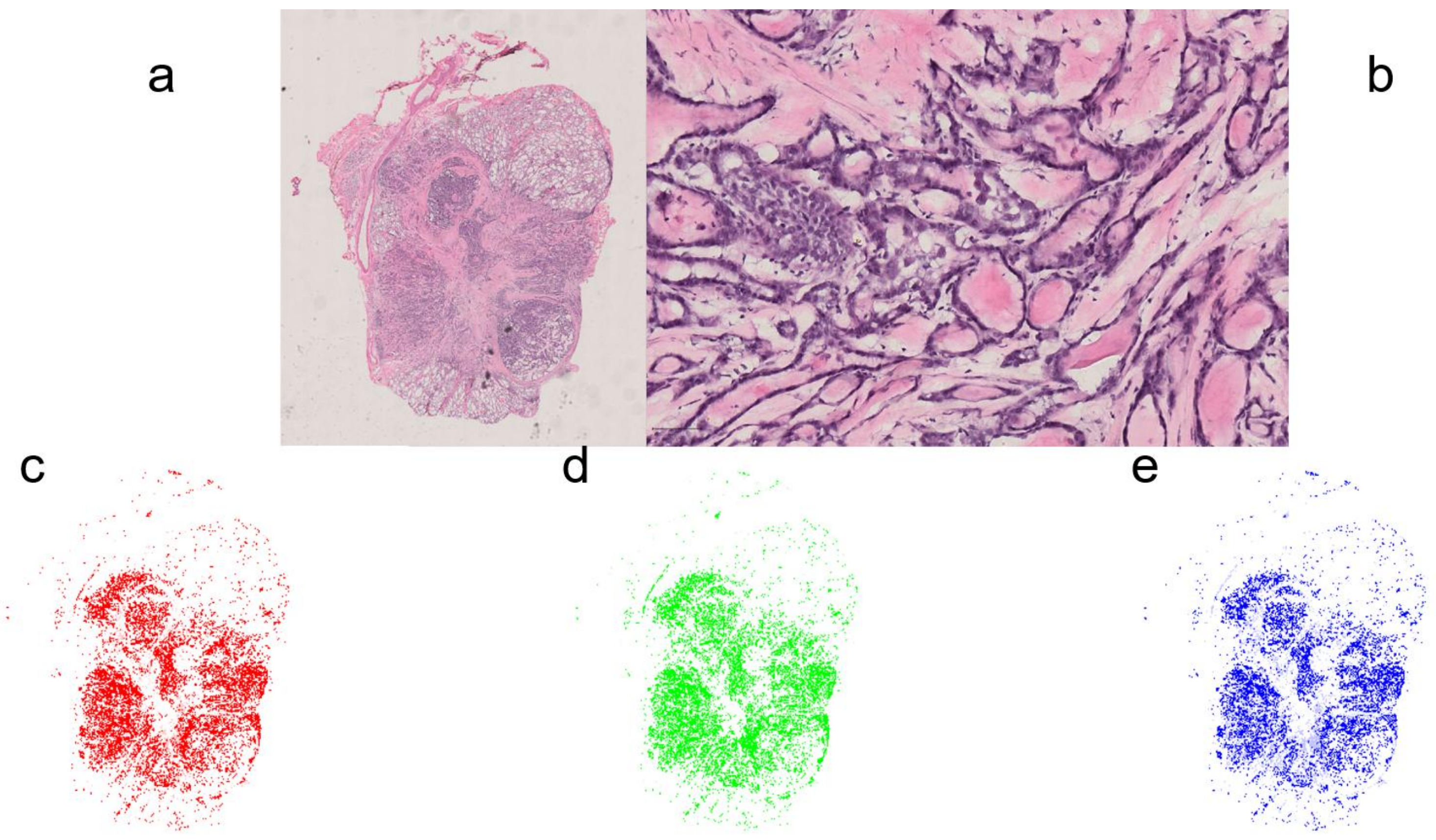
a
b
c
d
e

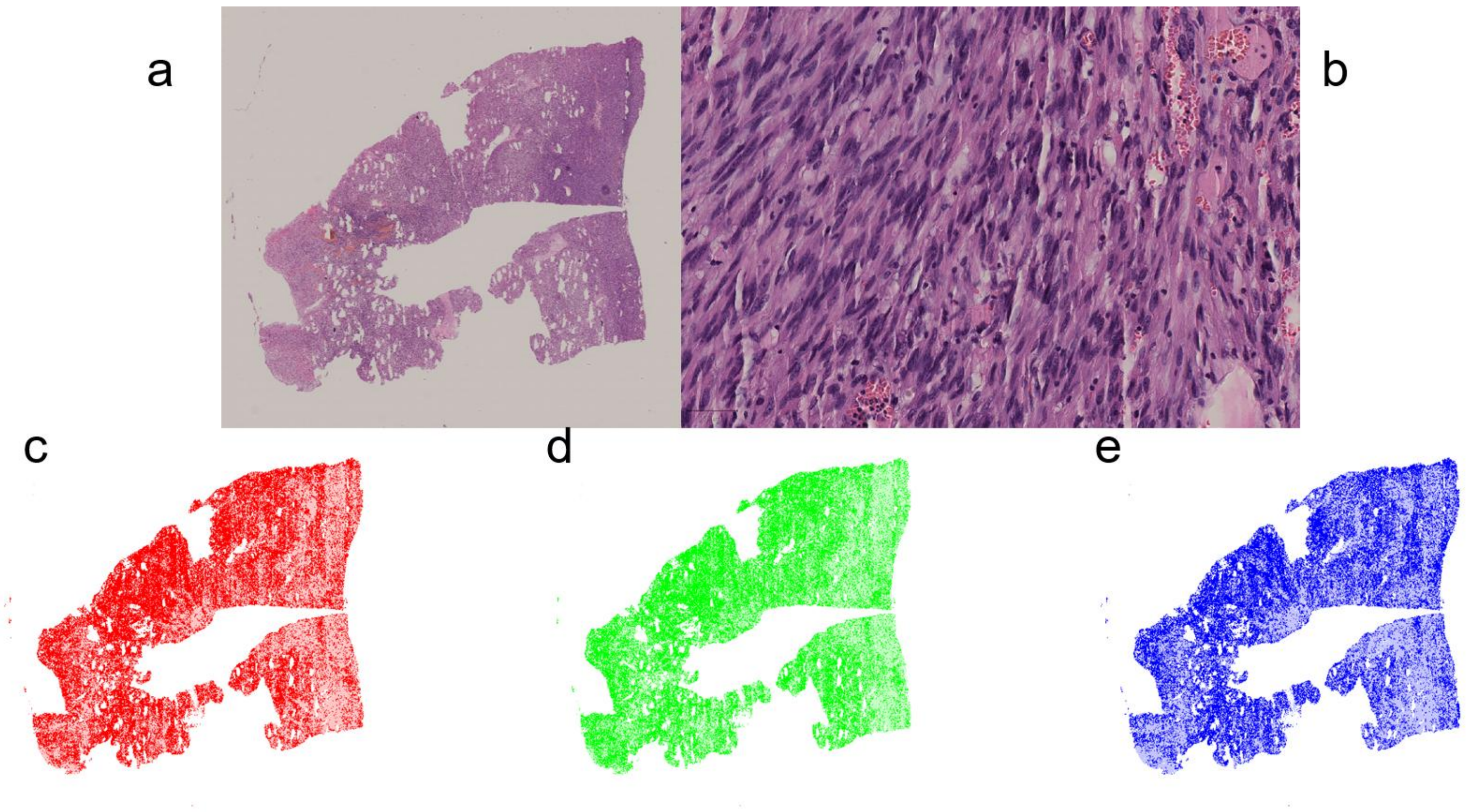
a
b
c
d
e

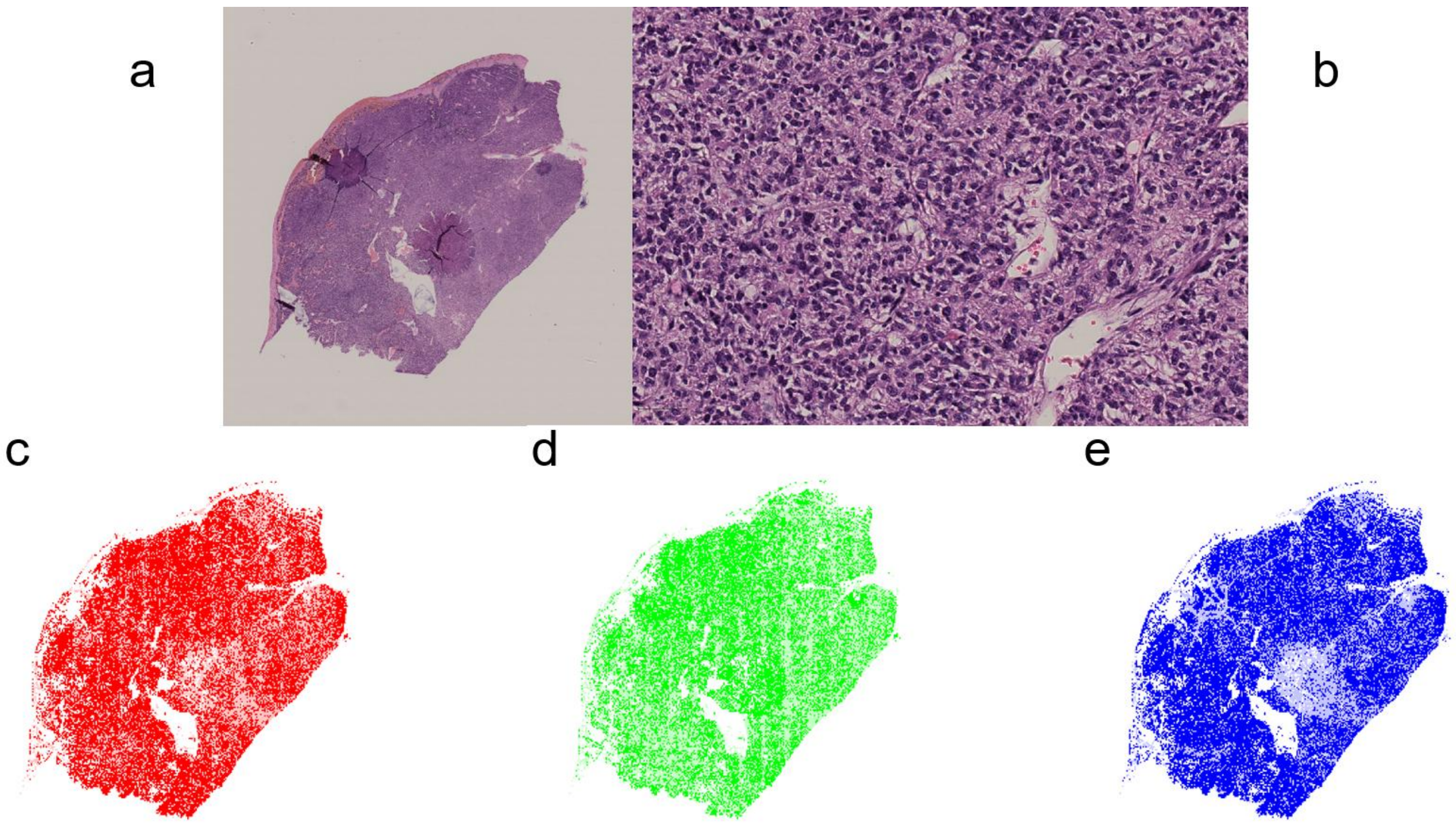
a
b
c
d
e

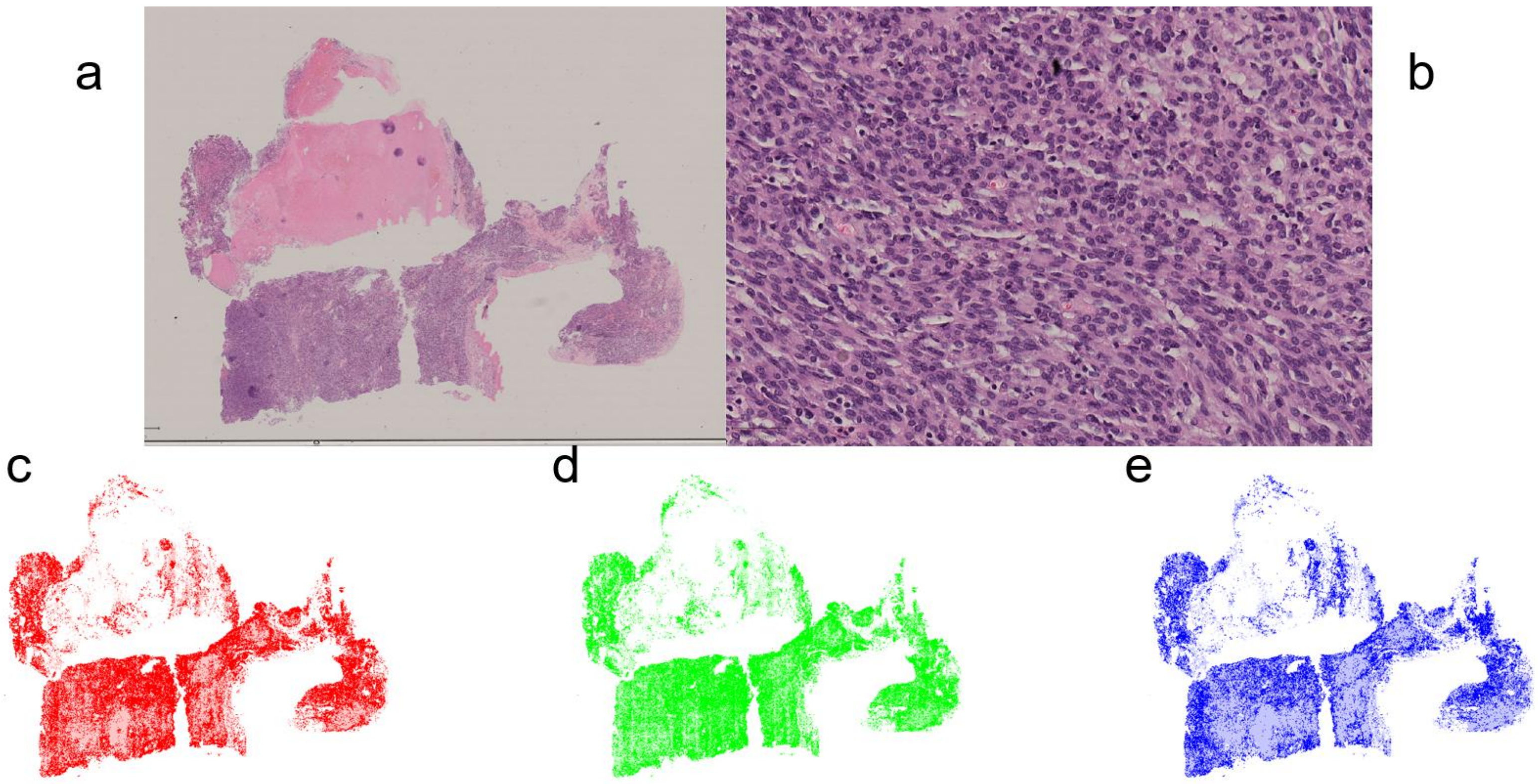
a
b
c
d
e

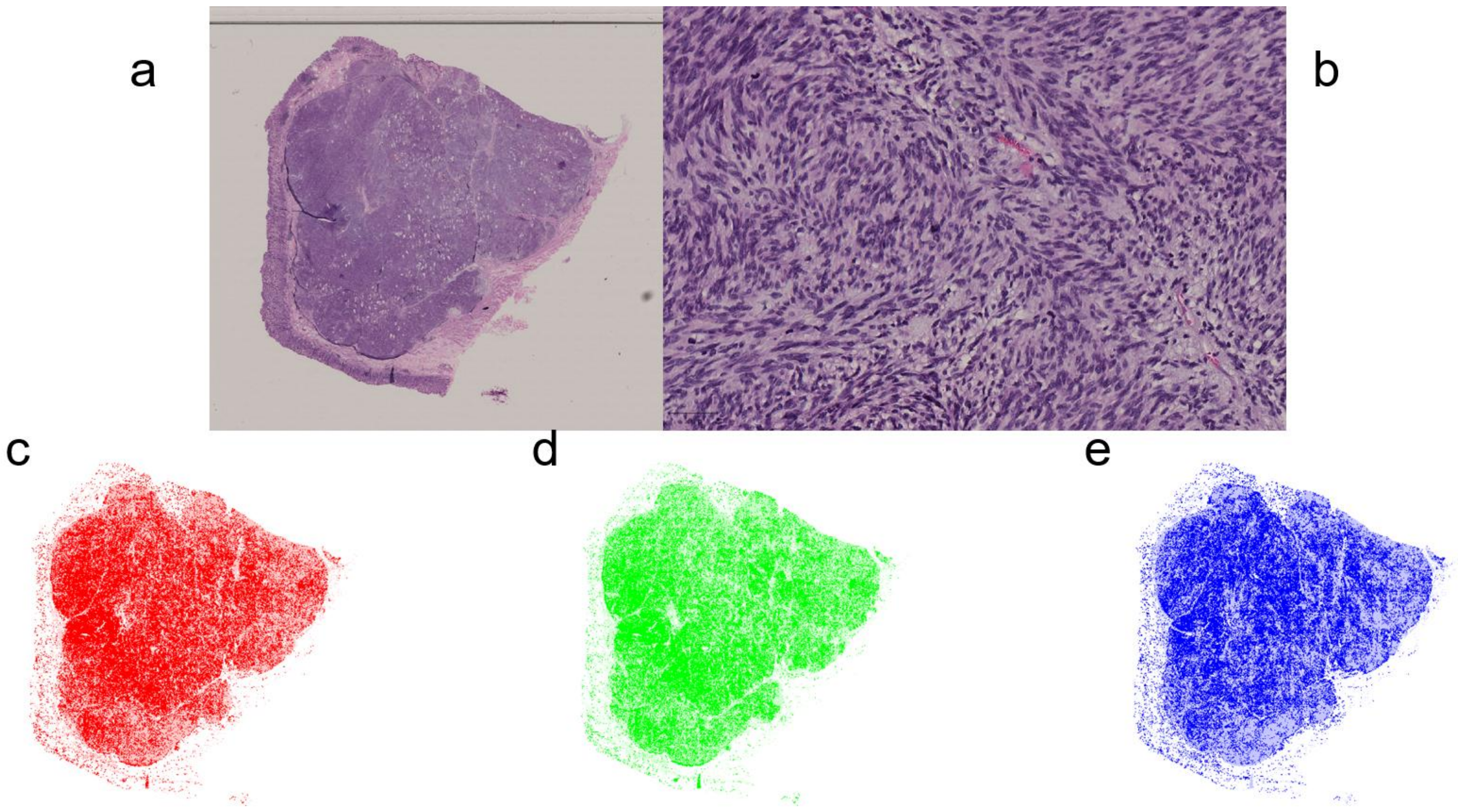
a
b
c
d
e

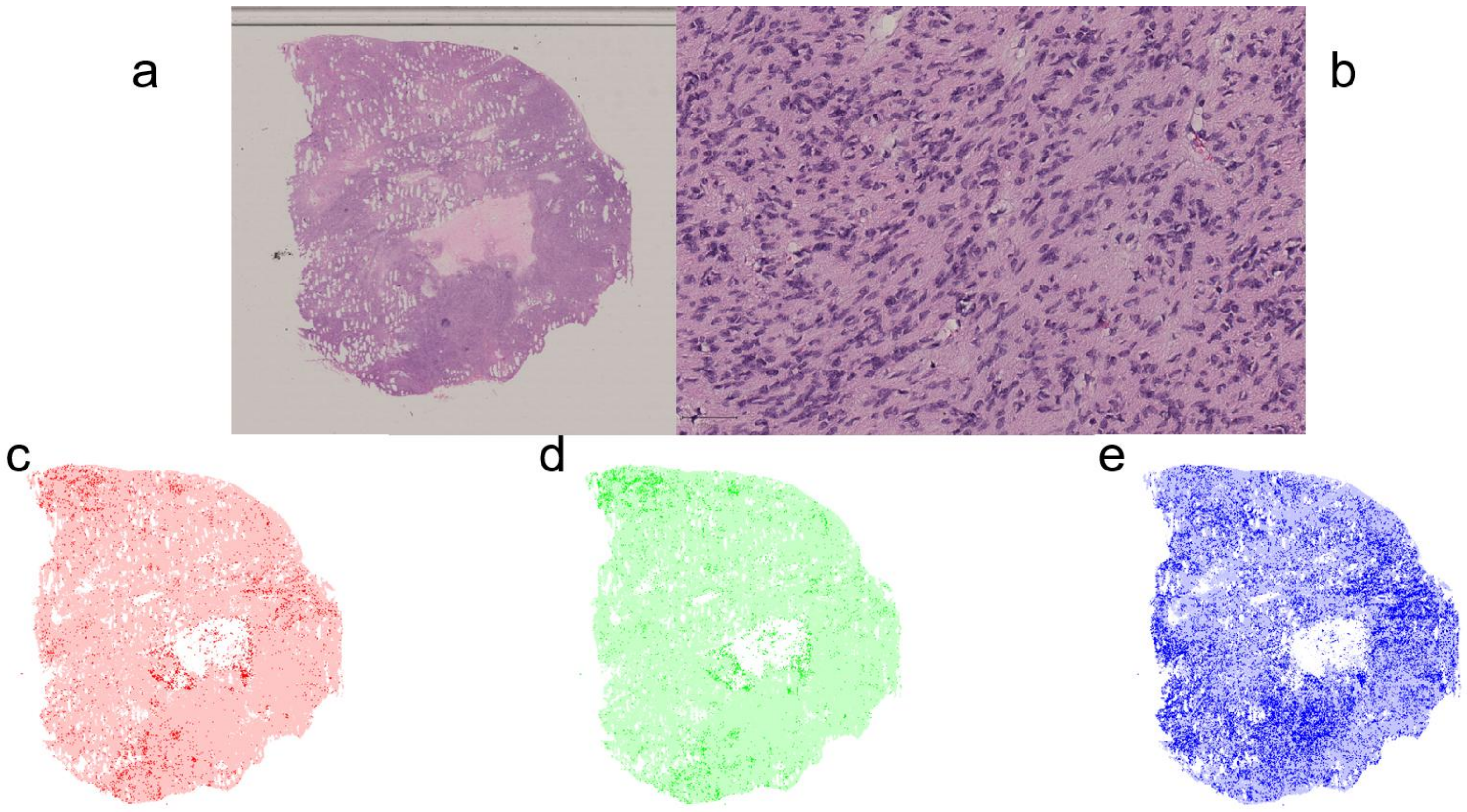
a
b
c
d
e

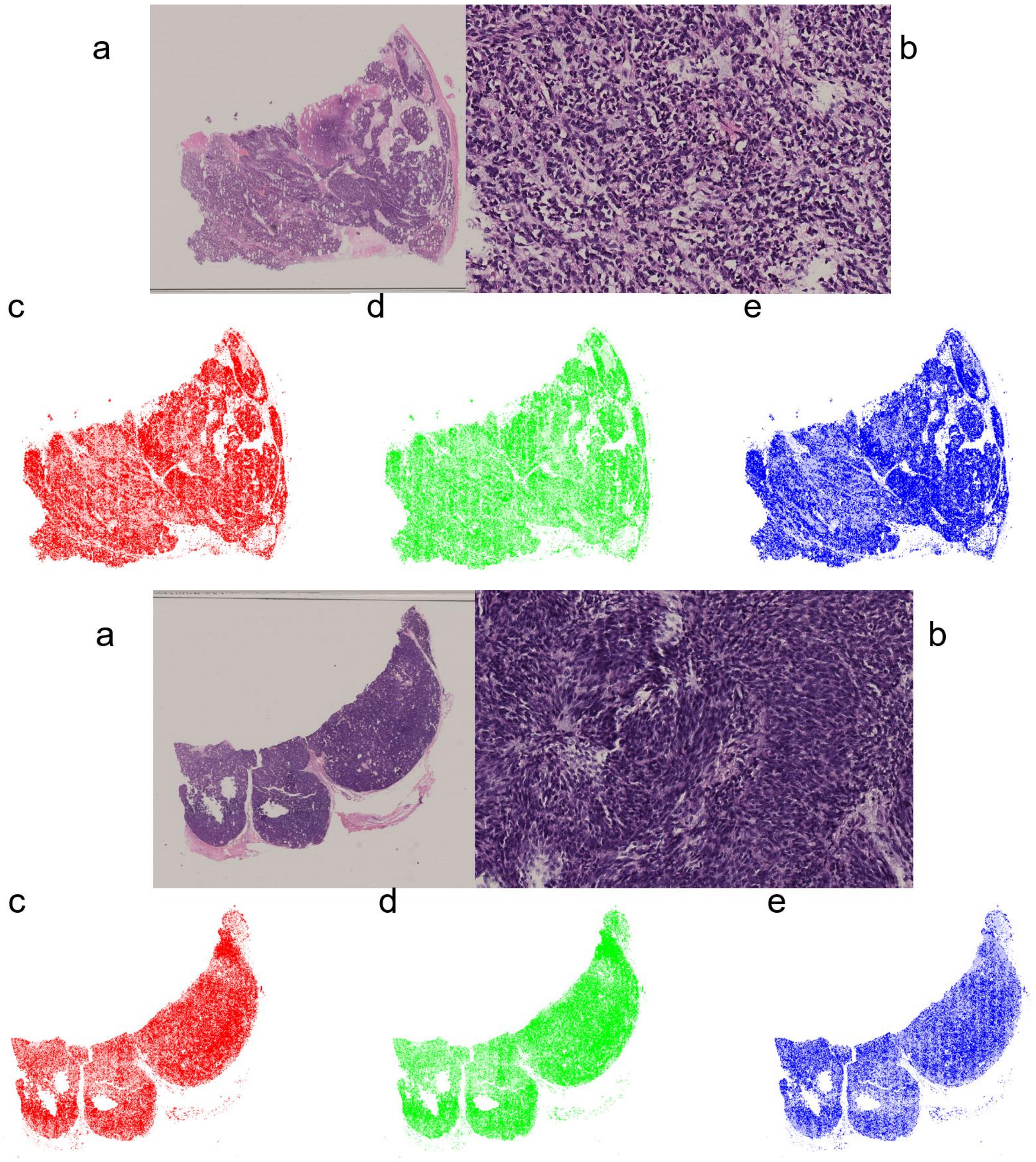

Supplementary Figure 2. Visualized prediction of pathomics deep learning model and texture distribution. (a) The overview of WSI; (b) the detailed view of WSI; (c) the distribution of the pathomics score (the darker the color, the larger the value); (d) the distribution of the cluster1 score (the darker the color, the larger the value); (e) the distribution of the cluster2 score (the darker the color, the larger the value).

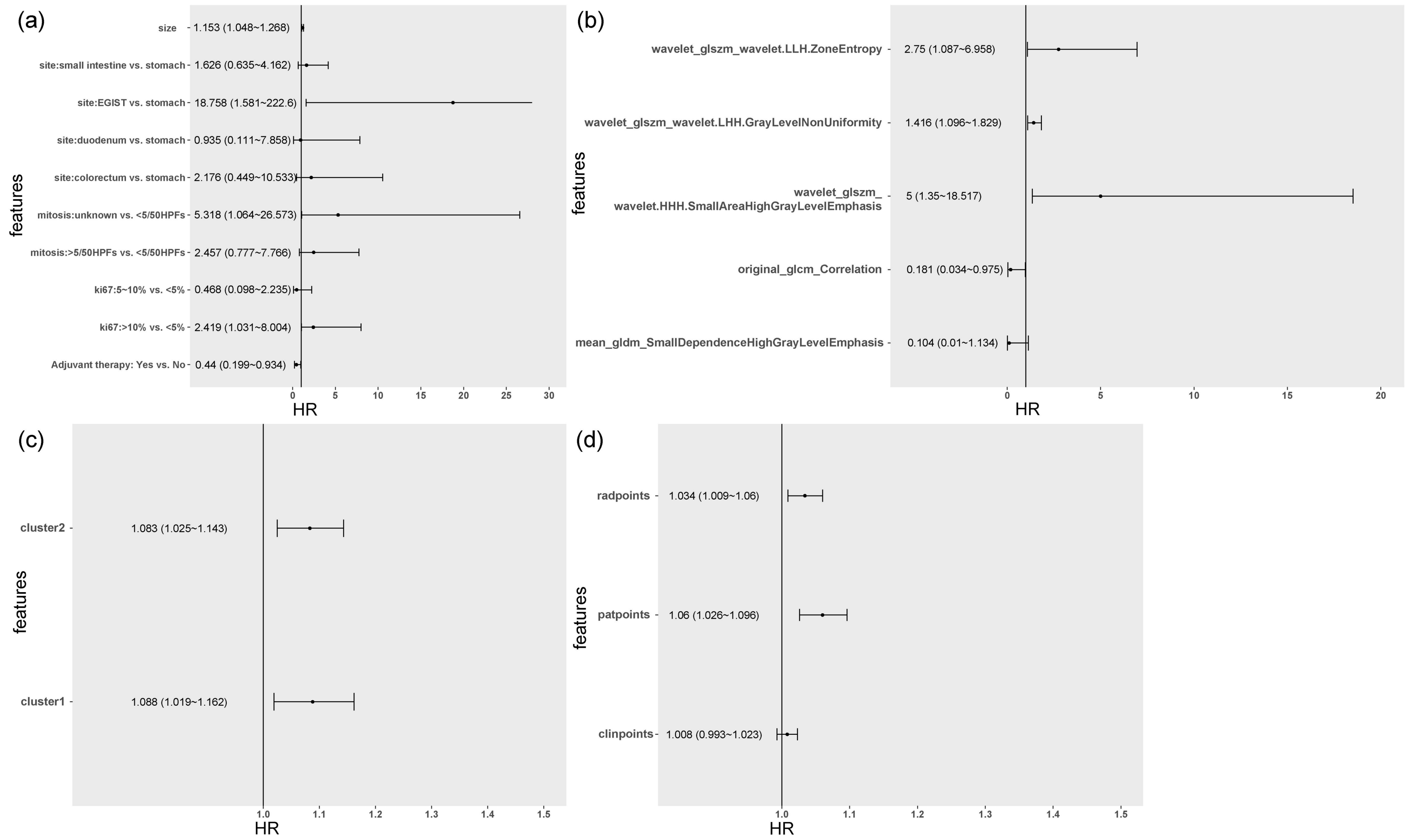

Supplementary Figure 3. Forest plots of unimodal models and the multimodal model. (a) The forest plot of clinical model; (b) the forest plot of radiomics model; (c) the forest plot of pathomics model; (d) the forest plots of the multimodal model.

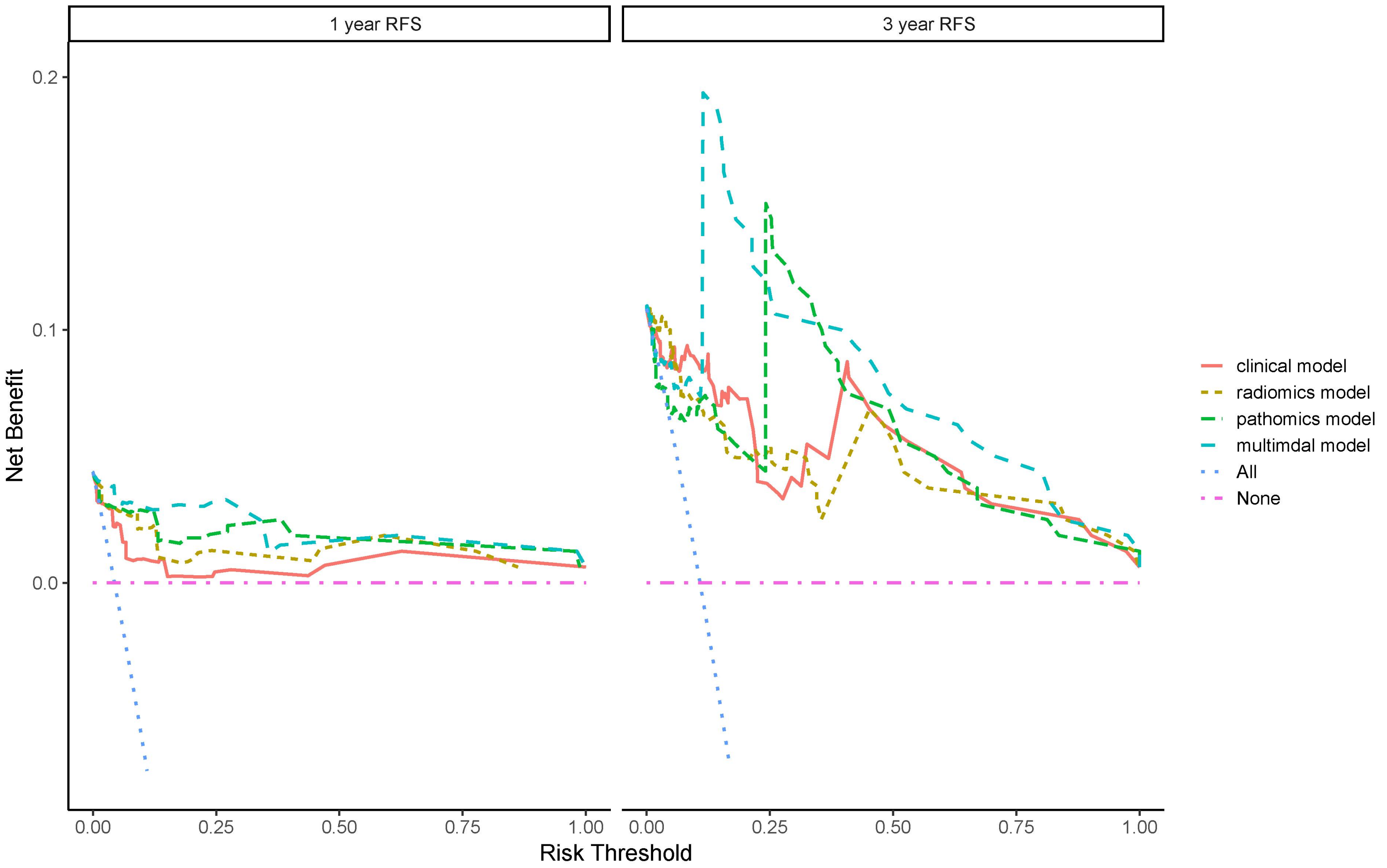

Supplementary Figure 4. DCA curves of uni-modal and multi-modal model

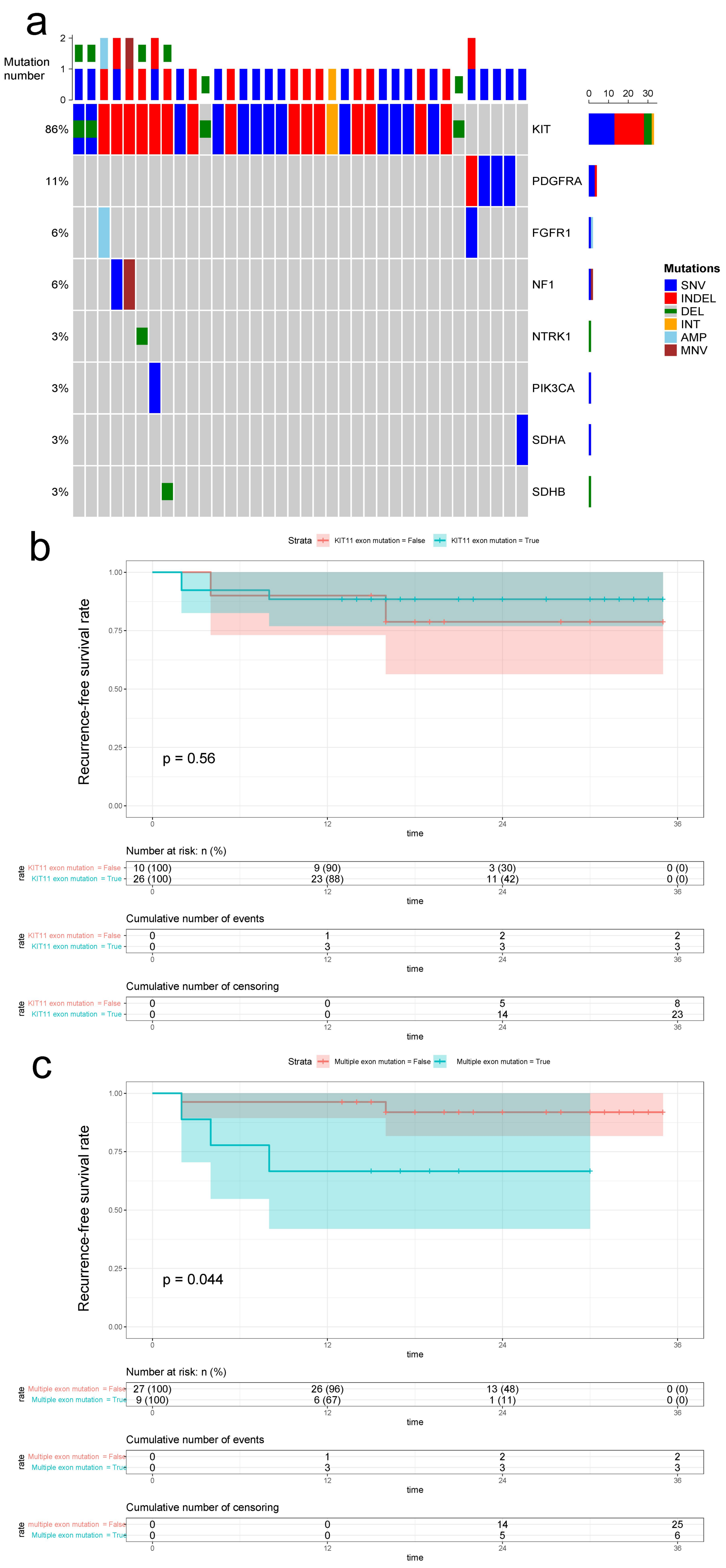

Supplementary Figure 5. Analysis of the results of targeted gene mutation detection. (a) gene mutation landscape of 36 patients who underwent targeted gene mutation detection; (b) Kaplan-Meier analysis on RFS stratified by KIT-11 mutation status (n = 36 patients); (c) Kaplan-Meier analysis on RFS stratified by multiple mutation status (n = 36 patients). P-values were calculated using the log-rank test. SNV: single nucleotide variation; INDEL: insertion/deletion mutation; DEL: deletion mutation; INT: interposition mutation; AMP: copy number amplification; MNV: multiple nucleotide variation.

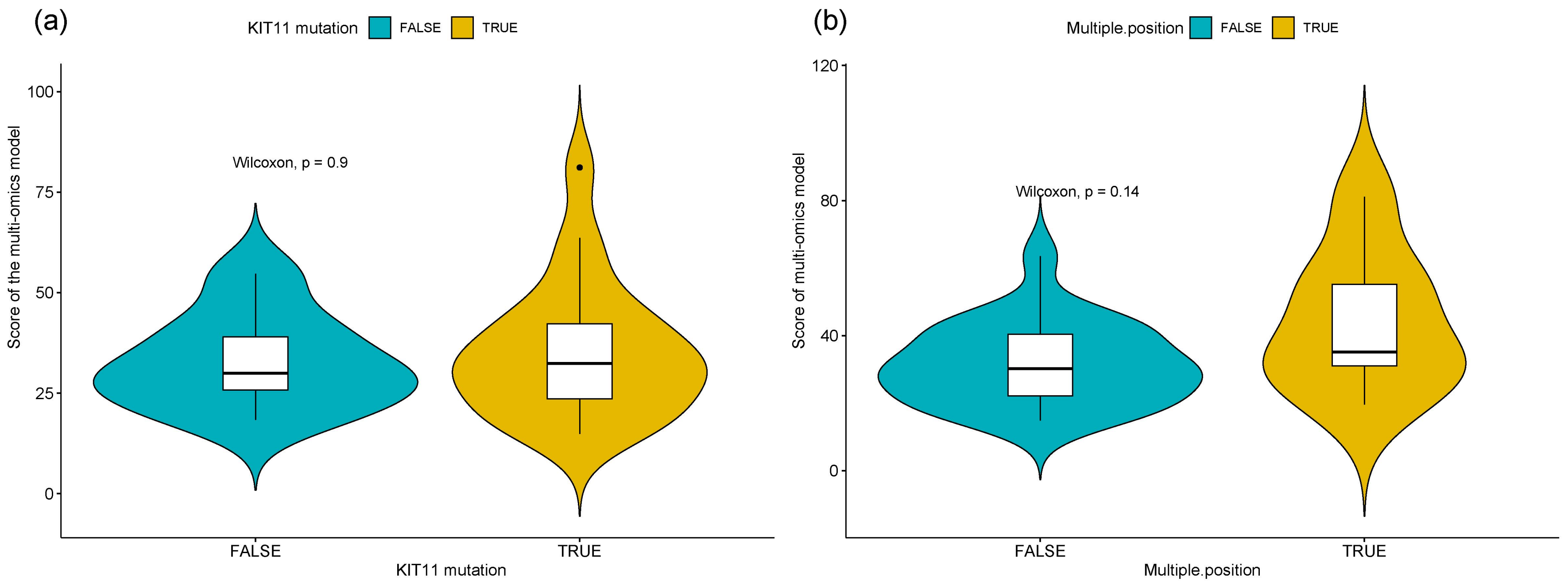

Supplementary Figure 6. The relationship between the phenotype of gene mutation and predictions of multi-omics model. (a) comparison of multimodal model's predictive scores in KIT11 mutation group and non-KIT11 mutation group; (b) comparison of multimodal model's predictive scores in multiple mutation position group and non-multiple mutation position group (n = 36 patients).

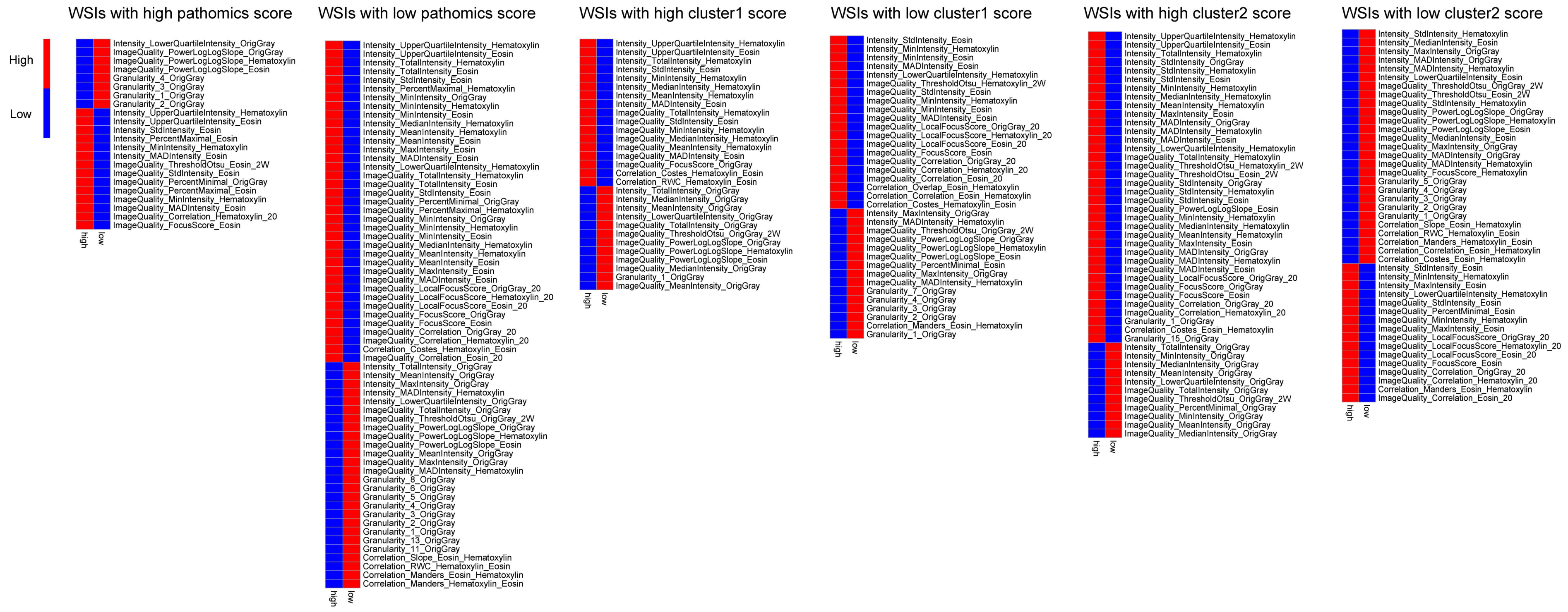

Supplementary Figure 7. The distribution of texture features categorized by different level of pathomics score, cluster1 score and cluster2 score.

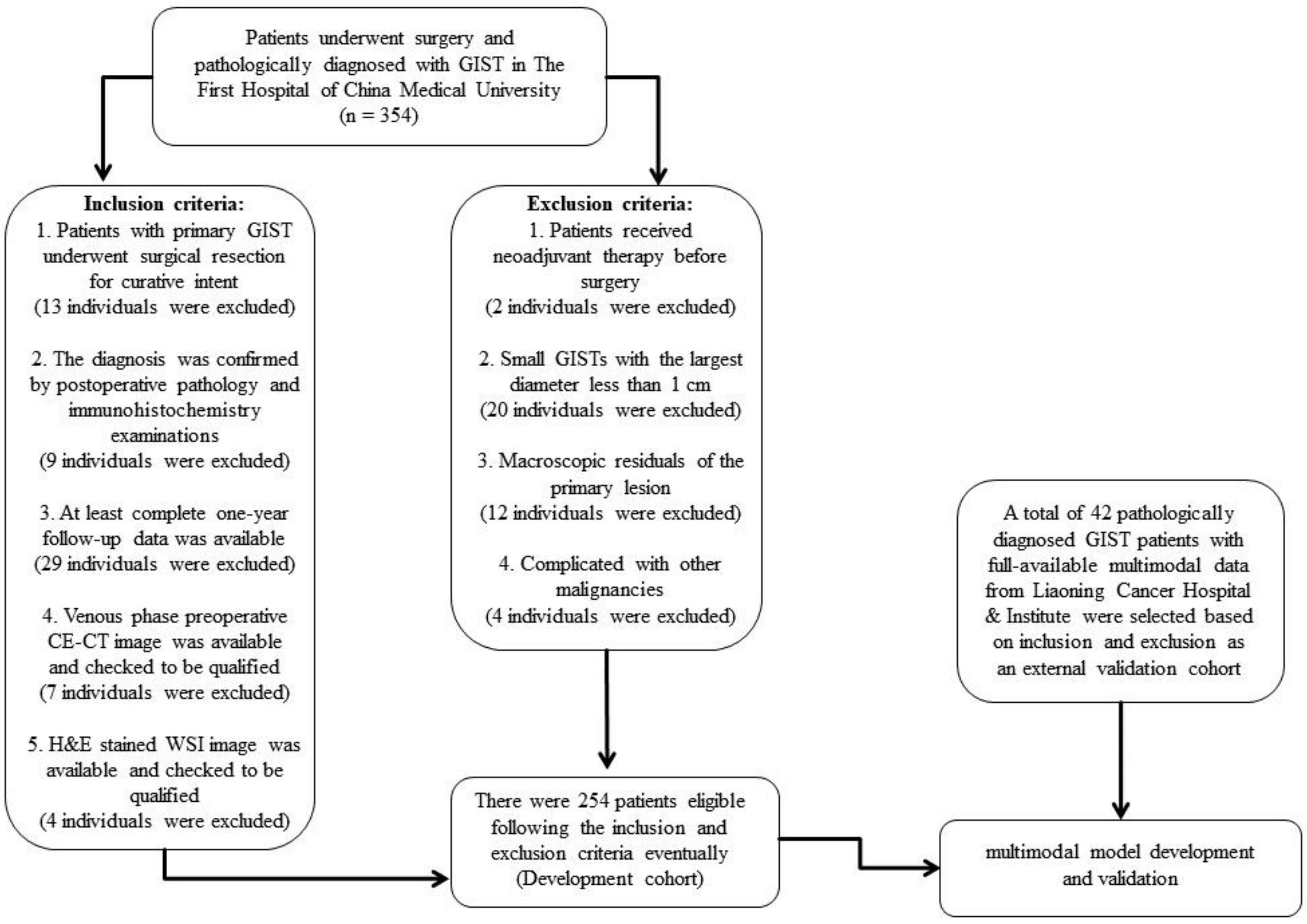

Supplementary Figure 8. Flow chart

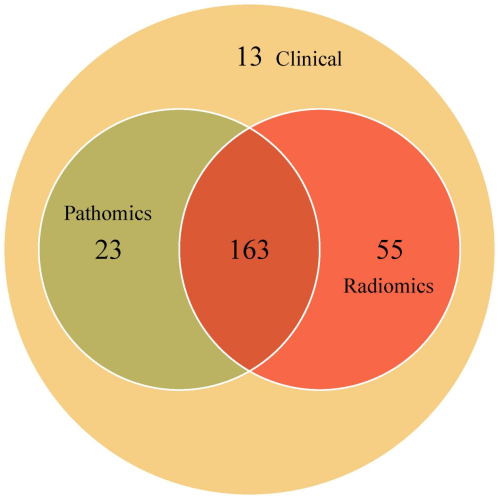

Supplementary Figure 9. Venn diagram of patients in our study with available clinical information, CE-CT images, and H&E stained images

Supplementary Table 1. Functions of prediction scores given by sub-pathomics models

Cluster1 score = (45.455*pfeature_40) + (14.113-8.821*pfeature_675) + (45.971*pfeature_1121) + (60.237*pfeature_1249-12.047) + (94.683-72.833*pfeature_1638) + (24.183-18.602*pfeature_258) + (3.018*pfeature_1782-0.603) + (12.969-6.485*pfeature_1824) + (10.402-7.43*pfeature_2041)

Cluster2 score = (34.026-34.026*pfeature_509) + (26.748-33.435*pfeature_741) + (37.418*pfeature_934-3.742) + (31.074*pfeature_1019) + (3.718*pfeature_1153-0.186) + (24.177*pfeature_1298) + (55.556-55.556*pfeature_1313) + (20.219*pfeature_1576-2.022) + (31.328*pfeature_1919-3.133) + (53.247-66.559*pfeature_285) + (11.936-18.363*pfeature_395) + (154.621*pfeature_1512-9.277)

Supplementary Table 2. The explanation of the radiomics features

Feature	Structure	Explanation
original_glcm_Correlation	original	Radiomics features extracted directly from the original images.
	glcm	Glcm: Gray Level Co-occurrence Matrix. A Gray Level Co-occurrence Matrix (GLCM) of size Ng×Ng describes the second-order joint probability function of an image region constrained by the mask and is defined as P(i,j|δ,θ). The (i,j)th element of this matrix represents the number of times the combination of levels i and j occur in two pixels in the image, that are separated by a distance of δ pixels along angle θ. The distance δ from the center voxel is defined as the distance according to the infinity norm. For δ=1, this results in 2 neighbors for each of 13 angles in 3D (26-connectivity) and for δ=2 a 98-connectivity (49 unique angles).
	Correlation	Correlation: Correlation is a value between 0 (uncorrelated) and 1 (perfectly correlated) showing the linear dependency of gray level values to their respective voxels in the GLCM.
mean_gldm_SmallDependenceHighGrayLevelEmphasis	mean	The average gray level intensity within the ROI.
	gldm	Gldm: Gray Level Dependence Matrix. A GLDM quantifies gray level dependencies in an image. A gray level dependency is defined as a the number of connected voxels within distance δ that are dependent on the center voxel. A neighbouring voxel with gray level j is considered dependent on center voxel with gray level i if |i−j|≤α. In a gray level dependence matrix P(i,j) the (i,j)th element describes the number of times a voxel with gray level i with j dependent voxels in its neighbourhood appears in image.
	SmallDependenceHighGrayLevelEmphasis	Measures the joint distribution of small dependence with higher gray-level values.
wavelet_glszm_wavelet.LLH.ZoneEntropy	wavelet	Original images were processed by wavelet filter which concluded three filters in three dimensions.
	glszm	Glszm: Gray-level size zone matrix. When glszm is wider and non-zero elements gathered in the right side, there is a rougher origin image. This is because the width of GLSZM is determined by the size of the largest connected domain, and the elements on the right side of GLSZM correspond to the number of connected domains with larger areas. The wider the GLSZM, it means that there are larger connected domains in the original image; the more non-zero elements on the right side of the GLSZM, it means that the areas of the connected domains in the original image are larger, and to the naked eye, there will be several larger areas in the image. sub-region.
	wavelet.LLH.ZoneEntropy	Zone Entropy measures the uncertainty/randomness in the distribution of zone sizes and gray levels. A higher value indicates more heterogeneneity in the texture patterns.
wavelet_glszm_wavelet.HHH.SmallAreaHighGrayLevelEmphasis	wavelet.HHH.SmallAreaHighGrayLevelEmphasis	SmallAreaHighGrayLevelEmphasis (SAHGLE) measures the proportion in the image of the joint distribution of smaller size zones with higher gray-level values.
wavelet_glszm_wavelet.LHH.GrayLevelNonUnifo	wavelet.LHH.GrayLevelNonUniformit	GrayLevelNonUniformity (GLN) measures the

rmity	y	variability of gray-level intensity values in the image, with a lower value indicating more homogeneity in intensity values.

All the explanations were gathered from Welcome to pyradiomics documentation! — pyradiomics v3.1.0rc2.post5+g6a761c4 documentation.

Supplementary Table 3. Acquisition Parameters of CT Images

Scanners	Discovery CT750 HD (GE)<br>Revolution CT (GE)<br>Aquilion (TOSHIBA)<br>iCT 256 (PHILIPS)<br>Optima CT680 Series (GE)<br>SOMATOM Definition Flash (SIEMENS)
Tube voltage	120kV
Slice thickness	1-2mm
Image matrix	512*512

Supplementary Table 4. The explanation of the pathomics features

Feature	Module	Explanation
Correlation_Correlation_Eosin_Hematoxylin	MeasureColocalization	The correlation between a pair of images Eosin and Hematoxylin, calculated as Pearson's correlation coefficient. The formula is covariance(Eosin, Hematoxylin)/[std(Eosin) × std(Hematoxylin)].
Correlation_Costes_Eosin_Hematoxylin		Costes' automated threshold estimates maximum threshold of intensity for each image based on correlation. Manders coefficient is applied on thresholded images as Eosini_coloc = Eosini when Hematoxylini > Hematoxylinthr and Hematoxylini_coloc = Hematoxylini when Eosini > Eosinthr where Hematoxylinthr and Eosinthr are thresholds calculated using Costes' automated threshold method. (i = intensity, thr = threshold)
Correlation_Costes_Hematoxylin_Eosin		Costes' automated threshold estimates maximum threshold of intensity for each image based on correlation. Manders coefficient is applied on thresholded images as Hematoxylini_coloc = Hematoxylini when Eosini > Eosinthr and Eosini_coloc = Eosini when Hematoxylini > Hematoxylinthr where Eosinthr and Hematoxylinthr are thresholds calculated using Costes' automated threshold method.
Correlation_K_Eosin_Hematoxylin		
Correlation_K_Hematoxylin_Eosin		
Correlation_Manders_Eosin_Hematoxylin		The Manders coefficient for a pair of images Eosin and Hematoxylin is measured as M1 = sum(Eosini_coloc)/sum(Eosini) and M2 = sum(Hematoxylini_coloc)/sum(Hematoxylini), where Eosini_coloc = Eosini when Hematoxylini > 0, 0 otherwise and Hematoxylini_coloc = Hematoxylini when Eosini >0, 0 otherwise.
Correlation_Manders_Hematoxylin_Eosin		The Manders coefficient for a pair of images Hmatoxylin and Eosin is measured as M1 = sum(Hematoxylini_coloc)/sum(Hematoxylini) and M2 = sum(Eosini_coloc)/sum(Eosini), where Hematoxylini_coloc = Hematoxylini when Eosini > 0, 0 otherwise and Eosini_coloc = Eosini when Hematoxylini >0, 0 otherwise.
Correlation_Overlap_Eosin_Hematoxylin		The overlap coefficient is a modification of Pearson's correlation where average intensity values of the pixels are not subtracted from the original intensity values. For a pair of images Eosin and Hematoxylin, the overlap coefficient is measured as r = sum(Eosini * Hematoxylini) / sqrt (sum(Eosini*Eosini)*sum(Hematoxylini*Hematoxylini)).
Correlation_RWC_Eosin_Hematoxylin		The RWC coefficient for a pair of images Eosin and Hematoxylin is measured as RWC1 = sum(Eosini_coloc*Wi)/sum(Eosini) and RWC2 = sum(Hematoxylini_coloc*Wi)/sum(Hematoxylini), where Wi is Weight

		defined as Wi = (Eosinmax - Di)/Eosinmax where Eosinmax is the maximum of Ranks among Eosin and Hematoxylin based on the max intensity, and Di = abs(Rank(Eosini) - Rank(Hematoxylini)) (absolute difference in ranks between Eosin and Hematoxylin) and Eosini_coloc = Eosini when Hematoxylini > 0, 0 otherwise and Hematoxylini_coloc = Hematoxylini when Eosini >0, 0 otherwise.
Correlation_RWC_Hematoxylin_Eosin		The RWC coefficient for a pair of images Hematoxylin and Eosin is measured as RWC1 = sum(Hematoxylini_coloc*Wi)/sum(Hematoxylini) and RWC2 = sum(Eosini_coloc*Wi)/sum(Eosini), where Wi is Weight defined as Wi = (Hematoxylinmax - Di)/Hematoxylinmax where Hematoxylinmax is the maximum of Ranks among Hematoxylin and Eosin based on the max intensity, and Di = abs(Rank(Hematoxylini) - Rank(Eosini)) (absolute difference in ranks between Hematoxylin and Eosin) and Hematoxylini_coloc = Hematoxylini when Eosini > 0, 0 otherwise and Eosini_coloc = Eosini when Hematoxylini >0, 0 otherwise.
Correlation_Slope_Eosin_Hematoxylin		The slope of the least-squares regression between a pair of images Eosin and Hematoxylin. Calculated using the model $A \times$ Eosin + B = Hematoxylin, where A is the slope.
Granularity_10_OrigGray<br>Granularity_11_OrigGray<br>Granularity_12_OrigGray<br>Granularity_13_OrigGray<br>Granularity_14_OrigGray<br>Granularity_1_OrigGray<br>Granularity_2_OrigGray<br>Granularity_3_OrigGray<br>Granularity_4_OrigGray<br>Granularity_5_OrigGray<br>Granularity_6_OrigGray<br>Granularity_7_OrigGray<br>Granularity_8_OrigGray<br>Granularity_9_OrigGray	MeasureGranularity	MeasureGranularity outputs spectra of size measurements of the textures in the image. Image granularity is a texture measurement that tries to fit a series of structure elements of increasing size into the texture of the image and outputs a spectrum of measures based on how well they fit. Granularity is measured as described by Ilya Ravkin [1]. Basically, MeasureGranularity: 1 - Downsamples the image (if you tell it to). This is set in Subsampling factor for granularity measurements or Subsampling factor for background reduction. 2 - Background subtracts anything larger than the radius in pixels set in Radius of structuring element. 3 - For as many times as you set in Range of the granular spectrum, it gets rid of bright areas that are only 1 pixel across, reports how much signal was lost by doing that, then repeats. i.e. The first time it removes one pixel from all bright areas in the image, (effectively deleting those that are only 1 pixel in size) and then reports what % of the signal was lost. It then takes the first-iteration image and repeats the removal and reporting (effectively reporting the amount of signal that is two pixels in size). etc
ImageQuality_Correlation_Eosin_20<br>ImageQuality_Correlation_OrigGray_20	MeasureImageQuality	A measure of the correlation of the image for a given spatial scale. This is a measure of the image spatial intensity distribution computed across sub-regions of an image for a given spatial scale [2]. If an image is blurred, the correlation between neighboring pixels becomes high, producing a high correlation value. Some care is required in selecting an appropriate spatial scale because differences in the spatial scale capture various features: moderate scales capture the blurring of intracellular features better than small scales and larger scales are more likely to reflect cell confluence than focal blur. You should select a spatial scale no bigger than the objects of interest, although you can select as many scales as desired and check empirically which is best.
ImageQuality_FocusScore_Eosin<br>ImageQuality_FocusScore_Hematoxylin<br>ImageQuality_FocusScore_OrigGray		A measure of the intensity variance across the image. This score is calculated using a normalized variance. Higher focus scores correspond to lower blurriness. More specifically, the focus score computes the intensity variance of the entire image divided by mean image intensity. Since it is tailored for auto-focusing applications, it assumes that the overall intensity and the number of objects in the image is constant, making it less useful for comparison images of different fields of view. For distinguishing extremely blurry images, however, it performs well.
ImageQuality_LocalFocusScore_Eosin_20<br>ImageQuality_LocalFocusScore_Hematoxylin_20<br>ImageQuality_LocalFocusScore_OrigGray_20		A measure of the intensity variance between image sub-regions. A local version of the Focus Score, it subdivides the image into non-overlapping tiles, computes the normalized variance for each, and takes the mean of these values as the final metric. It is potentially more useful for comparing focus between images of different fields of view, but is subject to the same caveats as the Focus Score. It can be useful in differentiating good versus badly segmented images in the cases when badly segmented images usually contain no cell objects with high background noise.
ImageQuality_MADIntensity_Eosin<br>ImageQuality_MADIntensity_Hematoxylin		Median absolute deviation (MAD) of pixel intensity values.

ImageQuality_MADIntensity_OrigGray		
ImageQuality_MaxIntensity_Eosin		Maximum of pixel intensity values.
ImageQuality_MaxIntensity_Hematoxylin		
ImageQuality_MaxIntensity_OrigGray		
ImageQuality_MeanIntensity_Eosin		Mean of pixel intensity values.
ImageQuality_MeanIntensity_Hematoxylin		
ImageQuality_MeanIntensity_OrigGray		
ImageQuality_MedianIntensity_Eosin		Median of pixel intensity values.
ImageQuality_MedianIntensity_Hematoxylin		
ImageQuality_MedianIntensity_OrigGray		
ImageQuality_MinIntensity_Eosin		Minimum of pixel intensity values.
ImageQuality_MinIntensity_Hematoxylin		
ImageQuality_MinIntensity_OrigGray		
ImageQuality_PercentMaximal_Eosin		Percent of pixels at the maximum intensity value of the image.
ImageQuality_PercentMaximal_Hematoxylin		
ImageQuality_PercentMaximal_OrigGray		
ImageQuality_PercentMinimal_Eosin		Percent of pixels at the minimum intensity value of the image.
ImageQuality_PercentMinimal_Hematoxylin		
ImageQuality_PercentMinimal_OrigGray		
ImageQuality_PowerLogLogSlope_Eosin		The slope of the image log-log power spectrum. The power spectrum contains the frequency information of the image, and the slope gives a measure of image blur. A higher slope indicates more lower frequency components, and hence more blur [3].
ImageQuality_PowerLogLogSlope_Hematoxylin		
ImageQuality_PowerLogLogSlope_OrigGray		
ImageQuality_StdIntensity_Eosin		Standard deviation of pixel intensity values.
ImageQuality_StdIntensity_Hematoxylin		
ImageQuality_StdIntensity_OrigGray		
ImageQuality_ThresholdOtsu_Eosin_2W		The automatically calculated threshold for each image for the thresholding method of choice.
ImageQuality_ThresholdOtsu_Hematoxylin_2W		
ImageQuality_ThresholdOtsu_OrigGray_2W		
ImageQuality_TotalIntensity_Eosin		Sum of all pixel intensity values.
ImageQuality_TotalIntensity_Hematoxylin		
ImageQuality_TotalIntensity_OrigGray		
Intensity_LowerQuartileIntensity_Eosin	MeasureImageIntensity	The intensity value of the pixel for which 25% of the pixels in the object have lower values.
Intensity_LowerQuartileIntensity_Hematoxylin		
Intensity_LowerQuartileIntensity_OrigGray		
Intensity_MADIntensity_Eosin		Median absolute deviation (MAD) of pixel intensity values.
Intensity_MADIntensity_Hematoxylin		
Intensity_MADIntensity_OrigGray		
Intensity_MaxIntensity_Eosin		Maximum of pixel intensity values.
Intensity_MaxIntensity_Hematoxylin		
Intensity_MaxIntensity_OrigGray		
Intensity_MeanIntensity_Eosin		Mean of pixel intensity values.
Intensity_MeanIntensity_Hematoxylin		
Intensity_MeanIntensity_OrigGray		
Intensity_MedianIntensity_Eosin		Median of pixel intensity values.
Intensity_MedianIntensity_Hematoxylin		
Intensity_MedianIntensity_OrigGray		
Intensity_MinIntensity_Eosin		Minimum of pixel intensity values.
Intensity_MinIntensity_Hematoxylin		
Intensity_MinIntensity_OrigGray		
Intensity_PercentMaximal_Eosin		Percent of pixels at the maximum intensity value of the image.
Intensity_PercentMaximal_Hematoxylin		
Intensity_PercentMaximal_OrigGray		
Intensity_StdIntensity_Eosin		Standard deviation of pixel intensity values.
Intensity_StdIntensity_Hematoxylin		
Intensity_StdIntensity_OrigGray		
Intensity_TotalIntensity_Eosin		Sum of all pixel intensity values.
Intensity_TotalIntensity_Hematoxylin		
Intensity_TotalIntensity_OrigGray		
Intensity_UpperQuartileIntensity_Eosin		The intensity value of the pixel for which 75% of the pixels in the object have lower values.
Intensity_UpperQuartileIntensity_Hematoxylin		
Intensity_UpperQuartileIntensity_OrigGray		

All the explanations were gathered from [Measurement — CellProfiler 4.2.6 documentation (cellprofiler-manual.s3.amazonaws.com)]

Supplementary Table 5. Results of PH assumption test

Model	Global p value
Radiomics	0.44
Pathomics	0.13
Clinical	0.152
Radiomics&Pathomics pre-fusion	0.17
Radiomics&Pathomics post-fusion	0.43
Clinical&Radiomics pre-fusion	0.57
Clinical&Radiomics post-fusion	0.95
Clinical&Pathomics pre-fusion	0.53
Clinical&Pathomics post-fusion	0.89
Radiomics&Pathomics&Clinical pre-fusion	0.529
Radiomics&Pathomics&Clinical post-fusion	0.93

Supplementary References

1. Ravkin I, Temov V. (1988) “Bit representation techniques and image processing”, *Applied Informatics*, v.14, pp. 41-90, Finances and Statistics, Moskow
2. Haralick RM (1979) “Statistical and structural approaches to texture” Proc. IEEE, 67(5):786-804.
3. Field DJ (1997) “Relations between the statistics of natural images and the response properties of cortical cells” *Journal of the Optical Society of America. A, Optics, image science, and vision*, 4(12):2379-94.
